# Sparse Semiparametric Discriminant Analysis for High-dimensional Zero-inflated Data

**Published:** 2025

**Authors:** Hee Cheol Chung, Yang Ni, Irina Gaynanova

**Affiliations:** Department of Mathematics and Statistics, University of North Carolina at Charlotte, Charlotte, NC 28223, USA; Department of Statistics and Data Sciences, The University of Texas at Austin, Austin, TX 78705, USA; Department of Biostatistics, University of Michigan, Ann Arbor, MI 48109, USA

**Keywords:** Latent Gaussian copula, probit regression, robust classification, sequencing data, skewed data, variable selection

## Abstract

Sequencing-based technologies provide an abundance of high-dimensional biological data sets with highly skewed and zero-inflated measurements. Despite the computational efficiency and high interpretability offered by linear classification methods, the violation of underlying distribution assumptions, driven by high skewness and zero inflation, results in invalid classification rules and interpretations. Furthermore, existing data transformation methods addressing these violations introduce ambiguity, rendering the final model and classification performance contingent on the specific transformation employed. To tackle these challenges, we propose a novel semiparametric framework for discriminant analysis based on the truncated latent Gaussian copula model. This model accommodates skewness and zero inflation, and its estimation procedure ensures robustness against data transformations. To facilitate model interpretability, we incorporate $\ell_{1}$ sparsity regularization and establish the consistency of the classification directions in high-dimensional settings. We validate our approach using human gut microbiome, breast cancer microRNA, and single-cell RNA sequencing data, highlighting its superior classification accuracy and robustness to data transformations.

## 1. Introduction

Linear methods are popular in classification analysis due to their computational efficiency and high interpretability. However, the complexity of modern high-dimensional data raises many challenges in its application. For example, microbiome, microRNA, and single-cell RNA sequencing data not only have a large number of variables relative to the sample size, but also are highly skewed and zero-inflated (Silverman et al., 2020). A large number of variables makes classical discriminant analysis less interpretable due to the lack of variable selection, and less accurate due to the singularity of the covariance matrix (Bickel and Levina, 2004). Moreover, deviations from normality such as skewness and zero-inflation challenge the underlying distribution assumptions, consequently affecting the reliability of linear discriminant analysis, particularly in its sensitivity to outliers, as noted by Hastie et al. (2009). To mitigate these violations, various data transformation methods like logarithmic, power, and log-ratio (Aitchison, 1983) transformations have been proposed. However, their application introduces increased ambiguity, as the signal variable identification and classification performance become contingent on the specific transformation employed (Ahlmann-Eltze and Huber, 2023; Weiss et al., 2017).

A rich body of work extends the classical linear discriminant analysis to high-dimensional settings. A common approach is to add regularization to the classification direction vector, e.g., the $\ell_{2}$-regularization (Guo et al., 2007) or the $\ell_{1}$-regularization (Witten and Tibshirani, 2011; Mai et al., 2012; Gaynanova et al., 2016). An alternative approach is to consider the data piling phenomenon in high dimensions, that is, observations from each class can be projected to a single point. Ahn and Marron (2010) estimate the classification direction by maximizing the distance between data piling sites, and Lee et al. (2013) regularize the degree of data piling. While these approaches improve accuracy in high-dimensional settings, they still lead to poor performance in the presence of skewness and zero inflation. Several works consider relaxations of the Gaussian assumption. Ahn et al. (2021) utilize a trace ratio formulation of Fisher’s discriminant analysis, which is more robust to violations of Gaussianity than the standard formulation. Clemmensen et al. (2011) model each class using a Gaussian mixture with subclass-specific means and common covariance matrices. Hernández and Velilla (2005) consider a fully nonparametric kernel linear discriminant analysis. Lapanowski and Gaynanova (2019) consider the optimal scoring formulation of kernel linear discriminant analysis with sparsity regularization. These methods, however, do not account for zero inflation. In sequencing data, zeros typically represent the values below sequencing detection limit (Lubbe et al., 2021) and hence treating these zeros as absolute can lead to inaccurate classification and inference. While Witten (2011) and Dong et al. (2016) explore extensions of discriminant analysis tailored for sequencing data using zero-inflated Poisson and negative binomial models, respectively, our findings reveal that the skewness prevalent in real data often exceeds the tolerance of these models. Consequently, this leads to sub-optimal classification accuracy, compelling the need for data transformations. However, it is important to note that while applied transformations effectively address distributional violations, they may inadvertently distort the original information (McKnight et al., 2019; Vandeputte et al., 2017; Lloréns-Rico et al., 2021).

In this work, we simultaneously address the issues of high dimensionality, skewness, and zero inflation by proposing a new transformation-robust semiparametric binary classification framework via the truncated latent Gaussian copula model (Yoon et al., 2020). The model accounts for zeros that are not necessarily absolute while simultaneously accommodating extreme skewness. Latent Gaussian copula models provide an elegant framework for the analysis of non-Gaussian data of (possibly) mixed types, such as skewed continuous (Liu et al., 2009), binary (Fan et al., 2017), ordinal (Quan et al., 2018; Feng and Ning, 2019), and zero-inflated (Yoon et al., 2020). These models capture dependencies among variables via the latent correlation matrix, which can be consistently estimated by inverting a bridge function (Fan et al., 2017; Quan et al., 2018; Yoon et al., 2020) that connects latent correlations to the rank-based association measure, Kendall’s τ. As such, the estimation is invariant to monotone transformations of the data. Subsequently, these models have been used for graphical model estimation (Fan et al., 2017; Feng and Ning, 2019; Yoon et al., 2019; Chung et al., 2022) and canonical correlation analysis (Yoon et al., 2020) with non-Gaussian data. However, the use of latent Gaussian copulas in the classification context has been limited. The existing approaches (Lin and Jeon, 2003; Han et al., 2013; Mai and Zou, 2015) are restricted to continuous data type, treating zeros as absolute. Furthermore, since the linear discriminant analysis model assumes class-specific means and common covariance matrix, but the means and variances are not identifiable under the Gaussian copula, Han et al. (2013) and Lin and Jeon (2003) impose additional constraints. In particular, Han et al. (2013) assume that the marginal transformations are mean- and variance-preserving. This assumption requires the methods to rely on observation-level moment estimates, which are sensitive to zero inflation and extreme values at the right tail of the distribution. We confirm this empirically in Section 5, where we show that the performance of Han et al. (2013) is highly dependent on data transformations, and is negatively affected by extreme skewness.

There are several major difficulties in adopting the truncated Gaussian copula model for discriminant analysis. The first difficulty is the identifiability of mean and variance parameters as described above, since the copula model is invariant under shifting and scaling of the bijective marginal transformations. Second, the aforementioned unsupervised problems, graphical model estimation and canonical correlation analysis, only require a consistent estimator of the latent correlation matrix. In contrast, discriminant analysis encompasses both the estimation of the classification direction (at the latent Gaussian level) and the prediction of class labels on new data (at the observed non-Gaussian level), thus requiring mapping of observed data to the latent level. For continuous-type data, accomplishing this task involves estimating the mapping from the observation level to the latent Gaussian level (Han et al., 2013; Lin and Jeon, 2003). However, for zero-inflated data, this becomes particularly challenging as the mapping from observed zeros to underlying latent Gaussian variables is not one-to-one. Thus, a new methodology is required to establish a valid classification rule under the model, and a substantially different theoretical analysis is required to establish consistency.

To address these limitations, we propose to consider a joint binary-truncated mixed copula model, where the class label is treated as a dichotomized latent Gaussian variable, and zero-inflated covariates follow the truncated Gaussian copula. The latent continuous representation of class labels is realistic in many contexts where responses are continuous in nature, even though the observed outcomes are discrete. For example, a person’s latent degree of inflammation is continuous, but the observed disease status is binary, depending on whether the degree passes a threshold of the immune system. Similarly, there is significant heterogeneity within cancer subtypes, with classifications typically based on the degree of similarity to a “prototypical subtype” rather than a strictly categorical assignment. For instance, in our second illustrative application in Section 5, the “Basal-like” breast cancer subtype is classified based on the latent similarity of a genetic profile characterized by the continuous expression of specific biomarkers (Rakha et al., 2007). At the same time, the proposed joint framework encapsulates all relationships between the class label and covariates via the joint latent correlation matrix. As a result, unlike Lin and Jeon (2003) and Han et al. (2013), our approach does not require additional conditions on the marginal transformations, and consequently, we do not rely on observation-level moment estimates. We obtain analytic approximations of posterior class probabilities under the proposed joint model, and further demonstrate that the model has an equivalent conditional linear representation on the latent Gaussian level, with the vector of coefficients being a function of full joint correlation matrix, analogous to linear regression with random Gaussian design. Since the optimal classification direction depends only on the joint latent correlation matrix, our approach is fully invariant to monotone transformations of the data. Furthermore, by adapting $\ell_{1}$-regularization, we prove that this classification direction can be consistently estimated in high-dimensional settings with the same rate as in sparse linear regression (Bickel et al., 2009). This is a highly non-trivial result as the direct application of the element-wise consistency of rank-based estimator of joint latent correlation matrix (Yoon et al., 2020) leads to a suboptimal rate. To our knowledge, the closest result is obtained by Barber and Kolar (2018), but the proof is restricted to continuous Gaussian copula as it relies on the closed form of the inverse bridge function in that setting. In contrast, for the truncated model (Yoon et al., 2020), the bridge function is significantly more complex, with no closed-form expression for its inverse. This poses new challenges for theoretical analyses, leading us to develop a different proof technique based on newly established bounds on the first and second derivatives of the inverse bridge function. Further, we propose an estimation procedure for posterior class probabilities conditional on observed measurements (zeros and non-zeros) based on Monte Carlo draws from a multivariate truncated normal distribution, which allows us to overcome the difficulty of predicting classes based on observed non-Gaussian data. Finally, we derive a Taylor approximation of the posterior probabilities, leading to a simple classification rule where the latent measurements corresponding to zeros are substituted with their conditional expectations.

In summary, from a methodological perspective, the primary novel aspects of our work are: (a) a novel framework for discriminant analysis based on truncated latent Gaussian copula models for skewed and zero-inflated data with analytic expressions of posterior probabilities under Bayes rule; (b) a principled approach for estimation of posterior probabilities using Monte Carlo methods to approximate the analytically intractable functionals of multivariate truncated normal distribution; (c) theoretical guarantees for consistency of estimated classification direction in high-dimensional setting with novel proof techniques that bound analytically intractable 2nd derivatives of inverse bridge functions, opening the path for establishing consistency in high-dimensional settings with general semiparametric Gaussian copula regression models (Dey and Zipunnikov, 2022).

From the application perspective, the numerical results on simulated and real data consistently convey that the proposed SEmiparametric Discriminant Analysis (SEDA) method is: 1) always the best-performing method on highly-skewed and zero-inflated data, with a significant margin of error improvement compared to existing approaches; 2) the performance of other methods can be significantly improved with data transformations that mitigate skewness, but the resulting misclassification errors and selected variables are dependent on the transformation choice; 3) the proposed SEDA maintains competitive or better accuracy even when accounting for transformations while being consistent in selected variables, facilitating the robustness and reproducibility of analyses.

## 2. Methodology

### 2.1 Notation

For a vector $a\in R^{p}$, we denote the $\ell_{q}$-norm, q∈[0,∞), by $\|a{\|}_{q}={\left(\sum_{j=1}^{p}{\left|a_{j}\right|}^{q}\right)}^{1/q}$ and the $\ell_{\infty }$-norm by $\|a{\|}_{\infty }={\text{max}}_{1\leq j\leq p}\left|a_{j}\right|$. For two vectors of the same size, $a,b\in R^{p}$, we write a<b to denote element-wise inequalities such that $a_{j}<b_{j}(j=1,\ldots ,p)$. The vectors $1_{p}$, $0_{p}\in R^{p}$ denote the one and zero vectors and matrices $I_{p}$, $1_{pp}\in R^{p\times p}$ denote the identity and matrix with ones. For a matrix $A\in R^{n\times p}$, $\|A{\|}_{\infty }={\text{max}}_{jk}\left|a_{jk}\right|$ denotes its $\ell_{\infty }$-norm, and for a square matrix $T\in R^{p\times p}$, |T| denotes its determinant and $\lambda_{\text{max}}(T)$ and $\lambda_{\text{min}}(T)$ denote the largest and smallest eigenvalues of T. For two functions f and g, we denote their composite function by f∘g=f(g(x)). We let 1(⋅) denote the indicator function taking the value 1 when its argument is true and 0 otherwise. For a sequence of random variables, $X_{1},\ldots ,X_{n},\ldots$, we write $X_{n}=O_{p}\left(a_{n}\right)$ if, for any ε>0, there exist M, N>0 such that $\text{Pr}\left(\left|X_{n}/a_{n}\right|>M\right)<\varepsilon$ for all n>N. We let $\Phi_{d}\left(a_{1},\ldots ,a_{d};\Sigma \right)$ and Φ(⋅) denote the d-dimensional Gaussian distribution function with zero mean and correlation matrix Σ evaluated at ${\left(a_{1},\ldots ,a_{d}\right)}^{⊤}\in R^{d}$ and the univariate standard Gaussian distribution function, respectively. We use C and $C_{i},i=1,2,\ldots$, to denote generic constants that do not depend on the sample size n, dimension p, and model parameters. The cardinality of a set 𝒮 is denoted by card(𝒮).

### 2.2 Model

Let Y∈{0,1} be a random variable corresponding to class label and $X={\left(X_{1},\ldots ,X_{p}\right)}^{⊤}\in R^{p}$ be a random vector of covariates. To accommodate possibly skewed and zero-inflated X, we propose to model X using truncated latent Gaussian copula of Yoon et al. (2020). We first review the standard Gaussian copula model, also known as the nonparanormal model (Liu et al., 2009).

**Definition 1 (Gaussian copula model)** *A random vector* $X\in R^{p}$ *satisfies the Gaussian copula model if there exist strictly increasing transformations* $f={\left\{f_{j}\right\}}_{j=1}^{p}$ *such that* ${\left(Z_{1},\ldots ,Z_{p}\right)}^{⊤}={\left\{f_{1}\left(X_{1}\right),\ldots ,f_{p}\left(X_{p}\right)\right\}}^{⊤}\sim {\text{N}}_{p}(0,\Sigma )$, *where* Σ *is a correlation matrix. We write* $X\sim {\text{NPN}}_{p}(0,\Sigma ,f)$.

While the Gaussian copula model can accommodate skewness through transformation functions ${\left\{f_{j}\right\}}_{j=1}^{p}$, it does not allow zero-inflated variables. The model of Yoon et al. (2020) allows for both zero inflation and skewness through the following extra truncation step.

**Definition 2 (Truncated latent Gaussian copula model)** *A random vector* $X\in R^{p}$ *satisfies the truncated latent Gaussian copula model if there exist a random vector* $X^{*}\sim {\text{NPN}}_{p}(0,\Sigma ,f)$ *and constants* $D_{j}>0$, j=1,…,p, *such that* $X_{j}=1\left(X_{j}^{*}>D_{j}\right)X_{j}^{*}$.

Combining the truncated Gaussian copula model for X with the latent Gaussian copula model for binary variable (Fan et al., 2017) leads to the joint model for the class label and covariates.

**Definition 3 (Binary-truncated mixed latent Gaussian copula model)** *A random vector* ${\left(Y,X^{⊤}\right)}^{⊤}\in \{0,1\}\times R^{p}$ satisfies the binary-truncated mixed latent Gaussian copula model if there exists a random vector ${\left(X_{y}^{*},X^{*⊤}\right)}^{⊤}\sim {\text{NPN}}_{1+p}(0,\Sigma ,f)$ and constants $D_{y}$, $D_{j}>0$, j=1,…,p, *such that*

(1) $$Y=1\left(X_{y}^{*}>D_{y}\right),\;X_{j}=1\left(X_{j}^{*}>D_{j}\right)X_{j}^{*},\;j=1,\ldots ,p.$$

In Model (1), the binary response Y is a dichotomized version of a latent continuous variable $X_{y}^{*}$. This latent variable serves as a technical device to derive an analytic expression for the conditional probability Pr(Y=1∣X=x) in the presence of zero-inflated covariates as we demonstrate later in (2). Latent continuous representations have been widely used to aid understanding of discrete response regression models and improve computational efficiency (Gelman et al., 2013, Ch. 16.2). In some applications, the latent representation has an intuitive interpretation. For example, a person’s latent degree of inflammation is continuous, but the observed disease status is binary, depending on whether the degree passes a threshold of the immune system or not. Similarly, the “Basal-like” breast cancer subtype is classified based on the latent similarity of a genetic profile characterized by the continuous expression of specific biomarkers (Rakha et al., 2007). While an intuitive interpretation is not always available, we use the latent continuous representation to facilitate probability modeling (Cox, 2018, Ch. 1.3), and it is not our goal to draw inference about the latent variables themselves. Additional examples of the equivalence between conditional models for discrete Y∣X and corresponding latent continuous representations can be found for the logistic regression (Holmes and Knorr-Held, 2003; Kinney and Dunson, 2007; Ma et al., 2022; O'brien and Dunson, 2004), probit regression (Albert and Chib, 1993; Chib and Greenberg, 1998; Fasano and Durante, 2022), and linear discriminant analysis (Hastie et al., 2009, Ch. 4.4).

The Bayes classification rule assigns a new observation X to class 1 if Pr(Y=1∣X)>Pr(Y=0∣X), and to class 0, otherwise. When $D_{j}=-\infty$ (no truncation) and $f_{j}$ are identities (no X transformation), the conditional model Y∣X of (1) reduces to the standard probit regression model as we demonstrate below. In this case, a standard linear model representation of Gaussian $X_{y}^{*}$ as a function of Gaussian X holds, with the vector of coefficients being a function of the full correlation matrix Σ. Further we derive the explicit form of the Bayes classification rule in the general truncated case, connecting it to the probit model based on latent Gaussian $Z_{y}^{*}$ (for response) and Z (for features).

### 2.3 Classification Rule

We first consider the special case of model (1) with $D_{j}=-\infty$, j=1,…,p; that is, X follows standard Gaussian copula (without truncation). By definition, there exists a latent Gaussian vector ${\left(Z_{y},Z_{1},\ldots ,Z_{p}\right)}^{⊤}\sim {\text{N}}_{1+p}(0,\Sigma )$ such that $Y=1\left(X_{y}^{*}>D_{y}\right)=1\left(Z_{y}>\Delta_{y}\right)$, $\Delta_{y}=f_{y}\left(D_{y}\right)$, and $X_{j}=f_{j}^{-1}\left(Z_{j}\right)$. Let $Z={\left(Z_{1},\ldots ,Z_{p}\right)}^{⊤}$. Since $f_{j}$’s are strictly increasing, conditional on X, we have the following probit regression model:

$$\text{Pr}(Y=1|X)=\text{Pr}\left(Z_{y}>\Delta_{y}|Z\right)=\text{Pr}\left(\left.\frac{Z_{y}-\beta^{*⊤}Z}{v}>\frac{\Delta_{y}-\beta^{*⊤}Z}{v}\right|Z\right)\;=\Phi \left(\frac{\beta^{*⊤}Z-\Delta_{y}}{v}\right),$$

where $\beta^{*}=\Sigma_{22}^{-1}\Sigma_{21}$, $v^{2}=1-\Sigma_{21}^{⊤}\Sigma_{22}^{-1}\Sigma_{21}$, $\Sigma_{21}^{⊤}=\text{cov}\left(Z_{y},Z\right)$, and $\Sigma_{22}=\text{cov}(Z)$. Observe that the above representation is based on the alternative equivalent formulation of joint distribution ${\left(Z_{y},Z_{1},\ldots ,Z_{p}\right)}^{⊤}\sim {\text{N}}_{1+p}(0,\Sigma )$ through the hierarchical conditional representation $Z_{y}|Z\sim \text{N}\left(\beta^{*⊤}Z,v^{2}\right)$, where $Z\sim {\text{N}}_{p}\left(0,\Sigma_{22}\right)$. The latter is equivalent to a standard linear regression model with a random Gaussian design. Thus, under model (1), the effect of Z on $Z_{y}$ is linear and determined by the correlation structure between $Z_{y}$ and Z, the same as in the Gaussian copula regression with continuous response (Cai and Zhang, 2018). Accordingly, we have the linear Bayes classifier $\delta (X)=1\left\{\beta^{*⊤}f(X)-\Delta_{y}>0\right\}$, where $f(X)={\left\{f_{1}\left(X_{1}\right),\ldots ,f_{p}\left(X_{p}\right)\right\}}^{⊤}$.

We now consider the general truncated case, where $X_{j}=1\left(Z_{j}>\Delta_{j}\right)f_{j}^{-1}\left(Z_{j}\right)$ with $\Delta_{j}=f_{j}\left(D_{j}\right),j=1,\ldots ,p$. While the same linear relationship holds between latent $Z_{y}$ and Z as demonstrated above, the posterior probability expression is more complex due to a lack of one-to-one mapping between observed zeros and latent Z. We next derive the full expression of posterior probability under this more challenging setting, which subsequently allows us to derive tractable analytic approximations of Pr(Y=1∣X) for estimation.

For a given vector of covariates $X\in R^{p}$, let $X_{t}\in R^{p_{t}}$ and $X_{o}\in R^{p_{o}}$ be the subvectors with truncated and observed realizations, respectively, where $p_{t}+p_{o}=p$. Likewise, let $Z_{t}$ and $Z_{o}$ be the corresponding latent Gaussian vectors, and $\Delta_{t}$ and $\Delta_{o}$ be the corresponding threshold vectors. Then it follows that $\text{Pr}(Y=1|X)=\text{Pr}\left(Z_{y}>\Delta_{y}|Z_{o},Z_{t}<\Delta_{t}\right)$. Since $Z_{y}|Z\sim \text{N}\left(\beta^{*⊤}Z,v^{2}\right)$ as before, the posterior probability can be expressed by integrating out $Z_{y}$ from the model:

(2) $$\text{Pr}(Y=1|X=x)=\text{Pr}\left(Y=1|X_{o}=x_{o},X_{t}=0_{p_{t}}\right)=\text{Pr}\left(Z_{y}>\Delta_{y}|Z_{o}=z_{o},Z_{t}<\Delta_{t}\right)\;={\left\{\text{Pr}\left(Z_{t}<\Delta_{t}|Z_{o}=z_{o}\right)\right\}}^{-1}\int_{\Delta_{y}}^{\infty }\int_{z_{t}<\Delta_{t}}p\left(z_{y},z_{t}|z_{o}\right)\text{d}z_{t}\text{d}z_{y}\;={\left\{\text{Pr}\left(Z_{t}<\Delta_{t}|Z_{o}=z_{o}\right)\right\}}^{-1}\int_{z_{t}<\Delta_{t}}\left\{\int_{\Delta_{y}}^{\infty }p\left(z_{y}|z_{t},z_{o}\right)\text{d}z_{y}\right\}p\left(z_{t}|z_{o}\right)\text{d}z_{t}\;={\left\{\text{Pr}\left(Z_{t}<\Delta_{t}|Z_{o}=z_{o}\right)\right\}}^{-1}\int_{z_{t}<\Delta_{t}}\Phi \left(\frac{\beta_{t}^{*⊤}z_{t}+\beta_{o}^{*⊤}z_{o}-\Delta_{y}}{v}\right)p\left(z_{t}|z_{o}\right)\text{d}z_{t}\;=\text{E}\left\{\left.\Phi \left(\frac{\beta_{t}^{*⊤}Z_{t}+\beta_{o}^{*⊤}z_{o}-\Delta_{y}}{v}\right)\right.|Z_{o}=z_{o},Z_{t}<\Delta_{t}\right\},$$

where $\beta_{t}^{*}$ and $\beta_{o}^{*}$ are the subvectors of $\beta^{*}$ corresponding to the components of $Z_{t}$ and $Z_{o}$, respectively, and the expectation (2) is over the multivariate Gaussian distribution of $Z_{t}$ given $Z_{o}=z_{o}$ truncated to the region $\left\{a\in R^{p_{t}}|a<\Delta_{t}\right\}$. The Bayes classifier under model (1) relies solely on the conditional model (2), assigning a new observation X to class 1 if the expectation in (2) exceeds 0.5, and to class 0 otherwise.

The conditional expectation in (2) does not admit a closed form, and we consider two approaches for its approximation. First, we can use Monte Carlo sampling. Let ${\left\{z_{t}^{\left(s\right)}\right\}}_{s=1}^{S}$ be an independent sample of size S from the $p_{t}$-variate truncated Gaussian, then

(3) $$\text{E}\left\{\left.\Phi \left(\frac{\beta_{t}^{*⊤}Z_{t}+\beta_{o}^{*⊤}z_{o}-\Delta_{y}}{v}\right)\right.|Z_{o}=z_{o},Z_{t}<\Delta_{t}\right\}\approx \frac{1}{S}\sum_{s=1}^{S}\Phi \left(\frac{\beta_{t}^{*⊤}z_{t}^{\left(s\right)}+\beta_{o}^{*⊤}z_{o}-\Delta_{y}}{v}\right).$$

This approach, however, is computationally demanding and makes the classification rule dependent on the scalar v (recall that the classification rules of probit and standard Gaussian copula model do not depend on v). Secondly, we consider the first-order Taylor approximation of (2) around the mean $\mu_{t}=\text{E}\left(Z_{t}|Z_{o}=z_{o},Z_{t}<\Delta_{t}\right)$, which leads to

(4) $$\text{E}\left\{\left.\Phi \left(\frac{\beta_{t}^{*⊤}Z_{t}+\beta_{o}^{*⊤}z_{o}-\Delta_{y}}{v}\right)\right.|Z_{o}=z_{o},Z_{t}<\Delta_{t}\right\}\approx \Phi \left(\frac{\beta_{t}^{*⊤}\mu_{t}+\beta_{o}^{*⊤}z_{o}-\Delta_{y}}{v}\right),$$

where the derivation is given in Appendix A. The mean of multivariate truncated Gaussian distribution $\mu_{t}$ still needs to be estimated with the Monte Carlo sample. However, the classification rule based on (4) is linear, $\delta (X)=1\left\{\beta_{t}^{*⊤}\mu_{t}+\beta_{o}^{*⊤}f_{o}\left(X_{o}\right)-\Delta_{y}>0\right\}$, so its main advantage over (3) is that it does not require multiple evaluations of the standard normal distribution function and the estimation of the scaling factor v.

### 2.4 Estimation of the Classification Direction

The Bayes classification rule under model (1) depends crucially on $\beta^{*}=\Sigma_{22}^{-1}\Sigma_{21}\in R^{p}$, which we refer to as classification direction. The best linear unbiased predictor for $Z_{y}$, the latent Gaussian variable of the class label, is $\text{E}\left(Z_{y}|Z\right)=\beta^{*⊤}Z$, where $\beta^{*}$ is the minimizer of the mean squared error criterion:

(5) $$\beta^{*}=\underset{\beta \in R^{p}}{\text{argmin}}\;\text{E}\left\{{\left(Z_{y}-\beta^{⊤}Z\right)}^{2}\right\}=\underset{\beta \in R^{p}}{\text{argmin}}\;\left(\beta^{⊤}\Sigma_{22}\beta -2\beta^{⊤}\Sigma_{12}\right).$$

In practice, $\Sigma_{12}$ and $\Sigma_{22}$ need to be estimated from the data. However, as $Z_{y}$ and Z are unobservable latent variables, $\Sigma_{21}$ and $\Sigma_{22}$ cannot be directly estimated using the sample. Instead, we propose to utilize rank-based estimators for $\Sigma_{12}$ and $\Sigma_{22}$ that take advantage of the bridge function connecting latent correlations to Kendall’s τ values (Fan et al., 2017; Yoon et al., 2020). The advantage of this connection is that it enables consistent estimation of latent correlations based on ranks without requiring estimation of underlying monotone transformations $f_{j}$’s.

Specifically, a strictly increasing bridge function G is defined such that $G\left(\Sigma_{jk}\right)=\text{E}\left({\overset{ˆ}{\tau }}_{jk}\right)=\tau_{jk}$, where $\Sigma_{jk}$ is an element of the full correlation matrix Σ corresponding to $Z_{j}$ and $Z_{k}$, $\tau_{jk}$ is the corresponding population Kendall’s τ, and ${\overset{ˆ}{\tau }}_{jk}$ is the sample Kendall’s τ. The sample Kendall’s τ is defined as

(6) $${\overset{ˆ}{\tau }}_{jk}=\frac{2}{n(n-1)}\sum_{1\leq i\leq i^{\prime }\leq n}\text{sign}\left(X_{ij}-X_{i^{\prime }j}\right)\text{sign}\left(X_{ik}-X_{i^{\prime }k}\right),$$

where $X_{ij}$ is the ith independent sample of $X_{j}$, n is the sample size. The specific form of the bridge function G depends on the type of observed variables. We are interested in binary-truncated (BT) pairs (correlations between the binary class label and zero-inflated variables) and truncated-truncated (TT) pairs (correlations between zero-inflated variables). The corresponding bridge functions $G_{BT}$ and $G_{TT}$ have the closed form expressions (Yoon et al., 2020):

(7) $$G_{BT}\left(\Sigma_{jk};\Delta_{j},\Delta_{k}\right)=2\left\{1-\Phi \left(\Delta_{j}\right)\right\}\Phi \left(\Delta_{k}\right)-2\Phi_{3}\left(-\Delta_{j},-\Delta_{k},0;\Sigma_{3a}\right)\;-2\Phi_{3}\left(-\Delta_{j},-\Delta_{k},0;\Sigma_{3b}\right),\;G_{TT}\left(\Sigma_{jk};\Delta_{j},\Delta_{k}\right)=-2\Phi_{4}\left(-\Delta_{j},-\Delta_{k},0,0;\Sigma_{4a}\right)+2\Phi_{4}\left(-\Delta_{j},-\Delta_{k},0,0;\Sigma_{4b}\right),$$

with

$$\Sigma_{3a}=\left(\begin{array}{ccc} 1 & -r & 1/\surd 2\\ -r & 1 & -r/\surd 2\\ 1/\surd 2 & -r/\surd 2 & 1 \end{array}\right),\;\Sigma_{3b}=\left(\begin{array}{ccc} 1 & 0 & -1/\surd 2\\ 0 & 1 & -r/\surd 2\\ -1/\surd 2 & -r/\surd 2 & 1 \end{array}\right),$$

and

$$\Sigma_{4a}=\left(\begin{array}{cccc} 1 & 0 & 1/\surd 2 & -r/\surd 2\\ 0 & 1 & -r/\surd 2 & 1/\surd 2\\ 1/\surd 2 & -r/\surd 2 & 1 & -r\\ -r/\surd 2 & 1/\surd 2 & -r & 1 \end{array}\right),\;\Sigma_{4b}=\left(\begin{array}{cccc} 1 & r & 1/\surd 2 & r/\surd 2\\ r & 1 & r/\surd 2 & 1/\surd 2\\ r/\surd 2 & 1/\surd 2 & 1 & r\\ 1/\surd 2 & r/\surd 2 & r & 1 \end{array}\right),$$

where $r=\Sigma_{jk}$. The moment equation $G\left(\Sigma_{jk};\Delta_{j},\Delta_{k}\right)=\text{E}\left({\overset{ˆ}{\tau }}_{jk}\right)$ and the strict monotonicity of G enable estimation of the latent correlation matrix Σ using the method of moments. We use the moment estimator ${\overset{ˆ}{\Delta }}_{j}=\Phi^{-1}\left({\overset{ˆ}{\pi }}_{j}\right)$, where ${\overset{ˆ}{\pi }}_{j}=\sum_{i=1}^{n}1\left(x_{ij}=0\right)/n$ is the proportion of zeros in $X_{ij},i=1,\ldots ,n$, leading to ${\overset{ˆ}{\Sigma }}_{jk}=G^{-1}\left({\overset{ˆ}{\tau }}_{jk};{\overset{ˆ}{\Delta }}_{j},{\overset{ˆ}{\Delta }}_{k}\right)$. At the sample level, however, $\overset{ˆ}{\Sigma }={\left[{\overset{ˆ}{\Sigma }}_{jk}\right]}_{1\leq j,k\leq p}$ is not guaranteed to be positive-definite (Fan et al., 2017; Yoon and Gaynanova, 2021). Yoon et al. (2020) propose to use $\tilde{\Sigma }=(1-\nu ){\overset{ˆ}{\Sigma }}_{*}+\nu I_{p}$ with a small positive constant ν to ensure the positive definiteness, where ${\overset{ˆ}{\Sigma }}_{*}$ is the projection of $\overset{ˆ}{\Sigma }$ on the cone of positive-semidefinite matrices. When $\nu =o\left\{(\text{log}\;p/n{)}^{1/2}\right\}$, $\tilde{\Sigma }$ has the same consistency rates as $\overset{ˆ}{\Sigma }$. We refer to Corollary 2 in Fan et al. (2017) and Theorem 7 in Yoon et al. (2020). In our implementation, we set $\overset{ˆ}{\Sigma }=\Sigma$ using the R package mixedCCA (Yoon and Gaynanova, 2021), which uses the default value of ν=0.01.

**Remark 4** *The sample Kendall’s* τ *in* (6) *is also known as* $\tau^{a}$, *and it ignores ties since the sign function is defined as* sign(0)=0. *The closed-form derivations of bridge functions and estimation consistency results mentioned above apply to* $\tau^{a}$. *To account for ties, one can consider an alternative Kendall’s* $\tau^{b}$ (Quan et al., 2018), *but the updated bridge functions require significantly more complex derivations and do not admit closed-form expression, thus we restrict our focus to $\tau^{a}$ in this work*.

In summary, to estimate $\beta^{*}$, we propose to replace $\Sigma_{21}$ and $\Sigma_{22}$ in (5) with the corresponding rank-based estimators ${\overset{ˆ}{\Sigma }}_{21}$ and ${\overset{ˆ}{\Sigma }}_{22}$, respectively. In addition, we consider a $\ell_{1}$–regularization to account for high dimensionality and to enhance the interpretability of the resulting classification rule. Specifically, we consider the following minimization problem:

(8) $$\overset{ˆ}{\beta }={\text{argmin}}_{\beta \in R^{p}}\left(\frac{1}{2}\beta^{⊤}{\overset{ˆ}{\Sigma }}_{22}\beta -\beta^{⊤}{\overset{ˆ}{\Sigma }}_{21}+\lambda \|\beta {\|}_{1}\right),$$

where λ>0 is the tuning parameter that controls the sparsity level of $\overset{ˆ}{\beta }$. This convex optimization problem can be efficiently solved via the coordinate descent algorithm. In our numerical studies, we select the tuning parameter λ that yields the lowest misclassification rate through 5-fold cross-validation.

### 2.5 Estimation of the Classification Rule

Here we describe how to obtain the sample Bayes classification rule based on the optimal rule in Section 2.3 and estimated classification direction $\overset{ˆ}{\beta }$. The critical difficulty is that both approximations of the posterior probability (3) and (4) rely on the latent Z, which is unobservable. We first illustrate how to estimate $Z_{o}$, the subvector of Z corresponding to non-zero observed values in X. We then use the estimated $Z_{o}$ to generate posterior samples of $Z_{t}$, the subvector of Z corresponding to zero values in X, for use in classification rule (3), and in computing the conditional mean for the classification rule (4).

Let ${\left(Y_{i},X_{i}^{⊤}\right)}^{⊤}={\left(Y_{i},X_{i1},\ldots ,X_{ip}\right)}^{⊤}\in \{0,1\}\times R^{p}$, i=1,…,n, be the ith sample from the latent Gaussian copula model for binary-truncated mixed data as in Definition 3. For each i, we write $X_{i,t}$ and $X_{i,o}$ to denote the truncated and observed subvectors of $X_{i}\in R^{p}$, respectively. Similarly, we denote the truncated and observed subvectors of a new observation $X^{\text{new}}$ by $X_{t}^{\text{new}}\in R^{p_{t}}$ and $X_{o}^{\text{new}}\in R^{p_{o}}$. We write ${\overset{ˆ}{\Delta }}_{y}$ and $\overset{ˆ}{\Delta }={\left({\overset{ˆ}{\Delta }}_{1},\ldots ,{\overset{ˆ}{\Delta }}_{p}\right)}^{⊤}$ to denote the estimated thresholds, where ${\overset{ˆ}{\Delta }}_{y}=\Phi^{-1}\left({\overset{ˆ}{\pi }}_{y}\right)$ and ${\overset{ˆ}{\Delta }}_{j}=\Phi^{-1}\left({\overset{ˆ}{\pi }}_{j}\right)$ with ${\overset{ˆ}{\pi }}_{j}=\sum_{i=1}^{n}1\left(x_{ij}=0\right)/n$, and ${\overset{ˆ}{\pi }}_{y}=\sum_{i=1}^{n}1\left(y_{i}=0\right)/n$.

To estimate $z_{o}^{\text{new}}$ corresponding to observed $x_{o}^{\text{new}}$, recall from Definition 3 that $z_{j,o}^{\text{new}}$ is given by $z_{j,o}^{\text{new}}=f_{j}\left(x_{j,o}^{\text{new}}\right)=\Phi^{-1}\circ F_{j}\left(x_{j,o}^{\text{new}}\right)$, where $F_{j}$ is the marginal distribution function of the jth latent variable $X_{j}^{*}$ (Liu et al., 2009). We propose to estimate $F_{j}$ using the empirical cumulative distribution function, where we apply the winsorization similar to Han et al. (2013) to avoid $f_{j}\left(x_{j,o}^{\text{new}}\right)$ being infinite. Based on observations $x_{1j},\ldots ,x_{nj}$, we consider

$${\overset{ˆ}{F}}_{j}\left(t;\delta_{n},x_{1j},\ldots ,x_{nj}\right)=W_{j}^{\delta_{n}}\left\{\frac{1}{n}\sum_{i=1}^{n}1\left(x_{ij}\leq t\right)\right\},$$

where

$$W_{j}^{\delta_{n}}(x)={\overset{ˆ}{\pi }}_{j}1\left(x\leq {\overset{ˆ}{\pi }}_{j}\right)+x1\left({\overset{ˆ}{\pi }}_{j}<x\leq 1-\delta_{n}\right)+\left(1-\delta_{n}\right)1\left(1-\delta_{n}<x\right).$$

In our numerical studies, we use $\delta_{n}=1/(2n)$ as recommended by Han et al. (2013). Based on ${\overset{ˆ}{F}}_{j}$’s, we set ${\overset{ˆ}{f}}_{j}=\Phi^{-1}\circ {\overset{ˆ}{F}}_{j}$ and estimate $z_{o}^{\text{new}}$ with ${\overset{ˆ}{z}}_{j,o}^{\text{new}}={\overset{ˆ}{f}}_{j}\left(x_{j,o}^{\text{new}}\right)$.

For prediction, the posterior probability (2) is estimated with sample versions of (3) and (4), respectively. Let ${\overset{ˆ}{\Delta }}_{t}$ is the subvector of $\overset{ˆ}{\Delta }$ corresponding to $Z_{t}^{\text{new}}$. We generate an independent sample of $Z_{t}^{\text{new}}$, ${\left\{z_{t}^{\text{new}(s)}\right\}}_{s=1}^{S}$, conditional on $Z_{o}^{\text{new}}={\overset{ˆ}{z}}_{o}^{\text{new}}$ and $Z_{t}^{\text{new}}<{\overset{ˆ}{\Delta }}_{t}$ from the following multivariate truncated Gaussian distribution. Let $\text{var}\left(Z_{t}^{\text{new}}\right)=\Sigma_{t}$, $\text{var}\left(Z_{o}^{\text{new}}\right)=\Sigma_{o}$, and $\text{cov}\left(Z_{o}^{\text{new}},Z_{t}^{\text{new}}\right)=\Sigma_{ot}$. By properties of the multivariate Gaussian distribution, $\text{E}\left(Z_{t}|Z_{o}\right)=\Sigma_{ot}^{⊤}\Sigma_{o}^{-1}Z_{o}$ and $\text{var}\left(Z_{t}|Z_{o}\right)=\Sigma_{t}-\Sigma_{ot}^{⊤}\Sigma_{o}^{-1}\Sigma_{ot}$. By plugging estimators ${\overset{ˆ}{Z}}_{o}^{\text{new}},{\overset{ˆ}{\Sigma }}_{t},{\overset{ˆ}{\Sigma }}_{o},{\overset{ˆ}{\Sigma }}_{ot}$, and ${\overset{ˆ}{\Delta }}_{t}$, we obtain the multivariate truncated Gaussian distribution with the probability density

(9) $$p\left(z_{t}|{\overset{ˆ}{z}}_{o}^{\text{new}},z_{t}<{\overset{ˆ}{\Delta }}_{t}\right)=\frac{\phi_{p_{t}}\left(z_{t};\overset{ˆ}{\gamma },\overset{ˆ}{\Gamma }\right)}{\text{Pr}\left(Z_{t}<{\overset{ˆ}{\Delta }}_{t}|Z_{o}={\overset{ˆ}{z}}_{o}^{\text{new}}\right)}1\left(z_{t}<{\overset{ˆ}{\Delta }}_{t}\right),$$

where $\phi_{p}(z;\gamma ,\Gamma )$ denotes the p-dimensional Gaussian density with mean γ and covariance matrix Γ, $\overset{ˆ}{\gamma }={\overset{ˆ}{\Sigma }}_{ot}^{⊤}{\overset{ˆ}{\Sigma }}_{o}^{-1}{\overset{ˆ}{z}}_{o}^{\text{new}}$, and $\overset{ˆ}{\Gamma }={\overset{ˆ}{\Sigma }}_{t}-{\overset{ˆ}{\Sigma }}_{ot}^{⊤}{\overset{ˆ}{\Sigma }}_{o}^{-1}{\overset{ˆ}{\Sigma }}_{ot}$. Combining ${\left\{z_{t}^{\text{new}(s)}\right\}}_{s=1}^{S}$ with the estimates $\overset{ˆ}{\beta }$ from Section 2.4, $\overset{ˆ}{v}={\left(1-{\overset{ˆ}{\Sigma }}_{21}^{⊤}{\overset{ˆ}{\Sigma }}_{22}^{-1}{\overset{ˆ}{\Sigma }}_{21}\right)}^{1/2}$, and ${\overset{ˆ}{\Delta }}_{y}$, a sample plug-in version of the posterior probability (3) is given by

(10) $$\hat{\text{Pr}}\left(Y=1|X^{\text{new}}\right)=S^{-1}\sum_{s=1}^{S}\Phi \left(\frac{{\overset{ˆ}{\beta }}_{t}^{⊤}z_{t}^{\text{new}(s)}+{\overset{ˆ}{\beta }}_{o}^{⊤}{\overset{ˆ}{z}}_{o}^{\text{new}}-{\overset{ˆ}{\Delta }}_{y}}{\overset{ˆ}{v}}\right).$$

Similarly, using the Monte Carlo estimate ${\tilde{\mu }}_{t}=S^{-1}\sum_{s=1}^{S}z_{t}^{\text{new}(s)}$ for $\text{E}\left(Z_{t}|Z_{o}={\overset{ˆ}{z}}_{o}^{\text{new}},Z_{t}<\right.{\overset{ˆ}{\Delta }}_{t})$, we have a sample version of the posterior probability (4) as

(11) $$\hat{\text{Pr}}\left(Y=1|X^{\text{new}}\right)=\Phi \left(\frac{{\overset{ˆ}{\beta }}_{t}^{⊤}{\tilde{\mu }}_{t}+{\overset{ˆ}{\beta }}_{o}^{⊤}{\overset{ˆ}{z}}_{o}^{\text{new}}-{\overset{ˆ}{\Delta }}_{y}}{\overset{ˆ}{v}}\right).$$

The corresponding sample Bayes rule assigns a new observation $X^{\text{new}}$ to class 1 if the sample posterior probability is greater than 0.5 and to class 0, otherwise. While the expression (11) for the posterior probability depends on $\overset{ˆ}{v}$, the corresponding classification rule does not—similar to the standard linear discriminant analysis rule.

## 3. Theoretical Results

In this section, we demonstrate that the estimated classification direction $\overset{ˆ}{\beta }$ from (8) is a consistent estimator for $\beta^{*}$ under the following assumptions.

**Assumption 1 (Latent correlations)** *All the off-diagonal elements of* Σ *satisfy* $\left|\Sigma_{jk}\right|\leq 1-\varepsilon_{r}$ *for some constant* $\varepsilon_{r}\in (0,1)$.

**Assumption 2 (Thresholds)** *All the thresholds* $\Delta_{j}$ *satisfy* $\left|\Delta_{j}\right|\leq M$ *for some constant* M.

**Assumption 3 (Condition number)** $\text{C}(\Sigma )=\frac{\lambda_{\text{max}}(\Sigma )}{\lambda_{\text{min}}(\Sigma )}\leq C_{\text{cov}}$ *for some constant* $C_{\text{cov}}$.

**Assumption 4 (Sparsity)** $\beta^{*}$ *is sparse with the support* $𝒮=\left\{j:\beta_{j}^{*}\neq 0\right\}$ *with* s=card(𝒮).

**Assumption 5 (Sample size)** slogp=o(n).

Assumptions 1–2 are needed to guarantee consistency of estimated latent correlations in ${\overset{ˆ}{\Sigma }}_{22}$ and ${\overset{ˆ}{\Sigma }}_{21}$. Under these assumptions, the level of zero inflation, represented by the sizes of thresholds $\Delta_{j}$ and $\Delta_{k}$, affects the convergence rate at most by introducing a constant factor (Fan et al., 2017; Yoon et al., 2020). Assumptions 3–5 are used to account for the high-dimensional setting, where p is large, potentially much greater than n. We also take advantage of the restricted eigenvalue condition.

**Definition 5 (Restricted eigenvalue condition)** *A* p×p *matrix* Σ *satisfies the restricted eigenvalue condition* RE(s,3) *with parameter* γ=γ(Σ) *if for all sets* 𝒮⊂{1,…,p} *with* card(𝒮)≤s, *and for all* $a\in 𝒞(𝒮,3)=\left\{a\in R^{p}:{\left\|a_{{𝒮}^{c}}\right\|}_{1}\leq 3{\left\|a_{𝒮}\right\|}_{1}\right\}$, *it holds that*

$$a^{⊤}\Sigma a\geq \gamma^{-1}{\left\|a_{𝒮}\right\|}_{2}^{2}.$$

First, we provide the deterministic bound on estimation error, a standard bound for high-dimensional sparse regression (Bickel et al., 2009; Hastie et al., 2015; Negahban et al., 2012). For completeness, the proof is presented in Appendix C.

**Theorem 6** *Under Assumption 4, if* $\lambda \geq 2{\left\|{\overset{ˆ}{\Sigma }}_{21}-{\overset{ˆ}{\Sigma }}_{22}\beta^{*}\right\|}_{\infty }$ *and* ${\overset{ˆ}{\Sigma }}_{22}$ *satisfy* RE(s,3) *with parameter* γ, *then*

$${\left\|\overset{ˆ}{\beta }-\beta^{*}\right\|}_{2}\leq \frac{15}{2}\gamma \sqrt{s}\lambda .$$

To derive the probabilistic bound, we need to control the size of the tuning parameter, that is ${\left\|{\overset{ˆ}{\Sigma }}_{21}-{\overset{ˆ}{\Sigma }}_{22}\beta^{*}\right\|}_{\infty }$, and also ensure that the restricted eigenvalue condition on Σ implies the condition on $\overset{ˆ}{\Sigma }$. The existing results on the consistency of $\overset{ˆ}{\Sigma }$ (Yoon et al., 2020) provide the following high probability bounds: ${\left\|{\overset{ˆ}{\Sigma }}_{21}-\Sigma_{21}\right\|}_{\infty }\leq C\sqrt{\text{log}\;p/n}$ and ${\left\|{\overset{ˆ}{\Sigma }}_{22}-\Sigma_{22}\right\|}_{\infty }\leq C\sqrt{\text{log}\;p/n}$. A direct application of these results to control ${\left\|{\overset{ˆ}{\Sigma }}_{21}-{\overset{ˆ}{\Sigma }}_{22}\beta^{*}\right\|}_{\infty }$ gives

$${\left\|{\overset{ˆ}{\Sigma }}_{21}-{\overset{ˆ}{\Sigma }}_{22}\beta^{*}\right\|}_{\infty }\leq {\left\|{\overset{ˆ}{\Sigma }}_{21}-\Sigma_{21}\right\|}_{\infty }+{\left\|\left(\Sigma_{22}-{\overset{ˆ}{\Sigma }}_{22}\right)\beta^{*}\right\|}_{\infty }\;\leq C\sqrt{\text{log}\;p/n}+{\left\|{\overset{ˆ}{\Sigma }}_{22}-\Sigma_{22}\right\|}_{\infty }{\left\|\beta^{*}\right\|}_{1}\leq C_{1}\sqrt{\text{log}\;p/n}{\left\|\beta^{*}\right\|}_{1}.$$

The above bound is sub-optimal as ${\left\|\beta^{*}\right\|}_{1}$ scales approximately $s^{1/2}$, implying that the knowledge of true sparsity level is required to choose the tuning parameter λ. In contrast, the results from sparse high-dimensional regression (Bickel et al., 2009; Hastie et al., 2015; Negahban et al., 2012) suggest that the optimal rate should be of the order $\sqrt{\text{log}\;p/n}$ without the extra dependence on s. Our main theoretical contribution is obtaining the optimal bound for ${\left\|{\overset{ˆ}{\Sigma }}_{21}-{\overset{ˆ}{\Sigma }}_{22}\beta^{*}\right\|}_{\infty }$ under the model (1).

**Theorem 7** *Under Assumptions 1–5, for any* η∈(0,1), *there exists some constant* C>0 *such that*

$${\left\|{\overset{ˆ}{\Sigma }}_{21}-{\overset{ˆ}{\Sigma }}_{22}\beta^{*}\right\|}_{\infty }\leq C\sqrt{\frac{\text{log}\left(p\eta^{-1}\right)}{n}}$$

*with probability at least 1-η*.

The full proof is presented in Appendix C, and here we summarize the argument at a high level. To our knowledge, the only similar result is obtained by Barber and Kolar (2018) in the case of continuous Gaussian copula of Definition 1. Their proof, however, takes advantage of the closed form of the inverse bridge function $G^{-1}$ in the continuous case, which is a scaled cosine function. Due to the significantly higher complexity of the bridge function $G_{TT}$ in (7) for the truncated Gaussian copula case, its inverse lacks a closed-form expression, which makes the proof more challenging. An additional complication arises from substituting true thresholds $\Delta_{j}$ with their estimators ${\overset{ˆ}{\Delta }}_{j}$, these thresholds being unique to the truncated case. To overcome these challenges, we consider the 2nd-order Taylor expansion of ${\overset{ˆ}{\sigma }}_{jk}=G_{TT}^{-1}\left({\overset{ˆ}{\tau }}_{jk},{\overset{ˆ}{\Delta }}_{j},{\overset{ˆ}{\Delta }}_{k}\right)$ with respect to $\sigma_{jk}=G_{TT}^{-1}\left(\tau_{jk},\Delta_{j},\Delta_{k}\right)$. To control the first-order terms, we combine the bound on first derivatives of inverse bridge functions (Yoon et al., 2020) with the concentration bound for deviations of quadratic forms involving the Kendall’s τ correlation matrix (Barber and Kolar, 2018)[Lemma E.2] and sign sub-Gaussian property of the Gaussian vectors (Barber and Kolar, 2018)[Lemma 4.5]. To control the second-order terms, we show that the second derivatives of inverse bridge functions are bounded and use these bounds in conjunction with element-wise convergence of $\overset{ˆ}{\Sigma }$ and $\overset{ˆ}{\Delta }$. Due to the inverse bridge function not being available in a closed form, establishing bounds on the second derivative is highly non-trivial and is a major technical part of the proof. A similar technique proves that the restricted eigenvalue condition on Σ implies the condition holds for $\overset{ˆ}{\Sigma }$ (Lemma A.6 in Appendix C.3), leading to our final estimation bound.

**Theorem 8** *Under Assumptions 1–5, if* $\lambda =C\sqrt{\text{log}\;p/n}$ *for some constant* C>0 *and* $\Sigma_{22}$ *satisfies* RE(s,3) *with parameter* γ, *then*

$${\left\|\overset{ˆ}{\beta }-\beta^{*}\right\|}_{2}^{2}=O_{p}\left(\gamma^{2}\frac{s\;\text{log}\;p}{n}\right).$$

The obtained rate in estimation error coincides with the optimal rate in sparse linear regression (Bickel et al., 2009; Hastie et al., 2015; Negahban et al., 2012). The developed technique can be applied to establish estimation consistency within the general semiparametric Gaussian copula regression framework (Dey and Zipunnikov, 2022), encompassing all continuous, binary, ordinal, and truncated variable types.

## 4. Simulation

We empirically evaluate the performance of the proposed method, which we name SEmiparametric Discriminant Analysis (SEDA), with two approaches for estimating class-conditional probabilities as described in Section 2.5: formula (10) based on the Monte Carlo approximation and formula (11) based on the Taylor approximation, where we set the size of Monte Carlo samples S=100 from (9). Given a fixed tuning parameter λ, we solve (8) with a solver implemented in C in the R package MGSDA (Gaynanova, 2021). For comparison, we consider high-dimensional copula discriminant analysis (CODA) of Han et al. (2013), sparse semiparametric discriminant analysis (SSDA) of Mai and Zou (2015), negative binomial linear discriminant analysis (NBLDA) of Dong et al. (2016), classification and clustering of sequencing data using a Poisson model (PoiClaClu) of Witten (2011), random forest (RF) of Breiman (2001), sparse logistic regression (S-Logistic) of Friedman et al. (2010), and sparse support vector machine (S-SVM) of Yi and Huang (2017). Comprehensive implementation details are provided in Appendix B.1.

To generate synthetic data, we fix the number of covariates p=300 and consider three correlation structures for the latent Gaussian vector associated with the covariates:

1. Autoregressive (AR), $\Sigma_{22}={\left[0.7^{\left|j-j^{\prime }\right|}\right]}_{1\leq j,j^{\prime }\leq p}$.
2. Compound symmetry (CS), $\Sigma_{22}=0.7I_{p}+0.31_{pp}$; where $I_{p}$ and $1_{pp}$ are the p×p identity matrix and matrix of ones, respectively.
3. Geometric decaying eigenvalues (GD): $\Sigma_{22}=\Gamma N\Gamma^{⊤}$ where Γ is generated from the uniform distribution on p-dimensional orthogonal group (Chikuse, 2003, Theorem 2.2.1) and N is a diagonal matrix with geometrically decaying eigenvalues $\nu_{1}>\nu_{2}>\cdots >\nu_{p}$, where
$$\nu_{j}=\frac{p\left(0.9^{j-1}-0.9^{j}\right)}{\left(1-0.9^{p}\right)},\;j=1,\ldots ,p.$$

In the AR setting, each variable is strongly positively correlated with only a few variables, whereas in the CS setting, all variables are moderately positively correlated. In the GD setting, the correlation structure mimics the high-dimensional real data (Lee et al., 2013), with a wide range of correlations from −0.7 to 0.7.

We consider two model settings: joint model (as in Definition 3) and mixture model (proposed in Han et al. (2013), thus favoring CODA). For both models, we use the quantitative microbiome profiling data of Vandeputte et al. (2017) as a reference to mimic the real-world data. The true classification direction vector $\beta^{*}$ is set so that only the first s=0.05p=15 variables are non-zero. For the joint model, we consider three levels of zero-inflation (truncation): no truncation (0% zeros), low truncation (10%–50% zeros in each variable) and high truncation (50%–80% zeros in each variable). We use the empirical cumulative distribution functions of the corresponding reference variables to generate p=300 covariates (since the number of reference variables is fewer than 300, we recycle the empirical cumulative distribution functions to generate multiple synthetic variables).

For the mixture model, we exclusively consider the non-truncation case for a fair comparison with CODA. As a benchmark for classification accuracy, we define an Oracle rule for each model, using the Bayes plug-in rule that incorporates the true $\beta^{*}$ while estimating the underlying transformations as in (A1) and (A3). Comprehensive details regarding the data generation mechanism employed for each model and the oracle rules are contained in Appendix B.”

For each model and correlation structure, we consider equally (50:50) and unequally (20:80) proportioned class sizes and fix the sample sizes of training and test data at n=150 and $n_{\text{test}}=300$, respectively. We consider 100 replications for each population setting, correlation structure, and truncation level (for the joint setting).

To assess the prediction performance, we evaluate average misclassification rates on test data, which are reported in Table 1. For SEDA, we found that both approximations (10) and (11) of the conditional class probability led to practically the same misclassification rates, and thus, we only report the results for (11) (the results for (10) are in Appendix Table A1). When compared to parametric models in joint settings (NBLDA, PoiClaClu, S-Logistic, S-SVM), the proposed SEDA performs significantly better, especially with the increase in levels of zero inflation, confirming that existing models struggle to simultaneously address skewness and zero-inflation. Compared with semiparametric CODA, the proposed SEDA still performs significantly better, even under the population model settings favorable to CODA (i.e., the mixture setting without zero inflation). We suspect this is due to extreme skewness in simulated data, which affects the quality of observation level moment estimates that CODA relies on. SSDA, another semiparametric approach, has better performance than CODA and is comparable to SEDA in the mixture setting with no zero inflation, but is worse than SEDA in the joint setting as the proportion of zeros increases. When compared to the fully nonparametric random forest, SEDA has competitive performance in the equal class size case and is significantly better in the unequal class size case.

To assess the variable selection performance, we calculate the Matthews correlation coefficient (Matthews, 1975). A larger value represents a better variable selection performance, with the two boundary values, 1 and −1, indicating the completely correct and incorrect variable selections, respectively. Average MCC values across 100 replications for each method except random forest (which does not provide variable selection) are provided in Table A2. The proposed SEDA performs the best in almost all settings and is worse by the magnitude of at most 0.06 when it’s second best. Overall, the proposed SEDA obtains the best or second-best results in both classification accuracy and model selection performance and is the best overall performing method when considering both metrics.

## 5. Application to Sequencing Data

We assess the classification performance of SEDA and competing methods on three sequencing data sets: the Quantitative Microbiome Profiling (QMP) data of Vandeputte et al. (2017), microRNA data from breast cancer patients available through The Cancer Genome Atlas Project (Cancer Genome Atlas Network, 2012), and single-cell RNA (scRNA) sequencing data from the 10x Genomics website (https://www.10xgenomics.com).

The QMP data are not compositional but quantitative (Vandeputte et al., 2017), and we use the data set processed as in Yoon et al. (2019). The QMP data contain p=101 genera from $n_{0}=29$ patients with Crohn’s disease (Y=0) and $n_{1}=106$ healthy controls (Y=1). The proportions of zeros across all 101 genera range from 1% to 80%, with 14 genera having no zeros. As the data are heavily skewed, we also consider two popularly used transformations: log transformation after adding the pseudo count one (log-transformed) and modified centered log-ratio transformation (Yoon et al., 2019) (mclr-transformed). Unlike log transformation, mclr transformation is not monotone; thus, we expect the results of all methods, including the proposed SEDA, to change. The mclr transformation is designed for compositional rather than quantitative microbiome data (Yoon et al., 2019) and can be viewed as evaluating the robustness of the results to the total per-sample count. Our goal is to construct a classification rule that can separate patients with Crohn’s disease from healthy controls and to identify key genera that influence the rule.

The microRNA breast cancer data contain abundances of p=423 microRNAs from n=348 breast cancer patients, where $n_{0}=66$ patients have Basal-like tumor type (Y=0) and $n_{1}=282$ patients have other sub-types (Y=1). The proportions of zeros across all 423 variables range from 0.3% to 49%, with 206 variables having no zeros. As the data are skewed, we consider square-root and log transformations, where log transformation is applied after adding a pseudo-count of one. We aim to construct a classification rule that separates the Basal-like tumor type from other types.

The scRNA sequencing data contain p=329 genes of $n_{0}=77$ CXCR4-negative centrocyte B-cells and $n_{1}=188$ immature B-Cells from the lymphoblastoid cell line (LCL) GM12878. The proportions of zeros across 329 genes range from 2% to 90%, with 6 genes having no zeros. As well as the original data, we consider two transformations: log transformation after adding the pseudo count one (log-transformed) and square-root transformation.

For each data set, we apply the same methods as in Section 4, using 100 random splits into training (4/5) and testing (1/5). Table 2 displays the average misclassification rates and model sizes. Moreover, we evaluate the stability of variable selection across data sets with distinct transformations utilizing the Jaccard index (Jaccard, 1912). The Jaccard index compares the size of shared elements, $\text{card}\left({𝒮}_{1}\cap {𝒮}_{2}\right)$, to the total unique elements, $\text{card}\left({𝒮}_{1}\cup \right.{𝒮}_{2})$, between two estimated signal sets, ${𝒮}_{1}$ and ${𝒮}_{2}$, from distinct transformed data sets. The index’s scale ranges from zero (indicating an empty intersection) to one (representing complete agreement). For more than two sets, we employ the average pairwise Jaccard indices (Lang, 2022). For each fold, we compute the Jaccard index across transformed data sets and present the average values in Table 2.

Under the untransformed original data sets, the proposed method significantly outperforms all other methods having the lowest misclassification rates. Under the transformed data sets, the error rates of most other methods are notably improved. In contrast, the results of PoiClaClu, RF, SSDA, and the proposed method are not much affected by the transformations. In particular, the results of the proposed method and SSDA are not affected by the log and square root transformations at all, as the transformation preserves the rank of observed values. Specifically, the rank-based correlation estimator and the marginal transformations based on the empirical cumulative distribution function do not depend on the variables' scales but on the observations' rank. On the contrary, the mclr transformation changes the rank of the observations by applying observation-wise transformation. Hence, the error rate of the proposed method and SSDA on the mclr-transformed QMP data are slightly different from the original QMP data. However, SSDA shows overall inferior misclassification rates, particularly on QMP and scRNA data sets, likely because of the relatively higher zero-inflation compared to the microRNA data set. Although CODA employs the rank correlation and marginal transformation estimators as ours, its error rates differ substantially between original and transformed data sets. This sensitivity is possibly due to the moment matching constraints as the applied transformations dramatically change the first moments of the two classes, resulting in different CODA classifiers. Furthermore, CODA exhibits the lowest variable selection stability by selecting significantly different signal sets across various transformations.

On QMP data sets, PoiClaClu demonstrates relatively robust performance in class prediction and variable selection across diverse transformations. This robustness might stem from the inherent power transformation of data within the R package PoiClaClu, aimed at addressing overdispersion under the Poisson model (Witten, 2011). However, noticeable differences in variable selection between the original and log-transformed microRNA and scRNA data sets suggest sensitivity of model selection to potential misspecification.

NBLDA exhibits high variable selection stability, potentially attributed to its selection of a large number (or all) of variables. However, its classification error rates are larger than those of other methods and vary significantly across transformed data sets. The average misclassification rates and model sizes of the proposed method exhibit modest (mlcr-transformed) or no (log-transformed or square-root-transformed) changes, demonstrating its expected robustness.

Overall, the results from the three data sets consistently convey that: 1) the proposed SEDA is always the best-performing method on original highly-skewed and zero-inflated data, with a significant margin of error improvement; 2) the performance of other methods can be significantly improved with data transformations that mitigate skewness, but the resulting misclassification errors and selected variables are dependent on the transformation choice; 3) the proposed SEDA maintains competitive or better accuracy even when accounting for transformations while being consistent in selected variables, facilitating the robustness and reproducibility of analyses.

## 6. Discussion

There are several further research directions that could be pursued. First, our estimation consistency result is non-trivial, leading us to develop a new technique to facilitate underlying theoretical analyses by combining sub-Gaussian properties of sign vector with newly established bounds on second derivatives of inverse bridge function for truncated/truncated cases. The theoretical techniques introduced in this work can be extended beyond the specific application considered in the manuscript. While we have focused on the binary-truncated mixed model in Definition 3, the framework can flexibly accommodate the bridge functions of continuous, binary, ordinal, and truncated variables, ensuring estimation consistency in high-dimensional settings under a semiparametric latent Gaussian copula regression model, such as the model considered in Dey and Zipunnikov (2022). This generalization requires establishing bounds on the first and second derivatives of corresponding inverse bridge functions, which, while technical, can be accomplished similarly as in Appendix C.3. Secondly, our focus here is on the binary classification problem, and multi-class extensions are of interest. In case the classes have a natural ordering, e.g., a disease classification as “Mild”, “Moderate,” or “Severe,” the extension is straightforward by considering the ordinal-truncated mixed model, with corresponding bridge function for ordinal-truncated case as derived in Huang et al. (2021). However, it is unclear how to incorporate unordered class labels due to ambiguity in the underlying latent Gaussian representation, making it a compelling question for future study. The R implementing SEDA are available at https://github.com/heech31/SEDA.

## Supplementary Material

1

## Figures and Tables

**Table 1:** Average misclassification rates (%) for simulated data based on 100 replications, with standard errors in parentheses

Joint	Oracle	CODA	NBLDA	PoiClaClu	RF	S-Logistic	SSDA	S-SVM	SEDA_L_
(50:50)	No truncation								
AR	7.9 (0.2)	17.5 (0.3)	19.5 (0.3)	16.0 (0.3)	14.0 (0.2)	15.3 (0.2)	13.4 (0.3)	21.9 (0.8)	13.0 (0.3)
CS	7.6 (0.2)	17.2 (0.3)	22.7 (0.3)	20.4 (0.3)	13.7 (0.2)	16.2 (0.2)	15.7 (0.3)	15.0 (0.2)	14.8 (0.3)
GD	8.7 (0.2)	16.7 (0.3)	22.0 (0.4)	22.3 (0.4)	20.3 (0.3)	19.6 (0.4)	17.2 (0.3)	21.7 (0.4)	16.4 (0.3)
	Low truncation								
AR	8.8 (0.2)	23.9 (0.3)	31.1 (0.4)	19.8 (0.3)	14.9 (0.2)	23.4 (0.3)	17.0 (0.3)	33.8 (0.6)	15.0 (0.3)
CS	8.3 (0.2)	19.1 (0.3)	27.2 (0.3)	22.5 (0.5)	14.0 (0.2)	19.3 (0.2)	17.2 (0.3)	16.5 (0.3)	14.9 (0.3)
GD	8.3 (0.2)	22.9 (0.4)	24.7 (0.4)	22.7 (0.4)	20.5 (0.3)	25.9 (0.5)	19.9 (0.3)	30.5 (0.6)	17.0 (0.3)
	High truncation								
AR	11.0 (0.2)	36.1 (0.6)	33.5 (0.3)	17.5 (0.3)	16.7 (0.2)	28.8 (0.3)	20.1 (0.4)	37.5 (0.6)	18.5 (0.3)
CS	9.6 (0.2)	32.9 (0.6)	32.0 (0.4)	29.8 (0.5)	14.3 (0.2)	20.9 (0.2)	18.8 (0.3)	17.1 (0.3)	15.8 (0.2)
GD	8.6 (0.2)	29.4 (0.6)	25.1 (0.4)	23.0 (0.4)	21.0 (0.4)	30.3 (0.7)	22.6 (0.4)	32.7 (0.6)	18.2 (0.3)
(20:80)	No truncation								
AR	5.5 (0.1)	14.6 (0.2)	15.8 (0.3)	17.2 (0.3)	17.4 (0.2)	16.3 (0.3)	10.9 (0.2)	20.0 (0.2)	9.8 (0.2)
CS	5.3 (0.1)	11.9 (0.2)	16.1 (0.2)	16.5 (0.4)	11.3 (0.1)	13.0 (0.2)	12.8 (0.2)	10.2 (0.2)	11.0 (0.2)
GD	6.1 (0.1)	13.3 (0.2)	18.6 (0.3)	26.4 (0.5)	16.4 (0.2)	16.7 (0.3)	13.7 (0.2)	16.1 (0.2)	12.6 (0.2)
	Low truncation								
AR	6.8 (0.1)	22.0 (1.1)	19.1 (0.2)	19.3 (0.5)	18.8 (0.2)	19.9 (0.2)	14.3 (0.2)	20.2 (0.2)	12.3 (0.2)
CS	6.4 (0.1)	14.6 (0.2)	16.8 (0.2)	13.6 (0.4)	10.8 (0.1)	15.1 (0.3)	12.9 (0.2)	11.1 (0.2)	11.0 (0.2)
GD	6.0 (0.1)	16.7 (0.3)	17.3 (0.3)	27.5 (0.6)	16.8 (0.2)	19.2 (0.2)	14.9 (0.2)	20.1 (0.4)	12.9 (0.2)
	High truncation								
AR	9.5 (0.2)	21.2 (0.6)	19.9 (0.3)	23.2 (0.4)	19.7 (0.2)	19.8 (0.2)	20.3 (0.2)	20.0 (0.2)	16.7 (0.2)
CS	7.6 (0.1)	21.5 (0.9)	17.1 (0.2)	27.3 (1.2)	11.2 (0.2)	16.7 (0.3)	16.5 (0.2)	12.7 (0.3)	11.7 (0.2)
GD	6.5 (0.1)	21.4 (1.1)	17.0 (0.3)	26.7 (0.4)	17.3 (0.2)	19.6 (0.2)	17.7 (0.3)	20.1 (0.3)	13.6 (0.2)
Mixture	Oracle	CODA	NBLDA	PoiClaClu	RF	S-Logistic	SSDA	S-SVM	SEDA_L_
(50:50)	No truncation								
AR	10.0 (0.2)	15.3 (0.3)	41.4 (1.0)	41.5 (1.0)	11.7 (0.2)	11.6 (0.2)	12.5 (0.3)	12.8 (0.4)	12.2 (0.2)
CS	10.2 (0.2)	14.9 (0.3)	15.9 (0.2)	16.7 (0.3)	13.1 (0.2)	12.0 (0.2)	13.1 (0.3)	14.2 (0.2)	12.9 (0.2)
GD	9.8 (0.2)	13.9 (0.2)	21.6 (0.4)	22.0 (0.4)	13.5 (0.2)	12.4 (0.2)	13.1 (0.3)	14.3 (0.3)	13.2 (0.3)
(20:80)	No truncation								
AR	7.3 (0.1)	12.6 (0.3)	41.5 (0.4)	44.9 (0.5)	11.6 (0.2)	10.7 (0.2)	9.5 (0.2)	11.3 (0.2)	9.1 (0.2)
CS	7.3 (0.1)	11.7 (0.2)	15.3 (0.3)	15.9 (0.3)	9.6 (0.2)	11.2 (0.2)	10.7 (0.3)	9.9 (0.2)	9.8 (0.2)
GD	7.2 (0.2)	11.0 (0.2)	21.1 (0.4)	22.4 (0.5)	11.5 (0.2)	11.5 (0.3)	10.3 (0.2)	10.8 (0.2)	10.0 (0.2)

**Table 2:** Average misclassification rates (%), model sizes, and stability measured by the Jaccard indices (%) for real data sets based on 100 random splits, with standard errors in parentheses.

	CODA	NBLDA	PoiClaClu	RF	S-Logistic	SSDA	S-SVM	SEDA_L_
QMP (original)								
Error	10.25 (0.56)	12.96 (0.64)	4.32 (0.36)	5.79 (0.43)	14.00 (0.50)	7.57 (0.47)	10.46 (0.50)	2.93 (0.31)
Size	37.65 (1.22)	101 (0)	37.39 (2.18)	–	6.51 (0.31)	35.05 (1.77)	50.69 (1.27)	24.09 (1.51)
QMP (log-transformed)								
Error	2.18 (0.25)	3.79 (0.39)	3.64 (0.34)	5.75 (0.45)	3.93 (0.31)	7.57 (0.47)	6.79 (0.41)	2.93 (0.31)
Size	8.09 (0.43)	84.93 (2.22)	38.60 (1.54)	–	12.94 (0.37)	35.05 (1.77)	19.45 (2.12)	24.09 (1.51)
QMP (mclr-transformed)								
Error	2.75 (0.31)	3.54 (0.30)	3.57 (0.31)	4.32 (0.35)	2.21 (0.22)	3.61 (0.32)	4.36 (0.39)	2.79 (0.31)
Size	10.41 (1.12)	68.17 (2.70)	36.04 (2.32)	–	11.12 (0.33)	41.17 (2.40)	23.24 (1.36)	10.45 (0.95)
Stability	22.74 (0.70)	73.20 (1.51)	63.69 (1.44)	–	30.73 (0.53)	55.60 (1.10)	30.63 (1.52)	59.14 (1.47)
Breast cancer microRNA (original)								
Error	4.85 (0.23)	16.08 (0.49)	4.30 (0.24)	4.06 (0.19)	6.39 (0.26)	3.56 (0.20)	7.00 (0.25)	3.08 (0.20)
Size	52.92 (0.92)	423 (0)	385.48 (6.69)	–	20.51 (0.4)	70.03 (4.34)	44.05 (1.94)	86.34 (7.67)
Breast cancer microRNA (log-transformed)								
Error	3.17 (0.18)	5.97 (0.28)	5.97 (0.29)	3.93 (0.20)	3.58 (0.17)	3.56 (0.20)	3.23 (0.18)	3.08 (0.20)
Size	22.42 (3.28)	216.68 (20.78)	42.33 (7.37)	–	9.12 (0.34)	70.03 (4.34)	17.79 (1.37)	86.34 (7.67)
Breast cancer microRNA (square-root-transformed)								
Error	3.07 (0.19)	4.99 (0.24)	4.24 (0.24)	3.99 (0.20)	4.87 (0.21)	3.56 (0.20)	4.39 (0.21)	3.08 (0.20)
Size	50.74 (2.08)	419.06 (3.40)	387.27 (6.46)	–	13.78 (0.39)	70.03 (4.34)	31.29 (2.06)	86.34 (7.67)
Stability	20.41 (0.37)	67.22 (3.31)	39.59 (1.21)	–	40.55 (0.62)	100 (0)	31.57 (0.83)	100 (0)
B-cell scRNA (original)								
Error	7.29 (0.36)	10.71 (0.48)	6.77 (0.32)	4.21 (0.24)	5.73 (0.31)	6.13 (0.33)	12.02 (0.60)	3.50 (0.23)
Size	23.33 (0.74)	328.17 (0.32)	278.18 (4.29)	–	26.65 (0.33)	10.14 (0.23)	43.63 (1.89)	59.53 (4.21)
B-cell scRNA (log-transformed)								
Error	9.08 (0.34)	4.08 (0.22)	3.71 (0.22)	4.10 (0.24)	4.19 (0.25)	6.13 (0.33)	5.83 (0.29)	3.50 (0.23)
Size	10.41 (1.28)	149.42 (9.70)	164.89 (7.47)	–	26.72 (0.45)	10.14 (0.23)	40.23 (1.60)	59.53 (4.21)
B-cell scRNA (square-root-transformed)								
Error	7.65 (0.34)	5.88 (0.32)	5.94 (0.33)	4.08 (0.24)	4.63 (0.29)	6.13 (0.33)	6.12 (0.31)	3.50 (0.23)
Size	16.30 (0.55)	326.41 (1.82)	258.74 (5.36)	–	27.34 (0.41)	10.14 (0.23)	46.24 (1.97)	59.53 (4.21)
Stability	31.15 (4.32)	68.11 (8.27)	60.42 (4.58)	98.42 (0.24)	63.13 (2.80)	100 (0)	44.51 (4.00)	100 (0)
