# Supplementary material for: Sparse Semiparametric Discriminant Analysis for High-dimensional Zero-inflated Data
Source: J Mach Learn Res. Author manuscript; Available in PMC 2026 Jun 19. (PMC13278561)
Supplement: 1 [file NIHMS2181167-supplement-1.pdf]

Appendix A. First-order Taylor approximation of the posterior probability

Let $\mu_t = \mathrm{E}(Z_t \mid Z_o = z_o, Z_t \leq \Delta_t)$ and

$$g(Z_t) = \Phi\left(\frac{\beta_t^{*\top} Z_t + \beta_o^{*\top} z_o - \Delta_y}{v}\right).$$

Then, by Taylor expansion,

$$\begin{aligned}
\Pr(Y = 1 \mid X) &= \mathrm{E}\left\{g(Z_t) \mid Z_o = z_o, Z_t < \Delta_t\right\} \\
&\approx \mathrm{E}\left\{g(\mu_t) + \nabla g(\mu_t)^\top (Z_t - \mu_t) \mid Z_o = z_o, Z_t < \Delta_t\right\} \\
&= g(\mu_t) + \nabla g(\mu_t)^\top \mathrm{E}\left\{(Z_t - \mu_t) \mid Z_o = z_o, Z_t < \Delta_t\right\} \\
&= g(\mu_t).
\end{aligned}$$

Appendix B. Additional numerical results

B.1 Implementation details of the methods

We consider high-dimensional COpula Discriminant Analysis (CODA) of Han et al. (2013), Negative Binomial Linear Discriminant Analysis (NBLDA) of Dong et al. (2016), Classification and Clustering of Sequencing Data Based on a Poisson Model (PoiClaClu) of Witten (2011), Random Forest (RF) of Breiman (2001), Sparse Logistic regression (S-Logistic) of Friedman et al. (2010), Sparse Semiparametric Discriminant Analysis (SSDA) of Mai and Zou (2015), and Sparse Support Vector Machine (S-SVM) of Yi and Huang (2017) using `R` packages `NBLDA` (Goksuluk et al., 2022), `PoiClaclu` (Witten, 2019), `randomForest` (Liaw and Wiener, 2002), `glmnet` (Friedman et al., 2010), and `sparseSVM` (Yi and Zeng, 2018), respectively.

Since CODA, SSDA, and SEDA do not have available software, we use the default settings described in the original papers. For all methods, we use the default settings in the associated software for selecting tuning parameters and classification.

For both CODA and SSDA, the sparsity tuning parameter is chosen via 5-fold cross-validation to minimize the misclassification error rate. In both methods, the intercept in the classification rule is set with the optimal intercept of Mai et al. (2012). Specifically, given $\hat{\beta}$, whether from CODA or SSDA, the corresponding optimal intercept $\hat{\beta}_0^{\text{opt}}$ is

$$\hat{\beta}_0^{\text{opt}} = -\hat{\mu}_a^\top \hat{\beta} + \frac{\hat{\beta}^\top \hat{S} \hat{\beta}}{\hat{\mu}_d^\top \hat{\beta}} \log\left(\frac{n_1}{n_0}\right),$$

where $\hat{S}$, $\hat{\mu}_a$, and $\hat{\mu}_d$ are the estimated common covariance matrix, global mean, and mean difference, respectively. Using the optimal intercept, the sample classification rule assigns a new observation X^{new} to class 1 if

$$\{\hat{f}(X^{\text{new}}) - \hat{\mu}_a\}^\top \hat{\beta} + \hat{\beta}_0^{\text{opt}} > 0$$

and to class 0, otherwise, where $\hat{f} = (\hat{f}_1, \dots, \hat{f}_p)^\top$ denotes the estimated copula transformation. For SEDA, both the sparsity tuning parameter λ and the intercept Δ_y are selected based on 5-fold cross-validation to minimize the misclassification error rate using a grid of 100 values for each.

B.2 Joint model

We generate data from the latent Gaussian copula model for binary/truncated mixed data as in Definition 3. Recall that given full correlation matrix Σ, the population direction is $\beta^* = \Sigma_{22}^{-1}\Sigma_{21}$. To generate β^* with a given support $\mathcal{S} = \{j : \beta_j^* \neq 0\}$ for each of the three correlation structures Σ_{22} from above, we define Σ_{21} as follows.

Let $b = (b_1, \dots, b_p)^\top \in \{0,1\}^p$ be the indicator vector for the signal variables such that $b_j = 1$ if $j \in \mathcal{S}$ and $b_j = 0$, otherwise. Let $v^2 = 1 - \Sigma_{21}^\top \Sigma_{22}^{-1} \Sigma_{21} = 0.05$ be the prespecified conditional variance of $Z_y | Z_1, \dots, Z_p$. We set $\Sigma_{21} = \{(1 - v^2)/b^\top \Sigma_{22} b\}^{1/2} \Sigma_{22} b$ to ensure positive-definiteness of the full correlation matrix Σ with the desired sparsity of $\beta^* = \{(1 - v^2)/b^\top \Sigma_{22} b\}^{1/2} b$.

Given Σ, we follow the synthetic microbiome data generation mechanism proposed in Yoon et al. (2019). Specifically, we select monotone transformations and truncation levels so that the resulting synthetic X follows the empirical marginal cumulative distributions of the reference data of Vandeputte et al. (2017). To investigate the effect of truncation, we divide all 101 reference variables according to three truncation levels: no truncation (0%), low truncation (10%-50%), and high truncation (40%-80%). For each level, we use the empirical cumulative distribution functions of the corresponding reference variables to generate $p = 300$ covariates (as the number of the reference variables is less than 300, we use the same empirical cdf to generate multiple synthetic variables).

Let $\tilde{F}_j$ be the empirical cumulative distribution function chosen to represent variable X_j. For $i = 1, \dots, n$, we generate $(Z_{i,y}, Z_i)^\top \sim \mathrm{N}_{1+p}(0, \Sigma)$ and obtain Y_i and $X_i = (X_{i1}, \dots, X_{ip})^\top$ as $Y_i = 1(Z_{i,y} > \Delta_y)$, $X_{ij} = \tilde{F}_j^- \circ \Phi(Z_{ij})$, $j = 1, \dots, p$, where $\tilde{F}_j^-(u) = \min_i\{X_{ij} \mid \tilde{F}_j(X_{ij}) \geq u\}$. For the balanced and unbalanced class settings, we set $\Delta_y =$

$\Phi^{-1}(0.5) = 0$ and $\Delta_y = \Phi^{-1}(0.2) = -0.842$ resulting in $\Pr(Y = 0) = 0.5$ and $\Pr(Y = 0) = 0.2$, respectively. Marginally, this data generation scheme for X_j is the uniform sampling with replacement of the observations of the jth reference variable, but the joint association structure is induced by the prespecified latent correlation matrix Σ_{22}. Under the joint population, we define the Oracle classification rule as

$$\delta_J(X^{\text{new}}) = 1\left\{\beta_t^\top \tilde{\mu}_t + \beta_o^\top \hat{f}_o(X_o^{\text{new}}) - \Delta_y > 0\right\}, \tag{A1}$$

where the marginal transformation for the observed variables $\hat{f}_o$ is estimated with the training sample, $\tilde{\mu}_t$ is estimated with $\hat{Z}_o^{\text{new}}$ and the true latent correlation matrix (11), β_t and β_o are from the true latent correlation matrix Σ, and Δ_y is the population threshold.

B.3 Mixture model

Han et al. (2013) consider the following model:

$$X|(Y = g) \sim \text{NPN}(\mu_g, \Sigma, f) \quad (g = 0, 1), \tag{A2}$$

where $\mu_g \in \mathbb{R}^p$ is the mean of class $g = 0, 1$ and $\Sigma \in \mathbb{R}^{p\times p}$ is a common covariance matrix. Thus, unlike Definition (1), CODA allows latent Gaussian vector to have a non-zero mean and covariance matrix by restricting monotone transformations, f_j ($j = 1, \ldots, p$), to be mean and variance preserving, i.e., each f_j satisfies the following moment matching conditions:

$$\begin{aligned} \text{E}\{f_j(X_j)|Y = g\} &= \text{E}(X_j|Y = g) = \mu_{j,g} \quad (j = 1, \ldots, p), \\ \text{var}\{f_j(X_j)|Y = g\} &= \text{var}(X_j|Y = g) = \sigma_j^2 \quad (j = 1, \ldots, p). \end{aligned}$$

This model does not account for zero inflation; it assumes continuous X.

To generate realistic simulation data, we set $\Sigma = S\Sigma_{22}S$, where $S = \text{diag}(s_1, \ldots, s_p)$ contains the sample standard deviations of the reference variables and Σ_{22} is one of the three correlation structures described in Section 4. We set the class means μ_g ($g = 0, 1$) and discriminant direction β^* as the following.

Let $\mu_a = (\mu_1 + \mu_0)/2$ and $\mu_d = \mu_1 - \mu_0$ be the global mean and mean difference, respectively. Under model (A2), the Bayes classification direction is given by $\beta^* = \Sigma^{-1}\mu_d$. When $\Pr(Y = 1) = \Pr(Y = 0)$, the Bayes error rate is $\alpha = \Phi\left(-q^{1/2}/2\right)$, where $q = \beta^{*\top}\Sigma\beta^*$. Given the support $\mathcal{S}$, let $b \in \{0, 1\}^p$ be the corresponding indicator vector such that $b_j = 1$ if $j \in \mathcal{S}$ and $b_j = 0$, otherwise. Fixing the Bayes error rate at $\alpha = 0.1$, we generate β^* as $\beta^* = -2\Phi^{-1}(\alpha)b/(b^\top\Sigma b)^{1/2}$, and obtain $\mu_d = \Sigma\beta^*$. Finally, we set $\mu_0 = C1_p$ and $\mu_1 = \mu_0 + \mu_d$, where the constant $C > 0$ is chosen sufficiently large to mimic the means of the reference data, leading to generated synthetic data with non-negative values.

Given μ_g and Σ from above, we generate $Z_i|(Y_i = g) \sim \text{N}(\mu_g, \Sigma)$ ($i = 1, \ldots, n;\ g = 0, 1$), where class sizes are $n_0 = n_1 = n/2 = 75$ for the balanced setting, and $n_0 = 30$ and $n_1 = 120$ for the unbalanced setting. To obtain continuous $X_1^*, \ldots, X_n^*$ that follow model (A2), we use the identity transformation (Han et al., 2013; Liu et al., 2009), i.e., $Z_j = X_j^* = X_j$, $j = 1, \ldots, p$.

Under the mixture model, the Oracle classification rule is

$$\delta_M(X) = 1\left\{ \left(\hat{f}(X) - \mu_a\right)^\top \beta^* + \log \frac{\Pr(Y=1)}{\Pr(Y=0)} > 0 \right\}, \tag{A3}$$

where $\hat{f} = (\hat{f}_1, \ldots, \hat{f}_p)^\top$ is estimated from the training data as in Section 3.2. of Han et al. (2013), μ_a is the true population global mean, $\beta^* = \Sigma^{-1}\mu_d$, and $\Pr(Y = g)$ is the population proportion of the class g.

B.4 Additional results on simulated data

This section provides complete simulation results. Table A1 display average misclassification rates including SEDA with (10), and Table A2 displays the average Matthews correlation coefficient (Matthews, 1975), where a larger value represents a better variable selection performance. The two boundary values, 1 and -1, indicate the completely correct and incorrect variable selections, respectively. Random forest is omitted, as it does not provide variable selection. The two approximations, Taylor and Monte Carlo, of the conditional class probability results in practically the same misclassification rates and variable selections.

Table A1: Average missclassification rates (%) for simulated data based on 100 replications, with standard errors in parentheses.

Joint	CODA	NBLDA	PoiClaClu	RF	S-Logistic	SSDA	S-SVM	SEDA_L	SEDA_MC
(50:50)				No truncation					
AR	17.5 (0.3)	19.5 (0.3)	16.0 (0.3)	14.0 (0.2)	15.3 (0.2)	13.4 (0.3)	21.9 (0.8)	13.0 (0.3)	13.0 (0.3)
CS	17.2 (0.3)	22.7 (0.3)	20.4 (0.3)	13.7 (0.2)	16.2 (0.2)	15.7 (0.3)	15.0 (0.2)	14.8 (0.3)	14.8 (0.3)
GD	16.7 (0.3)	22.0 (0.4)	22.3 (0.4)	20.3 (0.3)	19.6 (0.4)	17.2 (0.3)	21.7 (0.4)	16.4 (0.3)	16.4 (0.3)
				Low truncation					
AR	23.9 (0.3)	31.1 (0.4)	19.8 (0.3)	14.9 (0.2)	23.4 (0.3)	17.0 (0.3)	33.8 (0.6)	15.0 (0.3)	15.0 (0.3)
CS	19.1 (0.3)	27.2 (0.3)	22.5 (0.5)	14.0 (0.2)	19.3 (0.2)	17.2 (0.3)	16.5 (0.3)	14.9 (0.3)	14.9 (0.3)
GD	22.9 (0.4)	24.7 (0.4)	22.7 (0.4)	20.5 (0.3)	25.9 (0.5)	19.9 (0.3)	30.5 (0.6)	17.0 (0.3)	17.0 (0.3)
				High truncation					
AR	36.1 (0.6)	33.5 (0.3)	17.5 (0.3)	16.7 (0.2)	28.8 (0.3)	20.1 (0.4)	37.5 (0.6)	18.5 (0.3)	18.6 (0.3)
CS	32.9 (0.6)	32 (0.4)	29.8 (0.5)	14.3 (0.2)	20.9 (0.2)	18.8 (0.3)	17.1 (0.3)	15.8 (0.2)	15.8 (0.2)
GD	29.4 (0.6)	25.1 (0.4)	23 (0.4)	21.0 (0.4)	30.3 (0.7)	22.6 (0.4)	32.7 (0.6)	18.2 (0.3)	18.2 (0.3)
(20:80)				No truncation					
AR	14.6 (0.2)	15.8 (0.3)	17.2 (0.3)	17.4 (0.2)	16.3 (0.3)	10.9 (0.2)	20.0 (0.2)	9.8 (0.2)	9.8 (0.2)
CS	11.9 (0.2)	16.1 (0.2)	16.5 (0.4)	11.3 (0.1)	13.0 (0.2)	12.8 (0.2)	10.2 (0.2)	11.0 (0.2)	11.0 (0.2)
GD	13.3 (0.2)	18.6 (0.3)	26.4 (0.5)	16.4 (0.2)	16.7 (0.3)	13.7 (0.2)	16.1 (0.2)	12.6 (0.2)	12.6 (0.2)
				Low truncation					
AR	22.0 (1.1)	19.1 (0.2)	19.3 (0.5)	18.8 (0.2)	19.9 (0.2)	14.3 (0.2)	20.2 (0.2)	12.3 (0.2)	12.3 (0.2)
CS	14.6 (0.2)	16.8 (0.2)	13.6 (0.4)	10.8 (0.1)	15.1 (0.3)	12.9 (0.2)	11.1 (0.2)	11.0 (0.2)	11.0 (0.2)
GD	16.7 (0.3)	17.3 (0.3)	27.5 (0.6)	16.8 (0.2)	19.2 (0.2)	14.9 (0.2)	20.1 (0.4)	12.9 (0.2)	12.9 (0.2)
				High truncation					
AR	21.2 (0.6)	19.9 (0.3)	23.2 (0.4)	19.7 (0.2)	19.8 (0.2)	20.3 (0.2)	20.0 (0.2)	16.7 (0.2)	16.7 (0.2)
CS	21.5 (0.9)	17.1 (0.2)	27.3 (1.2)	11.2 (0.2)	16.7 (0.3)	16.5 (0.2)	12.7 (0.3)	11.7 (0.2)	11.7 (0.2)
GD	21.4 (1.1)	17.0 (0.3)	26.7 (0.4)	17.3 (0.2)	19.6 (0.2)	17.7 (0.3)	20.1 (0.3)	13.6 (0.2)	13.5 (0.2)

Mixture	CODA	NB-DA	PoiClaClu	RF	S-Logistic	SSDA	S-SVM	SEDA_L	SEDA_MC
(50:50)				No truncation					
AR	15.3 (0.3)	41.4 (1.0)	41.5 (1.0)	11.7 (0.2)	11.6 (0.2)	12.5 (0.3)	12.8 (0.4)	12.2 (0.2)	12.2 (0.2)
CS	14.9 (0.3)	15.9 (0.2)	16.7 (0.3)	13.1 (0.2)	12.0 (0.2)	13.1 (0.3)	14.2 (0.2)	12.9 (0.2)	12.9 (0.2)
GD	13.9 (0.2)	21.6 (0.4)	22.0 (0.4)	13.5 (0.2)	12.4 (0.2)	13.1 (0.3)	14.3 (0.3)	13.2 (0.3)	13.2 (0.3)
(20:80)				No truncation					
AR	12.6 (0.3)	41.5 (0.4)	44.9 (0.5)	11.6 (0.2)	10.7 (0.2)	9.5 (0.2)	11.3 (0.2)	9.1 (0.2)	9.1 (0.2)
CS	11.7 (0.2)	15.3 (0.3)	15.9 (0.3)	9.6 (0.2)	11.2 (0.2)	10.7 (0.3)	9.9 (0.2)	9.8 (0.2)	9.8 (0.2)
GD	11.0 (0.2)	21.1 (0.4)	22.4 (0.5)	11.5 (0.2)	11.5 (0.3)	10.3 (0.2)	10.8 (0.2)	10.0 (0.2)	10.0 (0.2)

Table A2: Average Matthews correlation coefficients and standard errors over 100 replications from the joint and mixture populations.

Joint	CODA	NBLDA	PoiClaClu	S-Logistic	SSDA	S-SVM	$SEDA_L$	$SEDA_{MC}$
(50:50)				No truncation				
AR	0.20 (0.01)	0.00 (0.00)	0.62 (0.02)	0.65 (0.01)	0.70 (0.01)	0.54 (0.02)	0.72 (0.01)	0.72 (0.01)
CS	0.21 (0.01)	0.00 (0.00)	0.00 (0.01)	0.22 (0.01)	0.23 (0.01)	0.03 (0.00)	0.20 (0.01)	0.20 (0.01)
GD	0.14 (0.00)	0.00 (0.00)	0.09 (0.01)	0.16 (0.01)	0.23 (0.01)	0.11 (0.01)	0.18 (0.01)	0.17 (0.01)
				Low truncation				
AR	0.11 (0.00)	0.00 (0.00)	0.55 (0.02)	0.48 (0.01)	0.67 (0.01)	0.43 (0.01)	0.69 (0.02)	0.69 (0.02)
CS	0.12 (0.00)	0.00 (0.00)	0.05 (0.01)	0.10 (0.01)	0.20 (0.01)	0.00 (0.00)	0.18 (0.01)	0.18 (0.01)
GD	0.07 (0.01)	0.00 (0.00)	0.08 (0.01)	0.08 (0.01)	0.17 (0.01)	0.08 (0.01)	0.15 (0.01)	0.15 (0.01)
				High truncation				
AR	0.15 (0.01)	0.00 (0.00)	0.63 (0.02)	0.45 (0.01)	0.62 (0.01)	0.38 (0.01)	0.65 (0.01)	0.65 (0.01)
CS	0.06 (0.01)	0.00 (0.00)	0.02 (0.01)	0.10 (0.01)	0.15 (0.01)	0.02 (0.00)	0.16 (0.01)	0.16 (0.01)
GD	0.09 (0.00)	0.00 (0.00)	0.09 (0.01)	0.09 (0.01)	0.14 (0.01)	0.09 (0.01)	0.13 (0.01)	0.13 (0.01)
(20:80)				No truncation				
AR	0.20 (0.01)	0.00 (0.00)	0.55 (0.02)	0.51 (0.01)	0.58 (0.01)	0.19 (0.02)	0.66 (0.01)	0.66 (0.01)
CS	0.20 (0.01)	0.00 (0.00)	0.02 (0.00)	0.15 (0.01)	0.16 (0.01)	0.03 (0.00)	0.15 (0.01)	0.15 (0.01)
GD	0.11 (0.01)	0.00 (0.00)	0.08 (0.01)	0.05 (0.01)	0.17 (0.01)	0.04 (0.01)	0.15 (0.01)	0.15 (0.01)
				Low truncation				
AR	0.04 (0.00)	0.00 (0.00)	0.47 (0.03)	0.19 (0.02)	0.50 (0.01)	0.06 (0.01)	0.62 (0.01)	0.62 (0.01)
CS	0.10 (0.00)	0.00 (0.00)	0.03 (0.00)	0.07 (0.01)	0.12 (0.01)	-0.02 (0.00)	0.13 (0.01)	0.13 (0.01)
GD	0.05 (0.00)	0.00 (0.00)	0.08 (0.01)	-0.01 (0.00)	0.06 (0.01)	-0.01 (0.00)	0.12 (0.01)	0.12 (0.01)
				High truncation				
AR	0.07 (0.01)	0.00 (0.00)	0.58 (0.02)	0.02 (0.01)	0.35 (0.01)	0.04 (0.01)	0.55 (0.01)	0.55 (0.01)
CS	0.04 (0.01)	0.00 (0.00)	0.00 (0.00)	0.05 (0.01)	0.06 (0.01)	0.01 (0.00)	0.08 (0.01)	0.08 (0.01)
GD	0.04 (0.01)	0.00 (0.00)	0.08 (0.01)	-0.01 (0.00)	0.03 (0.01)	-0.01 (0.00)	0.11 (0.01)	0.11 (0.01)

Mixture	CODA	NB-LDA	PoiClaClu	S-Logistic	SSDA	S-SVM	$SEDA_L$	$SEDA_{MC}$
(50:50)				No truncation				
AR	0.11 (0.00)	0.13 (0.01)	0.14 (0.01)	0.60 (0.01)	0.54 (0.02)	0.71 (0.02)	0.65 (0.01)	0.65 (0.01)
CS	0.03 (0.00)	-0.02 (0.00)	-0.03 (0.00)	0.22 (0.01)	0.20 (0.01)	0.03 (0.00)	0.22 (0.01)	0.22 (0.01)
GD	0.03 (0.00)	0.00 (0.00)	0.01 (0.00)	0.21 (0.01)	0.20 (0.01)	0.13 (0.01)	0.19 (0.01)	0.19 (0.01)
(20:80)				No truncation				
AR	0.09 (0)	0.02 (0.01)	0.09 (0.01)	0.58 (0.01)	0.49 (0.01)	0.36 (0.01)	0.61 (0.01)	0.61 (0.01)
CS	0.00 (0)	-0.01 (0.00)	0.00 (0.00)	0.20 (0.01)	0.17 (0.01)	0.08 (0.00)	0.16 (0.01)	0.16 (0.01)
GD	0.03 (0)	0.00 (0.00)	0.00 (0.00)	0.21 (0.01)	0.19 (0.01)	0.09 (0.01)	0.17 (0.01)	0.17 (0.01)

Appendix C. Proofs of theoretical results

C.1 Notation and assumptions

In this section, we prove the results stated in the main manuscript. For completeness, we start with restating the notations and assumptions from the main manuscript as follows. For a vector $a \in \mathbb{R}^p$, we denote the ℓ_q-norm, $q \in [0, \infty)$, by $\|a\|_q = (\sum_{j=1}^p |a_j|^q)^{1/q}$ and the ℓ_∞-norm by $\|a\|_\infty = \max_{1\le j\le p} |a_j|$. For two vectors with the same size, $a, b, c \in \mathbb{R}^p$, we write $a < b$ to denote element-wise inequalities such that $a_j < b_j$, $j = 1, \dots, p$ and $a \in (b, c)$ whether $b < a < c$ or $c < a < b$. The vectors $1_p, 0_p \in \mathbb{R}^p$ denote the one and zero vectors and matrices $I_p, 1_{pp} \in \mathbb{R}^{p\times p}$ denote the identity and matrix with ones. For a matrix $A \in \mathbb{R}^{n\times p}$, $\|A\|_\infty = \max_{j,k} |a_{jk}|$ denotes its ℓ_∞-norm, and for a square matrix $T \in \mathbb{R}^{p\times p}$, $|T|$ denotes its determinant, and $\lambda_{\max}(T)$ and $\lambda_{\min}(T)$ denote the largest and smallest eigenvalues of T. For two matrices with the same size, $A, B \in \mathbb{R}^{n\times p}$, $A \circ B$ denotes

the Hadamard product defined as $A \circ B = [a_{jk}b_{jk}] \in \mathbb{R}^{n \times p}$. For two functions f and g, we denote their composite function by $f \circ g = f(g(x))$. We let $1(\cdot)$ denote the indicator function taking the value 1 when its argument is true and 0 otherwise. For a sequence of random variables, $X_1, \ldots, X_n, \ldots$, we write $X_n = O_p(a_n)$ if, for any $\varepsilon \in (0,1)$, there exist $M, N > 0$ such that $\Pr(|X_n/a_n| > M) < \varepsilon$ for all $n > N$. We let $\Phi_d(a_1, \ldots, a_d; \Sigma)$ and $\Phi(\cdot)$ denote the d-dimensional Gaussian distribution function with zero mean and correlation matrix Σ evaluated at $(a_1, \ldots, a_d)^\top$ and the univariate standard Gaussian distribution function, respectively. We use C and $C_i, i = 1, 2, \ldots$, to denote generic constants that do not depend on the sample size n, dimension p, and model parameters, where their values may change from line to line. We write $\mathrm{card}(\mathcal{S})$ to denote the cardinality of a set $\mathcal{S}$.

Throughout, we use G to denote the bridge function such that for TT case $\tau_{jk} = G(\Sigma_{jk}, \Delta_j, \Delta_k)$ with $\Sigma_{22} = \left[G^{-1}(\tau_{jk}, \Delta_j, \Delta_k)\right]_{1 \le j,k \le p} = G^{-1}(T, \Delta)$ and $\hat{\Sigma}_{22} = G^{-1}(\hat{T}, \hat{\Delta})$. Here T and $\hat{T}$ are the population and sample Kendall's τ matrices, respectively, $\Delta = (\Delta_1, \ldots, \Delta_p)^\top$, $\hat{\Delta} = (\hat{\Delta}_1, \ldots, \hat{\Delta}_p)^\top$ with $\Delta_j = \Phi^{-1}(\pi_j)$ with $\pi_j = \Pr(Z_j \le \Delta_j) = \Pr(X_j = 0)$, and $\hat{\Delta}_j = \Phi^{-1}(\hat{\pi}_j)$ with $\hat{\pi}_j = \sum_{i=1}^n 1(X_{ij} = 0)/n$ being the sample zero proportion of the jth variable.

C.2 Proofs of the theorems from the main manuscript

Proof of Theorem 6 The proof follows the proof of Theorem 2 in Gaynanova (2020). For completeness, we provide the full proof as follows. By the optimality condition of equation (9) in the main manuscript, we have

$$\hat{\Sigma}_{22}\hat{\beta} - \hat{\Sigma}_{21} + \lambda g = 0,$$

where g is a subgradient of $\|\beta\|_1$ at $\hat{\beta}$. This gives

$$(\hat{\beta} - \beta^*)^\top(\hat{\Sigma}_{22}\hat{\beta} - \hat{\Sigma}_{21} + \lambda g) = 0,$$

and thus

$$(\hat{\beta} - \beta^*)^\top\hat{\Sigma}_{22}(\hat{\beta} - \beta^*) - (\hat{\beta} - \beta^*)^\top(\hat{\Sigma}_{21} - \hat{\Sigma}_{22}\beta^*) + \lambda(\hat{\beta} - \beta^*)^\top g = 0. \tag{A4}$$

Since g is a subgradient of $\|\beta\|_1$ at $\hat{\beta}$,

$$(\hat{\beta} - \beta^*)^\top g \ge \|\hat{\beta}\|_1 - \|\beta^*\|_1. \tag{A5}$$

By combining (A4), (A5), and Hölder's and triangle inequalities, we have

$$\begin{aligned}(\hat{\beta} - \beta^*)^\top\hat{\Sigma}_{22}(\hat{\beta} - \beta^*) &\le (\hat{\beta} - \beta^*)^\top(\hat{\Sigma}_{21} - \hat{\Sigma}_{22}\beta^*) + \lambda\|\beta^*\|_1 - \lambda\|\hat{\beta}\|_1 \\ &\le \|\hat{\beta} - \beta^*\|_1\|\hat{\Sigma}_{21} - \hat{\Sigma}_{22}\beta^*\|_\infty + \lambda\|\beta^*\|_1 - \lambda\|\hat{\beta}\|_1.\end{aligned}$$

Using the condition on λ and Assumption 4,

$$\begin{aligned}(\hat{\beta} - \beta^*)^\top\hat{\Sigma}_{22}(\hat{\beta} - \beta^*) &\le \frac{\lambda}{2}\|\hat{\beta} - \beta^*\|_1 + \lambda\|\beta^*\|_1 - \lambda\|\hat{\beta}\|_1 \\ &= \frac{\lambda}{2}\|\hat{\beta}_{\mathcal{S}} - \beta^*_{\mathcal{S}}\|_1 + \frac{\lambda}{2}\|\hat{\beta}_{\mathcal{S}^c}\|_1 + \lambda\|\beta^*\|_1 - \lambda\|\hat{\beta}\|_1 \\ &= \frac{\lambda}{2}\|\hat{\beta}_{\mathcal{S}} - \beta^*_{\mathcal{S}}\|_1 + \frac{\lambda}{2}\|\hat{\beta}_{\mathcal{S}^c}\|_1 + \lambda\|\beta^*_{\mathcal{S}}\|_1 - \lambda\|\hat{\beta}\|_1 \\ &= \frac{\lambda}{2}\|\hat{\beta}_{\mathcal{S}} - \beta^*_{\mathcal{S}}\|_1 + \frac{\lambda}{2}\|\hat{\beta}_{\mathcal{S}^c}\|_1 + \lambda\|\beta^*_{\mathcal{S}}\|_1 - \lambda\|\hat{\beta}_{\mathcal{S}}\|_1 - \lambda\|\hat{\beta}_{\mathcal{S}^c}\|_1.\end{aligned}$$

Using the triangle inequality,

$$(\hat{\beta} - \beta^*)^\top \hat{\Sigma}_{22}(\hat{\beta} - \beta^*) \le \frac{\lambda}{2}\|\hat{\beta}_{\mathcal{S}} - \beta^*_{\mathcal{S}}\|_1 + \frac{\lambda}{2}\|\hat{\beta}_{\mathcal{S}^c}\|_1 + \lambda\|\hat{\beta}_{\mathcal{S}} - \beta^*_{\mathcal{S}}\|_1 - \lambda\|\hat{\beta}_{\mathcal{S}^c}\|_1 \quad \text{(A6)}$$

$$= \frac{3\lambda}{2}\|\hat{\beta}_{\mathcal{S}} - \beta^*_{\mathcal{S}}\|_1 - \frac{\lambda}{2}\|\hat{\beta}_{\mathcal{S}^c}\|_1 \quad \text{(A7)}$$

$$\le \frac{3\lambda}{2}\|\hat{\beta}_{\mathcal{S}} - \beta^*_{\mathcal{S}}\|_1. \quad \text{(A8)}$$

As $(\hat{\beta} - \beta^*)^\top \hat{\Sigma}_{22}(\hat{\beta} - \beta^*)$ is non-negative and $\beta^*_{\mathcal{S}^c} = 0$, (A7) implies that $\|\hat{\beta}_{\mathcal{S}^c}\|_1 \le 3\|\hat{\beta}_{\mathcal{S}} - \beta^*_{\mathcal{S}}\|_1$, and thus, $\hat{\beta} - \beta^*$ is in the cone $\mathcal{C}(\mathcal{S}, 3)$. Since $\hat{\Sigma}_{22}$ satisfies RE$(s, 3)$ with paramter γ, we have

$$\|\hat{\beta}_{\mathcal{S}} - \beta^*_{\mathcal{S}}\|_2 \le \{\gamma(\hat{\beta} - \beta^*)^\top \hat{\Sigma}_{22}(\hat{\beta} - \beta^*)\}^{1/2}. \quad \text{(A9)}$$

Since $\|\hat{\beta}_{\mathcal{S}} - \beta^*_{\mathcal{S}}\|_1 \le s^{1/2}\|\hat{\beta}_{\mathcal{S}} - \beta^*_{\mathcal{S}}\|_2$, (A8) and (A9) imply that

$$(\hat{\beta} - \beta^*)^\top \hat{\Sigma}_{22}(\hat{\beta} - \beta^*) \le \frac{9}{4}\gamma s \lambda^2 \quad \text{(A10)}$$

The bound for $\|\hat{\beta}_{\mathcal{S}} - \beta^*_{\mathcal{S}}\|_2$ can be obtained as follows. Since $\hat{\beta} - \beta^* \in \mathcal{C}(\mathcal{S}, 3)$,

$$\|\hat{\beta} - \beta^*\|_1 = \|\hat{\beta}_{\mathcal{S}} - \beta^*_{\mathcal{S}}\|_1 + \|\hat{\beta}_{\mathcal{S}^c} - \beta^*_{\mathcal{S}^c}\|_1 \quad \text{(A11)}$$

$$\le 4\|\hat{\beta}_{\mathcal{S}} - \beta^*_{\mathcal{S}}\|_1 \quad \text{(A12)}$$

$$\le 4s^{1/2}\|\hat{\beta}_{\mathcal{S}} - \beta^*_{\mathcal{S}}\|_2 \quad \text{(A13)}$$

$$\le 4\{s\gamma(\hat{\beta} - \beta^*)^\top \hat{\Sigma}_{22}(\hat{\beta} - \beta^*)\}^{1/2} \quad \text{(A14)}$$

$$\le 4(s\gamma)^{1/2}\frac{3}{2}(\gamma s \lambda^2)^{1/2} = 6s\gamma\lambda. \quad \text{(A15)}$$

Let $a = \hat{\beta} - \beta^*$ for notational simplicity. For $j = 0, 1, \ldots, J$, let T_j be the index set of $(j+1)$th s largest (in absolute) elements of a. Then, $a \in \mathcal{C}(T_0, 3)$ as

$$\begin{aligned}
\|a_{T_0^c}\|_1 = \|a\|_1 - \|a_{T_0}\|_1 &\le \|a\|_1 - \|a_{\mathcal{S}}\|_1 \\
&= \|a_{\mathcal{S}^c}\|_1 \\
&\le 3\|a_{\mathcal{S}}\|_1 \quad \text{since } a \in \mathcal{C}(\mathcal{S}, 3) \\
&\le 3\|a_{T_0}\|_1.
\end{aligned}$$

Furthermore, it follows that $\|a_{T_j}\|_0 = s$ for $j = 0, \ldots, J-1$ with last $\|a_{T_J}\|_0 \le s$. Also, for $j \ge 1$, $\|a_{T_j}\|_2 \le s^{1/2}\|a_{T_j}\|_\infty \le s^{1/2}s^{-1}\|a_{T_{j-1}}\|_1$. Thus, by the triangle inequality,

$$\|a\|_2 \le \|a_{T_0}\|_2 + \sum_{j=1}^{J}\|a_{T_j}\|_2 \le \|a_{T_0}\|_2 + \sum_{j=1}^{J} s^{1/2}\|a_{T_j}\|_\infty \quad \text{(A16)}$$

$$\le \|a_{T_0}\|_2 + \sum_{j=0}^{J-1} s^{1/2}s^{-1}\|a_{T_j}\|_1 \le \|a_{T_0}\|_2 + s^{-1/2}\|a\|_1. \quad \text{(A17)}$$

Using that $\hat{\Sigma}_{22}$ satisfies RE$(s, 3)$ and $a \in \mathcal{C}(T_0, 3)$,

$$\begin{aligned}\|\hat{\beta} - \beta^*\|_2 &= \|a\|_2 \le \|a_{T_0}\|_2 + s^{-1/2}\|a\|_1 \\ &\le \{\gamma(\hat{\beta} - \beta^*)^\top \hat{\Sigma}_{22}(\hat{\beta} - \beta^*)\}^{1/2} + 6s^{1/2}\gamma\lambda \quad \text{as (A9) and (A15)} \\ &\le \frac{3}{2}\gamma s^{1/2}\lambda + 6s^{1/2}\gamma\lambda \quad \text{by (A10)} \\ &= \frac{15}{2}\gamma s^{1/2}\lambda.\end{aligned}$$

■

Proof of Theorem 7 Using $\beta^* = \Sigma_{22}^{-1}\Sigma_{21}$ and triangle inequality, we have

$$\begin{aligned}\|\hat{\Sigma}_{21} - \hat{\Sigma}_{22}\beta^*\|_\infty &= \|\hat{\Sigma}_{21} - \hat{\Sigma}_{22}\beta^* + \Sigma_{21} - \Sigma_{21}\|_\infty \\ &= \|\hat{\Sigma}_{21} - \Sigma_{21} + \Sigma_{21} - \hat{\Sigma}_{22}\beta^*\|_\infty \\ &\le \|\hat{\Sigma}_{21} - \Sigma_{21}\|_\infty + \|(\Sigma_{22} - \hat{\Sigma}_{22})\beta^*\|_\infty.\end{aligned}$$

For $\|\hat{\Sigma}_{21} - \Sigma_{21}\|_\infty$, it follows from Theorem 7 of Yoon et al. (2020) that

$$\|\hat{\Sigma}_{21} - \Sigma_{21}\|_\infty \le C_1\sqrt{\frac{\log(p\eta^{-1})}{n}} \tag{A18}$$

with probability at least $1 - \eta$.

Consider $\|(\Sigma_{22} - \hat{\Sigma}_{22})\beta^*\|_\infty$. Recall that $\Sigma_{22} = G^{-1}(T, \Delta) = \left[G^{-1}(\tau_{jk}, \Delta_j, \Delta_k)\right]_{1\le j,k\le p}$, and $\hat{\Sigma}_{22} = G^{-1}(\hat{T}, \hat{\Delta})$. Let $G_\tau^{-1} = \partial G^{-1}(\tau, \Delta_j, \Delta_k)/\partial\tau$ be the partial derivative of the inverse bridge function with respect to τ. By adding and subtracting $G^{-1}(\hat{T}, \Delta)$ from $G^{-1}(\hat{T}, \hat{\Delta})$ and applying the mean value theorem to $G^{-1}(\hat{T}, \Delta)$ with respect to $\hat{T}$,

$$\begin{aligned}\hat{\Sigma}_{22} = G^{-1}(\hat{T}, \hat{\Delta}) &= G^{-1}(\hat{T}, \Delta) + \{G^{-1}(\hat{T}, \hat{\Delta}) - G^{-1}(\hat{T}, \Delta)\} \\ &= G^{-1}(T, \Delta) + G_\tau^{-1}(\tilde{T}, \Delta) \circ (\hat{T} - T) + \{G^{-1}(\hat{T}, \hat{\Delta}) - G^{-1}(\hat{T}, \Delta)\} \\ &= \Sigma_{22} + G_\tau^{-1}(\tilde{T}, \Delta) \circ (\hat{T} - T) + \{G^{-1}(\hat{T}, \hat{\Delta}) - G^{-1}(\hat{T}, \Delta)\},\end{aligned}$$

where $\tilde{T} = [\tilde{\tau}_{jk}]_{1\le j,k\le p}$ and $\tilde{\tau}_{jk} \in (\hat{\tau}_{jk}, \tau_{jk})$.

Therefore

$$(\Sigma_{22} - \hat{\Sigma}_{22})\beta^* = -G_\tau^{-1}(\tilde{T}, \Delta) \circ (\hat{T} - T)\beta^* - \{G^{-1}(\hat{T}, \hat{\Delta}) - G^{-1}(\hat{T}, \Delta)\}\beta^*.$$

By letting

$$G_\tau^{-1}(\tilde{T}, \Delta) = G_\tau^{-1}(T, \Delta) + \{G_\tau^{-1}(\tilde{T}, \Delta) - G_\tau^{-1}(T, \Delta)\},$$

we further have, by the triangle inequality, that

$$\begin{aligned}\|(\Sigma_{22} - \hat{\Sigma}_{22})\beta^*\|_\infty \le\ & \underbrace{\|G_\tau^{-1}(T, \Delta) \circ (\hat{T} - T)\beta^*\|_\infty}_{:=I_1} + \underbrace{\|\{G_\tau^{-1}(\tilde{T}, \Delta) - G_\tau^{-1}(T, \Delta)\} \circ (\hat{T} - T)\beta^*\|_\infty}_{:=I_2} \\ & + \underbrace{\|\{G^{-1}(\hat{T}, \hat{\Delta}) - G^{-1}(\hat{T}, \Delta)\}\beta^*\|_\infty}_{:=I_3}.\end{aligned}$$

We separately bound I_1, I_2, and I_3 in Lemmas A.1, A.2, and A.3, respectively. Combining these bounds with (A18) completes the proof. ■

Proof of Theorem 8 From Theorem 6, if $\hat{\Sigma}_{22}$ satisfies $RE(s,3)$ and $\lambda \geq 2\|\hat{\Sigma}_{21} - \hat{\Sigma}_{22}\beta^*\|_\infty$, then

$$\|\hat{\beta} - \beta^*\|_2^2 \leq C_1\gamma^2 s\lambda^2.$$

From Theorem 7, if $\lambda = C\sqrt{\log p/n}$, then $\lambda \geq 2\|\hat{\Sigma}_{21} - \hat{\Sigma}_{22}\beta\|_\infty$ holds with high probability. From Lemma A.6, if Σ_{22} satisfies $RE(s,3)$ with parameter $\gamma(\Sigma_{22})$, then with high probability so does $\hat{\Sigma}_{22}$ with $\gamma(\hat{\Sigma}_{22}) = C_2\gamma(\Sigma_{22})$. Combining these results gives that, with high probability,

$$\|\hat{\beta} - \beta^*\|_2^2 \leq C_3\gamma^2 s\frac{\log p}{n},$$

leading to the desired bound. ■

C.3 Main supporting lemmas

Lemma A.1 *Under Assumptions 1—5, for any fixed $\eta \in (0,1)$, there exists some constant $C > 0$ such that*

$$\|G_\tau^{-1}(T,\Delta) \circ (\hat{T} - T)\beta^*\|_\infty \leq C\sqrt{\frac{\log(p\eta^{-1})}{n}}$$

with probability at least $1 - \eta$.

Proof Let $e_j \in \mathbb{R}^p$ be the vector with 1 in the jth component and 0 otherwise. Then

$$\begin{aligned}\|G_\tau^{-1}(T,\Delta) \circ (\hat{T} - T)\beta^*\|_\infty &= \max_{1\leq j\leq p} |e_j^\top G_\tau^{-1}(T,\Delta) \circ (\hat{T} - T)\beta^*| \\ &= \max_{1\leq j\leq p} \|m_j\|_2 |u^\top(\hat{T} - T)e_j|.\end{aligned}$$

where $m_j = (G_\tau^{-1}(\tau_{1j},\Delta_1,\Delta_j)\beta_1^*, \ldots, G_\tau^{-1}(\tau_{pj},\Delta_p,\Delta_j)\beta_p^*)^\top$ and $u = m_j/\|m_j\|_2$ is a deterministic unit vector. Since $|G_\tau^{-1}| \leq C_0$ for some constant $C_0 > 0$ by Theorem 6 of Yoon et al. (2020), we have $\|m_j\|_2 \leq C_0\|\beta^*\|_2 \leq C_0 C_{\text{cov}}$ by Lemma A.4. Therefore,

$$\|G_\tau^{-1}(T,\Delta) \circ (\hat{T} - T)\beta^*\|_\infty \leq C_0 C_{\text{cov}} \max_{1\leq j\leq p} |u^\top(\hat{T} - T)e_j|.$$

Consider $u^\top(\hat{T} - T)e_j$. By Lemma A.8, for any $\epsilon > 0$ and $0 < t \leq n/C_{\text{cov}}$,

$$\begin{aligned}\Pr\left(\max_{1\leq j\leq p} |u^\top(\hat{T} - T)e_j| \geq \epsilon\right) &\leq p \cdot \Pr\left(|u^\top(\hat{T} - T)e_1| \geq \epsilon\right) \\ &\leq 2p \cdot \Pr\left\{\exp\left(tu^\top(\hat{T} - T)e_1\right) \geq \exp(t\epsilon)\right\} \\ &\leq 2p \cdot \mathrm{E}\left[\exp\left(tu^\top(\hat{T} - T)e_1\right)\right]\exp(-t\epsilon) \\ &\leq 2p \cdot \exp\left(\frac{t^2 C_{\text{cov}}^2}{n} - t\epsilon\right) \\ &= 2\exp\left(\log p + \frac{t^2 C_{\text{cov}}^2}{n} - t\epsilon\right).\end{aligned}$$

Letting $\epsilon = 2C_{\mathrm{cov}}\sqrt{\log(2p\eta^{-1})/n}$ and $t = C_{\mathrm{cov}}^{-1}\sqrt{n\log(2p\eta^{-1})}$, we have

$$\Pr\left\{\max_{1\le j\le p}|u^\top(\hat{T}-T)e_j| \ge 2C_{\mathrm{cov}}\sqrt{\frac{\log(2p\eta^{-1})}{n}}\right\} \le 1-\eta.$$

Thus, for some constant $C>0$, $\|G_\tau^{-1}(T,\Delta)\circ(\hat{T}-T)\beta^*\|_\infty \le C\sqrt{\log(p\eta^{-1})/n}$ with probability at least $1-\eta$. ∎

Lemma A.2 *Under Assumptions 1—5, for any fixed $\eta\in(0,1)$, there exists some constant $C>0$ such that*

$$\left\|\left\{G_\tau^{-1}(\tilde{T},\Delta) - G_\tau^{-1}(T,\Delta)\right\}\circ(\hat{T}-T)\beta^*\right\|_\infty \le C\sqrt{\frac{\log(p\eta^{-1})}{n}}$$

with probability at least $1-\eta$.

Proof By the mean value theorem, for some $\bar{T} = [\bar{\tau}_{jk}]_{1\le j,k\le p}$, where $\bar{\tau}_{jk}\in(\tilde{\tau}_{jk},\tau_{jk})$, we have

$$\|\{G_\tau^{-1}(\tilde{T},\Delta) - G_\tau^{-1}(T,\Delta)\}\circ(\hat{T}-T)\beta^*\|_\infty = \|G_{\tau\tau}^{-1}(\bar{T},\Delta)\circ(\tilde{T}-T)\circ(\hat{T}-T)\beta^*\|_\infty,$$

where $G_{\tau\tau}^{-1}$ is the 2nd partial derivative of inverse bridge function with respect to τ. By Lemma A.11, $|G_{\tau\tau}^{-1}|\le C_0$ for some constant $C_0>0$. Since $|\bar{\tau}_{jk}-\tau_{jk}|\le|\tilde{\tau}_{jk}-\tau_{jk}|\le|\hat{\tau}_{jk}-\tau_{jk}|$, by Hölder's inequality,

$$\begin{aligned}\|\{G_\tau^{-1}(\tilde{T},\Delta) - G_\tau^{-1}(T,\Delta)\}\circ(\hat{T}-T)\beta^*\|_\infty &\le \|G_{\tau\tau}^{-1}(\bar{T},\Delta)\circ(\tilde{T}-T)\circ(\hat{T}-T)\|_\infty\|\beta^*\|_1\\ &\le C_0\|\tilde{T}-T\|_\infty\|\hat{T}-T\|_\infty\|\beta^*\|_1\\ &\le C_0\|\hat{T}-T\|_\infty^2\|\beta^*\|_1.\end{aligned}$$

By Lemma A.9, $\|\hat{T}-T\|_\infty^2 \le C_1\log(p\eta^{-1})/n$ with probability at least $1-\eta$, and by Lemma A.4, $\|\beta^*\|_1\le\sqrt{s}C_{\mathrm{cov}}$. Thus, under Assumption 5, for sufficiently large n and some constant $C>0$,

$$\|I_2\|_\infty \le C\frac{\sqrt{s}\log(p\eta^{-1})}{n} \le C\frac{\log(p\eta^{-1})}{n}$$

with probability at least $1-\eta$. ∎

Lemma A.3 *Under Assumptions 1—5, for any fixed $\eta\in(0,1)$, there exists some constant $C>0$ such that*

$$\left\|\left\{G^{-1}(\hat{T},\hat{\Delta}) - G^{-1}(\hat{T},\Delta)\right\}\beta^*\right\|_\infty \le C\sqrt{\frac{\log(p\eta^{-1})}{n}}$$

with probability at least $1-\eta$.

Proof We reparameterize $\Delta_j = \Phi^{-1}(\pi_j)$ and write $G^{-1}(T, \Delta) = G^{-1}(T, \pi)$, where $\pi = (\pi_1, \ldots, \pi_p)^\top$. We also write $G^{-1}_{\pi_1} = \partial G^{-1}(\tau, \pi_1, \pi_2)/\partial \pi_1$ and $G^{-1}_{\pi_2} = \partial G^{-1}(\tau, \pi_1, \pi_2)/\partial \pi_2$. For each element of $G^{-1}(\hat{T}, \hat{\pi}) - G^{-1}(\hat{T}, \pi)$, the multivariate mean value theorem gives

$$G^{-1}(\hat{\tau}_{jk}, \hat{\pi}_j, \hat{\pi}_k) - G^{-1}(\hat{\tau}_{jk}, \pi_j, \pi_k) = \underbrace{G^{-1}_{\pi_1}(\hat{\tau}_{jk}, \tilde{\pi}_j, \tilde{\pi}_k)(\hat{\pi}_j - \pi_j)}_{:=I_{3,1}} + \underbrace{G^{-1}_{\pi_2}(\hat{\tau}_{jk}, \tilde{\pi}_j, \tilde{\pi}_k)(\hat{\pi}_k - \pi_k)}_{:=I_{3,2}},$$

for some $\tilde{\pi}_j \in (\hat{\pi}_j, \pi_j)$ and $\tilde{\pi}_k \in (\hat{\pi}_k, \pi_k)$, respectively.

Consider $I_{3,1}$. By adding and subtracting $G^{-1}_{\pi_1}(\tau_{jk}, \pi_j, \pi_k)(\hat{\pi}_j - \pi_j)$ to $I_{3,1}$, we have that

$$\begin{aligned} &G^{-1}_{\pi_1}(\hat{\tau}_{jk}, \tilde{\pi}_j, \tilde{\pi}_k)(\hat{\pi}_j - \pi_j) \\ &\quad = \left\{G^{-1}_{\pi_1}(\hat{\tau}_{jk}, \tilde{\pi}_j, \tilde{\pi}_k) - G^{-1}_{\pi_1}(\tau_{jk}, \pi_j, \pi_k) + G^{-1}_{\pi_1}(\tau_{jk}, \pi_j, \pi_k)\right\}(\hat{\pi}_j - \pi_j) \\ &\quad = \left\{G^{-1}_{\pi_1}(\hat{\tau}_{jk}, \tilde{\pi}_j, \tilde{\pi}_k) - G^{-1}_{\pi_1}(\tau_{jk}, \pi_j, \pi_k)\right\}(\hat{\pi}_j - \pi_j) \\ &\qquad + G^{-1}_{\pi_1}(\tau_{jk}, \pi_j, \pi_k)(\hat{\pi}_j - \pi_j). \end{aligned}$$

By applying the multivariate mean value theorem to $\{G^{-1}_{\pi_1}(\hat{\tau}_{jk}, \tilde{\pi}_j, \tilde{\pi}_k) - G^{-1}_{\pi_1}(\tau_{jk}, \pi_j, \pi_k)\}$, we further have that

$$\begin{aligned} &G^{-1}_{\pi_1}(\hat{\tau}_{jk}, \tilde{\pi}_j, \tilde{\pi}_k)(\hat{\pi}_j - \pi_j) \\ &\quad = G^{-1}_{\pi_1\tau}(\bar{\tau}_{jk}, \bar{\pi}_j, \bar{\pi}_k)(\hat{\tau} - \tau)(\hat{\pi}_j - \pi_j) + G^{-1}_{\pi_1\pi_1}(\bar{\tau}_{jk}, \bar{\pi}_j, \bar{\pi}_k)(\tilde{\pi}_j - \pi_j)(\hat{\pi}_j - \pi_j) \\ &\qquad + G^{-1}_{\pi_1\pi_2}(\bar{\tau}_{jk}, \bar{\pi}_j, \bar{\pi}_k)(\tilde{\pi}_k - \pi_k)(\hat{\pi}_j - \pi_j) + G^{-1}_{\pi_1}(\tau_{jk}, \pi_j, \pi_k)(\hat{\pi}_j - \pi_j) \end{aligned}$$

for some $\bar{\tau}_{jk} \in (\hat{\tau}_{jk}, \tau_{jk})$, $\bar{\pi}_j \in (\tilde{\pi}_j, \pi_j)$, and $\bar{\pi}_k \in (\tilde{\pi}_k, \pi_k)$. Thus, by the triangle inequality,

$$\begin{aligned} \|G^{-1}_{\pi_1}(\hat{T}, \tilde{\pi}) \circ (\hat{\Pi} - \Pi)\beta^*\|_\infty \le &\|G^{-1}_{\pi_1\tau}(\bar{T}, \bar{\pi}) \circ (\hat{T} - T) \circ (\hat{\Pi} - \Pi)\beta^*\|_\infty && \text{(A19)} \\ &+ \|G^{-1}_{\pi_1\pi_1}(\bar{T}, \bar{\pi}) \circ (\tilde{\Pi} - \Pi) \circ (\hat{\Pi} - \Pi)\beta^*\|_\infty && \text{(A20)} \\ &+ \|G^{-1}_{\pi_1\pi_2}(\bar{T}, \bar{\pi}) \circ (\hat{\Pi}^\top - \Pi^\top) \circ (\hat{\Pi} - \Pi)\beta^*\|_\infty && \text{(A21)} \\ &+ \|G^{-1}_{\pi_1}(T, \pi) \circ (\hat{\Pi} - \Pi)\beta^*\|_\infty, && \text{(A22)} \end{aligned}$$

where $\Pi = \pi 1_p^\top$ and $\hat{\Pi} = \hat{\pi} 1_p^\top$. We consider (A19)—(A21) and (A22) separately as follows.

For (A19)—(A21), we know that $|G^{-1}_{\pi_1\tau}|$, $|G^{-1}_{\pi_1\pi_1}|$, and $|G^{-1}_{\pi_1\pi_2}|$ are bounded above by some positive constants by Lemmas A.14, A.12, A.13, respectively. Also, for some constants $C_0, C_1 > 0$ and a fixed $\eta \in (0, 1)$, $\|\hat{\pi} - \pi\|_\infty \le C_0\sqrt{\log(p\eta^{-1})/n}$ and $\|\hat{T} - T\|_\infty \le C_1\sqrt{\log(p\eta^{-1})/n}$ with probability at least $1 - \eta$ by Lemmas A.5 and A.9. Since $|\tilde{\pi}_j - \pi_j| \le |\hat{\pi}_j - \pi_j|$, it follows that $\|\tilde{\Pi} - \Pi\|_\infty \le \|\hat{\Pi} - \Pi\|_\infty$ and

$$\|\hat{\Pi} - \Pi\|_\infty = \|\hat{\Pi}^\top - \Pi^\top\|_\infty = \|\hat{\pi} - \pi\|_\infty.$$

Hence, we can show that, for some constant $C > 0$, (A19)—(A21) are bounded above by $C\sqrt{\log(p\eta^{-1})/n}$ with probability at least $1 - \eta$ by following the steps of Lemma A.2.

For (A22), we know that $|G^{-1}_{\pi_1}|$ is bounded above by some positive constant by Lemma A.10. Thus, by following the steps of Lemma A.1 with $(\hat{T} - T)$ being replaced by $(\hat{\Pi} - \Pi)$ and using Lemma A.5, it follows that, for some constant $C > 0$, (A22) is bounded above by

$C\sqrt{\log(p\eta^{-1})/n}$ with probability at least $1-\eta$. By combining these results, we have that, for some constant $C > 0$,

$$\|G_{\pi_1}^{-1}(\hat{T}, \tilde{\pi}) \circ (\hat{\Pi} - \Pi)\beta^*\|_\infty \le C\sqrt{\frac{\log(p\eta^{-1})}{n}} \tag{A23}$$

with probability at least $1-\eta$.

By symmetrically applying above steps to $I_{3,2}$, we also have that, for some constant $C > 0$,

$$\|G_{\pi_2}^{-1}(\hat{T}, \tilde{\pi}) \circ (\hat{\Pi} - \Pi)^\top\beta^*\|_\infty \le C\sqrt{\frac{\log(p\eta^{-1})}{n}} \tag{A24}$$

with probability at least $1-\eta$. Combining (A23) and (A24) completes the proof. ■

Lemma A.4 *Let $\beta^* = \Sigma_{22}^{-1}\Sigma_{21}$. Under Assumptions 3—4*

$$\|\beta^*\|_2 < C_{\text{cov}} \quad \textit{and} \quad \|\beta^*\|_1 < \sqrt{s}C_{\text{cov}}.$$

Proof Under Assumption 3

$$\|\beta^*\|_2 = \|\Sigma_{22}^{-1}\Sigma_{21}\|_2 \le \|\Sigma_{22}^{-1}\|_{\text{op}}\|\Sigma_{21}\|_2.$$

At the same time, using $e_1 = (1, 0, \dots, 0)^\top$,

$$\lambda_{\max}(\Sigma) \ge \|\Sigma e_1\|_2 = \sqrt{1 + \|\Sigma_{21}\|_2^2} > \|\Sigma_{21}\|_2.$$

Since $\|\Sigma_{22}^{-1}\|_{\text{op}} = \{\lambda_{\min}(\Sigma)\}^{-1}$, we have that

$$\|\beta^*\|_2 \le \|\Sigma_{22}^{-1}\|_{\text{op}}\|\Sigma_{21}\|_2 < \frac{\lambda_{\max}(\Sigma)}{\lambda_{\min}(\Sigma)} \le C_{\text{cov}}.$$

Under Assumption 4, it follows

$$\|\beta^*\|_1 \le \sqrt{s}\|\beta^*\|_2 < \sqrt{s}C_{\text{cov}}.$$

■

Lemma A.5 *For $j = 1, \dots, p$, let $\pi_j = \Phi(\Delta_j)$ and $\hat{\pi}_j = n^{-1}\sum_{i=1}^n 1(X_{ij} = 0)$, where $\mathrm{E}(\hat{\pi}_j) = \pi_j$. Also, let $\pi = (\pi_1, \dots, \pi_p)^\top$ and $\hat{\pi} = (\hat{\pi}_1, \dots, \hat{\pi}_p)^\top$. Then for any deterministic $\|u\|_2 = 1$ and $t \ge 0$, and some constant $C > 0$*

$$\mathrm{E}\left[\exp\{tu^\top(\hat{\pi} - \pi)\}\right] \le \exp\left(\frac{t^2C}{n}\right).$$

Proof For $i = 1, \ldots, n$, let $b_i = (1(X_{i1} = 0), \ldots, 1(X_{ip} = 0))^\top$ such that $n^{-1} \sum_{i=1}^n b_i = \hat{\pi}$. By definition of the truncated latent Gaussian copula model, we have

$$\begin{aligned} b_{ij} &= 1(X_{ij} = 0) = 1(X_{ij}^* \le D_j) = 1(Z_{ij} \le \Delta_j) \\ &= 1(Z_{ij} - \Delta_j \le 0) = \frac{\text{sign}(Z_{ij} - \Delta_j) + 1}{2}. \end{aligned}$$

Since $\tilde{Z}_i = Z_i - \Delta \overset{iid}{\sim} \text{N}_p(-\Delta, \Sigma_{22})$, where $Z_i = (Z_{i1}, \ldots, Z_{ip})^\top$ and $\Delta = (\Delta_1, \ldots, \Delta_p)^\top$, $\text{sign}(\tilde{Z}_i) - \text{E}\{\text{sign}(\tilde{Z}_i)\}$ is $\mathsf{C}(\Sigma_{22})$-subgaussian by Lemma A.7, and thus $\hat{\pi} - \pi = n^{-1} \sum_{i=1}^n \{b_i - \text{E}(b_i)\}$ is sum of n iid $\mathsf{C}(\Sigma_{22})$-subgaussians. Thus,

$$\begin{aligned} \text{E}\left\{\exp(tu^\top(\hat{\pi} - \pi))\right\} &= \text{E}\left[\exp\left\{\frac{t}{n}\sum_{i=1}^n u^\top(b_i - \text{E}(b_i))\right\}\right] \\ &\le \prod_{i=1}^n \exp\left(\frac{t^2 C}{n^2}\right) = \exp\left(\frac{t^2 C}{n}\right). \end{aligned}$$

■

Lemma A.6 *Let Σ_{22} satisfy RE$(s, 3)$ with parameter $\gamma = \gamma(\Sigma_{22})$. Let Assumptions 1, 2 and 5 hold. Then with probability $1 - O(p^{-1})$, $\hat{\Sigma}_{22}$ satisfies RE$(s, 3)$ with*

$$\hat{\gamma} = \gamma(\hat{\Sigma}_{22}) \le C\gamma$$

for some constant $C > 1$.

Proof Let $a \in \mathcal{C}(S, 3) = \{a \in \mathbb{R}^p : \|a_{S^c}\|_1 \le 3\|a_S\|_1\}$. Let T_0 be the index set of the s largest (in absolute) elements of a. Then it holds that $a \in \mathcal{C}(T_0, 3)$, and

$$\|a\|_1 = \|a_S\|_1 + \|a_S^c\|_1 \le 4\|a_S\|_1 \le 4s^{1/2}\|a_S\|_2 \le 4s^{1/2}\|a_{T_0}\|_2. \tag{A25}$$

Furthermore, following (A17), it holds that

$$\|a\|_2 \le \|a_{T_0}\|_2 + s^{-1/2}\|a\|_1 \le 5\|a_{T_0}\|_2. \tag{A26}$$

Consider

$$a^\top\hat{\Sigma}_{22}a = a^\top\Sigma_{22}a + a^\top(\hat{\Sigma}_{22} - \Sigma_{22})a \ge a^\top\Sigma_{22}a - |a^\top(\hat{\Sigma}_{22} - \Sigma_{22})a|. \tag{A27}$$

Following the proof of Theorem 7 and reparameterization of Δ in terms of $\Pi = \pi 1_p^\top$, we have the following decomposition

$$\begin{aligned}
\hat{\Sigma}_{22} - \Sigma_{22} &= G_\tau^{-1}(T, \Delta) \circ (\hat{T} - T) + G_{\tau\tau}^{-1}(\bar{T}, \Delta) \circ (\tilde{T} - T) \circ (\hat{T} - T) \\
&\quad + \{G^{-1}(\hat{T}, \hat{\Delta}) - G^{-1}(\hat{T}, \Delta)\} \\
&= G_\tau^{-1}(T, \Delta) \circ (\hat{T} - T) + G_{\tau\tau}^{-1}(\bar{T}, \Delta) \circ (\tilde{T} - T) \circ (\hat{T} - T) \\
&\quad + \{G^{-1}(\hat{T}, \hat{\Pi}) - G^{-1}(\hat{T}, \Pi)\} \\
&= G_\tau^{-1}(T, \Delta) \circ (\hat{T} - T) + G_{\tau\tau}^{-1}(\bar{T}, \Delta) \circ (\tilde{T} - T) \circ (\hat{T} - T) \\
&\quad + G_{\pi_1}^{-1}(\hat{T}, \tilde{\pi}) \circ (\hat{\Pi} - \Pi) + G_{\pi_2}^{-1}(\hat{T}, \tilde{\pi}) \circ (\hat{\Pi} - \Pi)^\top \\
&= G_\tau^{-1}(T, \Delta) \circ (\hat{T} - T) + G_{\pi_1}^{-1}(T, \pi) \circ (\hat{\Pi} - \Pi) + G_{\pi_2}^{-1}(T, \pi) \circ (\hat{\Pi} - \Pi)^\top \\
&\quad + G_{\tau\tau}^{-1}(\bar{T}, \Delta) \circ (\tilde{T} - T) \circ (\hat{T} - T) \\
&\quad + G_{\pi_1\tau}^{-1}(\bar{T}, \bar{\pi}) \circ (\hat{T} - T) \circ (\hat{\Pi} - \Pi) + G_{\pi_2\tau}^{-1}(\bar{T}, \bar{\pi}) \circ (\hat{T} - T) \circ (\hat{\Pi} - \Pi)^\top \\
&\quad + G_{\pi_1\pi_1}^{-1}(\bar{T}, \bar{\pi}) \circ (\tilde{\Pi} - \Pi) \circ (\hat{\Pi} - \Pi) + G_{\pi_2\pi_2}^{-1}(\bar{T}, \bar{\pi}) \circ (\tilde{\Pi} - \Pi)^\top \circ (\hat{\Pi} - \Pi)^\top \\
&\quad + G_{\pi_1\pi_2}^{-1}(\bar{T}, \bar{\pi}) \circ (\hat{\Pi} - \Pi)^\top \circ (\hat{\Pi} - \Pi) + G_{\pi_2\pi_1}^{-1}(\bar{T}, \bar{\pi}) \circ (\hat{\Pi} - \Pi) \circ (\hat{\Pi} - \Pi)^\top,
\end{aligned} \tag{A28}$$

where $\bar{\tau}_{jk} \in (\tilde{\tau}_{jk}, \tau_{jk})$, $\tilde{\tau}_{jk} \in (\hat{\tau}_{jk}, \tau_{jk})$, $\bar{\pi}_j \in (\tilde{\pi}_j, \pi_j)$, and $\tilde{\pi}_j \in (\hat{\pi}_j, \pi_j)$. We will use one technique to bound all first order terms in (A28), and another technique to bound all second-order terms.

Consider second-order terms in (A28). Each term is bounded in the same way using Hölder's inequality and bounds on second derivatives. Concretely, consider the term corresponding to $G_{\tau\tau}^{-1}$, that is

$$|a^\top G_{\tau\tau}^{-1}(\bar{T}, \Delta) \circ (\tilde{T} - T) \circ (\hat{T} - T)a| \leq \|a\|_1^2 \|G_{\tau\tau}^{-1}(\bar{T}, \Delta) \circ (\tilde{T} - T) \circ (\hat{T} - T)\|_\infty.$$

By Lemma A.11, the 2nd derivative is bounded $|G_{\tau\tau}^{-1}| \leq C$, thus, since $\tilde{\tau}_{jk}$ is between $\hat{\tau}_{jk}$ and τ_{jk},

$$|a^\top G_{\tau\tau}^{-1}(\bar{T}, \Delta) \circ (\tilde{T} - T) \circ (\hat{T} - T)a| \leq C\|a\|_1^2 \|\tilde{T} - T\|_\infty \|\hat{T} - T\|_\infty \leq C\|a\|_1^2 \|\hat{T} - T\|_\infty^2.$$

Using the bound (A25) on $\|a\|_1$, the condition that Σ_{22} satisfies RE$(s, 3)$, and by Lemma A.9, it follows that, for any constant $\eta \in (0, 1)$,

$$|a^\top G_{\tau\tau}^{-1}(\bar{T}, \Delta) \circ (\tilde{T} - T) \circ (\hat{T} - T)a| \leq C_1 \|a_S\|_2^2 \frac{s \log(p\eta^{-1})}{n} \leq a^\top \Sigma_{22} a \; C_1 \gamma \frac{s \log(p\eta^{-1})}{n}$$

with probability at least $1 - \eta$. All the remaining 2nd order terms have the same bound as all the second derivatives are bounded, that is $|G_{\pi_j\pi_j}^{-1}| \leq C$ by Lemma A.12, $|G_{\pi_j\tau}^{-1}| \leq C$ by Lemma A.14, and $|G_{\pi_j\pi_k}^{-1}| \leq C$ by Lemma A.13. Also $\|\hat{\pi} - \pi\|_\infty \leq C_1\sqrt{\log(p\eta^{-1})/n}$ with probability at least $1 - \eta$ by Hoeffding's inequality combined with union bound, and $\|\hat{T} - T\|_\infty \leq C_2\sqrt{\log(p\eta^{-1})/n}$ with probability at least $1 - \eta$ by Lemma A.9.

Consider first-order terms in (A28). Each term is bounded in the same way using sub-gaussian properties in Lemma A.5 (for $\hat{\pi}$) and Lemma A.8 (for $\hat{T}$) combined with the

fact that the first derivatives are both bounded and fixed. Concretely, consider the term corresponding to G_τ^{-1}, that is

$$\begin{aligned}\left|a^\top G_\tau^{-1}(T,\Delta)\circ(\hat{T}-T)a\right| &= \left|\left(\sum_{j=1}^p a_j e_j\right)^\top G_\tau^{-1}(T,\Delta)\circ(\hat{T}-T)a\right| \\ &= \left|\sum_{j=1}^p a_j\left\{e_j^\top G_\tau^{-1}(T,\Delta)\circ(\hat{T}-T)a\right\}\right| \\ &\leq \|a\|_1 \max_{1\leq j\leq p}\left|e_j^\top G_\tau^{-1}(T,\Delta)\circ(\hat{T}-T)a\right| \\ &\leq 4\sqrt{s}\|a_{T_0}\|_2 \max_{1\leq j\leq p}|e_j^\top(\hat{T}-T)b_j|,\end{aligned}$$

where $e_j \in \mathbb{R}^p$ be the vector with 1 in the jth component and 0 otherwise, $b_j = a\circ G_\tau^{-1}(T_j,\Delta)$, and the last inequality follows from (A25). From Theorem 6 of Yoon et al. (2020), $|G_\tau^{-1}| \leq C$ for some constant $C > 0$, hence using (A26)

$$\|b_j\|_2 \leq C\|a\|_2 \leq C_1\|a_{T_0}\|_2.$$

Combining this bound with Lemma A.9, and following the proof of Lemma A.1 gives, with probability at least $1-\eta$,

$$\max_j |e_j^\top(\hat{T}-T)b_j| \leq C_2\|a_{T_0}\|_2\sqrt{\frac{\log(p\eta^{-1})}{n}},$$

and, using that Σ_{22} satisfies $RE(s,3)$ gives

$$|a^\top G_\tau^{-1}(T,\Delta)\circ(\hat{T}-T)a| \leq C\|a_{T_0}\|_2^2\sqrt{\frac{s\log(p\eta^{-1})}{n}} \leq a^\top\Sigma_{22}a\ C\gamma\sqrt{\frac{\log(p\eta^{-1})}{n}}.$$

All the remaining first order terms have the same bound as the first derivatives $G_{\pi_j}^{-1}$ are fixed and bounded by Lemma A.10, and $\hat{\pi}$ satisfy Lemma A.5.

Combining the bounds on the first and second-order terms coupled with Assumption 5 gives

$$\left|a^\top(\hat{\Sigma}_{22}-\Sigma_{22})a\right| \leq a^\top\Sigma_{22}aC_3\gamma\sqrt{\frac{s\log(p\eta^{-1})}{n}}$$

with probability at least $1-\eta$. Combining this bound with (A27) gives

$$a^\top\hat{\Sigma}_{22}a \geq a^\top\Sigma_{22}a\left\{1-C_3\gamma\sqrt{\frac{s\log(p\eta^{-1})}{n}}\right\}.$$

with probability at least $1-\eta$. Under the scaling of Assumption 5, $C_3\gamma\sqrt{s\log p/n} = o(1)$, thus it follows that with probability at least $1-\eta$, $\gamma(\hat{\Sigma}) \leq C\gamma$ for some constant $C > 1$. ■

C.4 Supporting lemmas based on existing results

Lemma A.7 (Barber and Kolar (2018) Lemma 4.5) *Let* $Z \sim \mathrm{N}_p(\mu, \Sigma)$. *Then* $\mathrm{sign}(Z) - \mathrm{E}\{\mathrm{sign}(Z)\}$ *is* $\mathsf{C}(\Sigma)$*-subgaussian.*

Lemma A.8 (Barber and Kolar (2018) Lemma E.2) *For fixed* u *and* v *with* $\|u\|_2, \|v\|_2 \leq 1$, *for any* $|t| \leq n/C$,

$$\mathrm{E}\left[\exp\left\{tu^\top \left(\hat{T} - T\right) v\right\}\right] \leq \exp\left(\frac{t^2 C^2}{n}\right).$$

Lemma A.9 (De la Pena and Giné (2012) Theorem 4.1.8) *For any* $\eta \in (0, 1)$,

$$\|\hat{T} - T\|_\infty \leq \sqrt{\frac{4\log\left(2\binom{p}{2}/\eta\right)}{n}}$$

with probability at least $1 - \eta$.

C.5 Bounds on partial derivatives of the inverse bridge function

Lemma A.10 *Let* $G^{-1}(\tau)$ *be the inverse bridge function for TT case, where* $\tau = G_{TT}(r; \Delta_j, \Delta_k)$. *Under Assumptions 1–2,* $|\partial G^{-1}(\tau)/\partial \pi_j| \leq C$ *and* $|\partial G^{-1}(\tau)/\partial \pi_k| \leq C$ *for some constant* $C > 0$.

Proof By the multivariate chain rule, we have

$$\begin{aligned}\frac{\partial G^{-1}(\tau)}{\partial \pi_j} = \frac{\partial G^{-1}(\tau)}{\partial \tau}\frac{\partial \tau}{\partial \Delta_j}\frac{\partial \Delta_j}{\partial \pi_j} &= \frac{\partial G^{-1}(\tau)}{\partial \tau}\frac{\partial G(r;\Delta_j,\Delta_k)}{\partial \Delta_j}\frac{\partial \Delta_j}{\partial \pi_j} \\ &:= A_1 A_2 A_3.\end{aligned} \tag{A29}$$

By Theorem 6 in Yoon et al. (2020), $|A_1| \leq C$. By Lemma A.15, A_2 is bounded. By Lemma A.21, A_3 is bounded. The proof for π_k is analogous. ■

Lemma A.11 *Let* $G^{-1}(\tau)$ *be the inverse bridge function for TT case, where* $\tau = G_{TT}(r; \Delta_j, \Delta_k)$. *Under Assumptions 1– 2,* $|G^{-1}_{\tau\tau} = \partial^2 G^{-1}(\tau)/\partial \tau^2| \leq C$ *for some constant* $C > 0$ *independent of* r, Δ_j, Δ_k.

Proof Let $h(r) = \partial G(r; \Delta_j, \Delta_k)/\partial r$ and consider

$$\begin{aligned}\frac{\partial^2 G^{-1}(\tau)}{\partial \tau^2} &= \frac{\partial}{\partial \tau}\left\{\frac{\partial G(r;\Delta_j,\Delta_k)}{\partial r}\right\}^{-1} = \frac{\partial}{\partial r}\left\{\frac{\partial G(r;\Delta_j,\Delta_k)}{\partial r}\right\}^{-1}\frac{\partial r}{\partial \tau} \\ &= \frac{\partial}{\partial r}\left\{\frac{1}{h(r)}\right\}\left(\frac{\partial \tau}{\partial r}\right)^{-1} = \frac{\partial}{\partial r}\left\{\frac{1}{h(r)}\right\}\left\{\frac{\partial G(r;\Delta_j,\Delta_k)}{\partial r}\right\}^{-1} \\ &= -\frac{1}{h(r)^2}\frac{\partial h(r)}{\partial r}\frac{1}{h(r)} = -\frac{1}{h(r)^3}\frac{\partial h(r)}{\partial r}.\end{aligned}$$

By Theorem 6 of Yoon et al. (2020), $h(r)$ is positive and bounded from below by a positive constant independent of r, Δ_j, Δ_k. By Lemma A.19, $|\partial h(r)/\partial r|$ is bounded above by a positive constant. Thus, we have $|\partial^2 G^{-1}(\tau)/\partial \tau^2| < C$ for some $C > 0$. ∎

Lemma A.12 *Let $G^{-1}(\tau)$ be the inverse bridge function for TT case, where $\tau = G_{TT}(r, \Delta_j, \Delta_k)$. Under Assumptions 1–2, $|G^{-1}_{\pi_j \pi_j} = \partial^2 G^{-1}(\tau)/\partial \pi_j^2| \leq C$ for some constant $C > 0$ independent of r, Δ_j, Δ_k.*

Proof Let $h(r) = \partial G(r; \Delta_j, \Delta_k)/\partial r$ so that $\partial G^{-1}(\tau)/\partial \tau = (\partial G(r; \Delta_j, \Delta_k)/\partial r)^{-1} = 1/h(r)$. By (A29) and multivariate chain rule, we have

$$
\begin{aligned}
\frac{\partial^2 G^{-1}(\tau)}{\partial \pi_j^2} &= \frac{\partial}{\partial \pi_j}\left(\frac{1}{h(r)}\frac{\partial G(r;\Delta_j,\Delta_k)}{\partial \Delta_j}\frac{\partial \Delta_j}{\partial \pi_j}\right) \\
&= \left[\frac{\partial}{\partial \pi_j}\left\{\frac{1}{h(r)}\right\}\right]\frac{\partial G(r;\Delta_j,\Delta_k)}{\partial \Delta_j}\frac{\partial \Delta_j}{\partial \pi_j} + \frac{1}{h(r)}\left[\frac{\partial^2 G(r;\Delta_j,\Delta_k)}{\partial \pi_j \partial \Delta_j}\right]\frac{\partial \Delta_j}{\partial \pi_j} \\
&\quad + \frac{1}{h(r)}\frac{\partial G(r;\Delta_j,\Delta_k)}{\partial \Delta_j}\left[\frac{\partial^2 \Delta_j}{\partial \pi_j^2}\right] \\
&= \left[\frac{\partial \{h(r)\}^{-1}}{\partial \Delta_j}\frac{\partial \Delta_j}{\partial \pi_j}\right]\frac{\partial G(r;\Delta_j,\Delta_k)}{\partial \Delta_j}\frac{\partial \Delta_j}{\partial \pi_j} + \frac{1}{h(r)}\left[\frac{\partial^2 G(r;\Delta_j,\Delta_k)}{\partial \Delta_j^2}\frac{\partial \Delta_j}{\partial \pi_j}\right]\frac{\partial \Delta_j}{\partial \pi_j} \\
&\quad + \frac{1}{h(r)}\frac{\partial G(r;\Delta_j,\Delta_k)}{\partial \Delta_j}\left[\frac{\partial^2 \Delta_j}{\partial \pi_j^2}\right] \\
&= \frac{\partial \{h(r)\}^{-1}}{\partial \Delta_j}\frac{\partial G(r;\Delta_j,\Delta_k)}{\partial \Delta_j}\left(\frac{\partial \Delta_j}{\partial \pi_j}\right)^2 + \frac{1}{h(r)}\frac{\partial^2 G(r;\Delta_j,\Delta_k)}{\partial \Delta_j^2}\left(\frac{\partial \Delta_j}{\partial \pi_j}\right)^2 \\
&\quad + \frac{1}{h(r)}\frac{\partial G(r;\Delta_j,\Delta_k)}{\partial \Delta_j}\frac{\partial^2 \Delta_j}{\partial \pi_j^2}.
\end{aligned}
$$

We next show that each term is bounded.

Consider $\partial\{h(r)\}^{-1}/\partial \Delta_j$. By the multivariate chain rule,

$$
\frac{\partial \{h(r)\}^{-1}}{\partial \Delta_j} = -\frac{1}{h(r)^2}\frac{\partial h(r)}{\partial \Delta_j} = -\frac{1}{h(r)^2}\frac{\partial^2 G(r;\Delta_j,\Delta_k)}{\partial \Delta_j \partial r}.
$$

The term $|\partial^2 G(r; \Delta_j, \Delta_k)/\partial \Delta_j \partial r|$ is bounded from above by Lemma A.16, and $|1/h(r)|$ is bounded from above by Theorem 6 in Yoon et al. (2020). Furthermore, $|\partial G(r; \Delta_j, \Delta_k)/\partial \Delta_j|$ is bounded by Lemma A.15, $|\partial^2 G(r; \Delta_j, \Delta_k)/\partial \Delta_j^2|$ is bounded by Lemma A.17, and $|\partial \Delta_j/\partial \pi_j|$, $|\partial^2 \Delta_j/\partial \pi_j^2|$ are both bounded by Lemma A.21. This concludes the proof. ∎

Lemma A.13 *Let $G^{-1}(\tau)$ be the inverse bridge function for TT case, where $\tau = G_{TT}(r, \Delta_j, \Delta_k)$. Under Assumptions 1–2, $|\partial^2 G^{-1}(\tau)/\partial \pi_k \pi_j| \leq C$ for some constant $C > 0$ independent of r, Δ_j, Δ_k.*

Proof Let $h(r) = \partial G(r; \Delta_j, \Delta_k)/\partial r$ so that $\partial G^{-1}(\tau)/\partial\tau = (\partial G(r; \Delta_j, \Delta_k)/\partial r)^{-1} = 1/h(r)$. By (A29) and multivariate chain rule, we have

$$\begin{aligned}\frac{\partial^2 G^{-1}(\tau)}{\partial \pi_k \pi_j} =& \frac{\partial}{\partial \pi_k}\left(\frac{1}{h(r)}\frac{\partial G(r;\Delta_j,\Delta_k)}{\partial \Delta_j}\frac{\partial \Delta_j}{\partial \pi_j}\right) \\ &= \left[\frac{\partial}{\partial \pi_k}\left\{\frac{1}{h(r)}\right\}\right]\frac{\partial G(r;\Delta_j,\Delta_k)}{\partial \Delta_j}\frac{\partial \Delta_j}{\partial \pi_j} + \frac{1}{h(r)}\left[\frac{\partial^2 G(r;\Delta_j,\Delta_k)}{\partial \pi_k \partial \Delta_j}\right]\frac{\partial \Delta_j}{\partial \pi_j} \\ &+ \frac{1}{h(r)}\frac{\partial G(r;\Delta_j,\Delta_k)}{\partial \Delta_j}\left[\frac{\partial^2 \Delta_j}{\partial \pi_k \partial \pi_j}\right].\end{aligned}$$

As $\partial^2\Delta_j/\partial\pi_k\partial\pi_j = 0$, we further have that

$$\begin{aligned}\frac{\partial^2 G^{-1}(\tau)}{\partial \pi_k \pi_j} &= \left[\frac{\partial\{h(r)\}^{-1}}{\partial \Delta_k}\frac{\partial \Delta_k}{\partial \pi_k}\right]\frac{\partial G(r;\Delta_j,\Delta_k)}{\partial \Delta_j}\frac{\partial \Delta_j}{\partial \pi_j} + \frac{1}{h(r)}\left[\frac{\partial^2 G(r;\Delta_j,\Delta_k)}{\partial \Delta_k \partial \Delta_j}\frac{\partial \Delta_k}{\partial \pi_k}\right]\frac{\partial \Delta_j}{\partial \pi_j} \\ &= \frac{\partial\{h(r)\}^{-1}}{\partial \Delta_k}\frac{\partial G(r;\Delta_j,\Delta_k)}{\partial \Delta_j}\left(\frac{\partial \Delta_k}{\partial \pi_k}\frac{\partial \Delta_j}{\partial \pi_j}\right) + \frac{1}{h(r)}\frac{\partial^2 G(r;\Delta_j,\Delta_k)}{\partial \Delta_k \partial \Delta_j}\left(\frac{\partial \Delta_k}{\partial \pi_k}\frac{\partial \Delta_j}{\partial \pi_j}\right),\end{aligned}$$

where $|\partial\Delta_j/\partial\pi_j|$ and $|\partial\Delta_k/\partial\pi_k|$ are bounded above by some constant $C > 0$ by Lemma A.21. Thus, by the triangle inequality,

$$\begin{aligned}\left|\frac{\partial^2 G^{-1}(\tau)}{\partial \pi_k \pi_j}\right| &\le \left|\frac{\partial\{h(r)\}^{-1}}{\partial \Delta_k}\frac{\partial G(r;\Delta_j,\Delta_k)}{\partial \Delta_j}\left(\frac{\partial \Delta_k}{\partial \pi_k}\frac{\partial \Delta_j}{\partial \pi_j}\right)\right| + \left|\frac{1}{h(r)}\frac{\partial^2 G(r;\Delta_j,\Delta_k)}{\partial \Delta_k \partial \Delta_j}\left(\frac{\partial \Delta_k}{\partial \pi_k}\frac{\partial \Delta_j}{\partial \pi_j}\right)\right| \\ &\le C^2\left|\frac{\partial\{h(r)\}^{-1}}{\partial \Delta_k}\frac{\partial G(r;\Delta_j,\Delta_k)}{\partial \Delta_j}\right| + C^2\left|\frac{1}{h(r)}\frac{\partial^2 G(r;\Delta_j,\Delta_k)}{\partial \Delta_k \partial \Delta_j}\right|\end{aligned}$$

We next show that each term is bounded.

Consider $\partial\{h(r)\}^{-1}/\partial\Delta_k$. By the multivariate chain rule,

$$\frac{\partial\{h(r)\}^{-1}}{\partial \Delta_k} = -\frac{1}{h(r)^2}\frac{\partial h(r)}{\partial \Delta_k} = -\frac{1}{h(r)^2}\frac{\partial^2 G(r;\Delta_j,\Delta_k)}{\partial \Delta_k \partial r}.$$

The term $|\partial^2 G(r; \Delta_j, \Delta_k)/\partial\Delta_k\partial r|$ is bounded from above by Lemma A.16, and $|1/h(r)|$ is bounded from above by Theorem 6 in Yoon et al. (2020). Furthermore, $|\partial G(r; \Delta_j, \Delta_k)/\partial\Delta_j|$ is bounded by Lemma A.15, $|\partial^2 G(r; \Delta_j, \Delta_k)/\partial\Delta_k\partial\Delta_j|$ is bounded by Lemma A.18. This concludes the proof. ∎

Lemma A.14 *Let $G^{-1}(\tau)$ be the inverse bridge function for TT case, where $\tau = G_{TT}(r, \Delta_j, \Delta_k)$. Under Assumptions 1–2, $|\partial^2 G^{-1}(\tau)/\partial\pi_j\partial\tau| \le C$ for some constant $C > 0$ independent of r, Δ_j, Δ_k.*

Proof Let $h(r) = \partial G(r; \Delta_j, \Delta_k)/\partial r$ so that $\partial G^{-1}(\tau)/\partial\tau = (\partial G(r; \Delta_j, \Delta_k)/\partial r)^{-1} = 1/h(r)$. By the multivariate chain rule,

$$\begin{aligned}\frac{\partial^2 G^{-1}(\tau)}{\partial \pi_j \partial \tau} =& \frac{\partial}{\partial \pi_j}\frac{\partial G^{-1}(\tau)}{\partial \tau} = \frac{\partial}{\partial \pi_j}\left\{\frac{1}{h(r)}\right\} = \frac{\partial}{\partial \Delta_j}\left\{\frac{1}{h(r)}\right\}\frac{\partial \Delta_j}{\partial \pi_j} \\ &= -\frac{1}{h(r)^2}\frac{\partial h(r)}{\partial \Delta_j}\frac{\partial \Delta_j}{\partial \pi_j} = -\frac{1}{h(r)^2}\frac{\partial G(r;\Delta_j,\Delta_k)}{\partial \Delta_j \partial r}\frac{\partial \Delta_j}{\partial \pi_j}.\end{aligned}$$

The terms $|\partial\Delta_j/\partial\pi_j|$, $|1/h(r)^2|$, and $|\partial^2 G(r;\Delta_j,\Delta_k)/\partial\Delta_j\partial r|$ are bounded above by constants by Lemma A.21, Theorem 6 of Yoon et al. (2020), and Lemma A.16, respectively. Thus, for some constant $C > 0$, we have

$$\left|\frac{\partial^2 G^{-1}(\tau)}{\partial\pi_j\partial\tau}\right| \leq C.$$

■

C.6 Bounds on the partial derivatives of the bridge function

Here we bound partial derivatives of the bridge function $G(r,\Delta_j,\Delta_k)$ for TT case, where

$$G(r,\Delta_j,\Delta_k) = -2\Phi_4(-\Delta_j,-\Delta_k,0,0;\Sigma_{4a}) + 2\Phi_4(-\Delta_j,-\Delta_k,0,0;\Sigma_{4b}).$$

As the bridge function consists of two 4-dimensional Gaussian distribution functions, we will show that, whether $\Sigma_4 = \Sigma_{4a}$ or $\Sigma_4 = \Sigma_{4b}$, the absolute values of partial derivatives of $\Phi_4(-\Delta_j,-\Delta_k,0,0;\Sigma_4)$ are bounded from above.

Lemma A.15 *Under Assumptions 1–2, $|\partial G(r;\Delta_j,\Delta_k)/\partial\Delta_j|$ and $|\partial G(r;\Delta_j,\Delta_k)/\partial\Delta_k|$ are bounded above by some constant $C > 0$ independent from r, Δ_j, Δ_k.*

Proof By the Leibniz rule,

$$\begin{aligned}\frac{\partial}{\partial\Delta_j}\Phi_4(-\Delta_j,-\Delta_k,0,0;\Sigma_4) &= \frac{\partial}{\partial\Delta_j}\int_{-\infty}^{0}\int_{-\infty}^{0}\int_{-\infty}^{-\Delta_k}\int_{-\infty}^{-\Delta_j}\phi(z_1,z_2,z_3,z_4)\mathrm{d}z_1\mathrm{d}z_2\mathrm{d}z_3\mathrm{d}z_4\\ &= (-1)\int_{-\infty}^{0}\int_{-\infty}^{0}\int_{-\infty}^{-\Delta_k}\phi(-\Delta_j,z_2,z_3,z_4)\mathrm{d}z_2\mathrm{d}z_3\mathrm{d}z_4.\end{aligned}$$

Thus, regardless of $\Sigma_4 = \Sigma_{4a}$ or $\Sigma_4 = \Sigma_{4b}$,

$$\begin{aligned}\left|\frac{\partial}{\partial\Delta_j}\Phi_4(-\Delta_j,-\Delta_k,0,0;\Sigma_4)\right| &= \int_{-\infty}^{0}\int_{-\infty}^{0}\int_{-\infty}^{-\Delta_k}\phi(-\Delta_j,z_2,z_3,z_4)\mathrm{d}z_2\mathrm{d}z_3\mathrm{d}z_4\\ &= \phi(-\Delta_j)\int_{-\infty}^{0}\int_{-\infty}^{0}\int_{-\infty}^{-\Delta_k}\phi(z_2,z_3,z_4\mid -\Delta_j)\mathrm{d}z_2\mathrm{d}z_3\mathrm{d}z_4,\end{aligned}$$

where $\phi(z_2,z_3,z_4 \mid -\Delta_j)$ is the conditional pdf given $Z_1 = -\Delta_j$. Therefore, the three-dimensional integral above corresponds to a probability (and is bounded by one), leading to

$$\left|\frac{\partial}{\partial\Delta_j}\Phi_4(-\Delta_j,-\Delta_k,0,0;\Sigma_4)\right| \leq \phi(-\Delta_j) \leq \phi(0) = 1/\sqrt{2\pi}.$$

The proof for Δ_k follows analogously. ■

Lemma A.16 *Under Assumptions 1–2, $|\partial^2 G(r;\Delta_j,\Delta_k)|/\partial r\partial\Delta_j|$ and $|\partial^2 G(r;\Delta_j,\Delta_k)|/\partial r\partial\Delta_k|$ are bounded above by some constant $C > 0$.*

Proof We start from the partial derivative with respect to Δ_j given in Lemma A.15 as

$$\frac{\partial}{\partial \Delta_j}\Phi_4(-\Delta_j,-\Delta_k,0,0;\Sigma_4)=(-1)\int_{-\infty}^{0}\int_{-\infty}^{0}\int_{-\infty}^{-\Delta_k}\phi(-\Delta_j,z_2,z_3,z_4;\Sigma_4)\mathrm{d}z_2\mathrm{d}z_3\mathrm{d}z_4.$$

Let $\Sigma_4=[\rho_{jk}]_{1\le j,k\le 4}$ and consider the following multivariate chain rule:

$$\begin{aligned}
&\frac{\partial}{\partial r}\int_{-\infty}^{0}\int_{-\infty}^{0}\int_{-\infty}^{-\Delta_k}\phi(-\Delta_j,z_2,z_3,z_4;\Sigma_4)\mathrm{d}z_2\mathrm{d}z_3\mathrm{d}z_4\\
&\qquad=\sum_{j<k}\left\{\frac{\partial}{\partial \rho_{jk}}\int_{-\infty}^{0}\int_{-\infty}^{0}\int_{-\infty}^{-\Delta_k}\phi(-\Delta_j,z_2,z_3,z_4;\Sigma_4)\mathrm{d}z_2\mathrm{d}z_3\mathrm{d}z_4\frac{\partial\rho_{jk}}{\partial r}\right\}\\
&\qquad=\sum_{j<k}\left\{\int_{-\infty}^{0}\int_{-\infty}^{0}\int_{-\infty}^{-\Delta_k}\frac{\partial\phi(-\Delta_j,z_2,z_3,z_4;\Sigma_4)}{\partial\rho_{jk}}\mathrm{d}z_2\mathrm{d}z_3\mathrm{d}z_4\frac{\partial\rho_{jk}}{\partial r}\right\}.
\end{aligned}$$

In the above, we only consider partial derivatives with respect to ρ_{12}, ρ_{14}, ρ_{23}, and ρ_{34} because ρ_{13}, ρ_{24} do not involve r whether $\Sigma_4=\Sigma_{4a}$ or $\Sigma_4=\Sigma_{4b}$, i.e., $\partial\rho_{jk}/\partial r=0$.

Consider the case $(j,k)=(2,3)$. By Plackett (1954),

$$\begin{aligned}
&\int_{-\infty}^{0}\int_{-\infty}^{0}\int_{-\infty}^{-\Delta_k}\frac{\partial\phi(-\Delta_j,z_2,z_3,z_4;\Sigma_4)}{\partial\rho_{23}}\mathrm{d}z_2\mathrm{d}z_3\mathrm{d}z_4\\
&\qquad=\int_{-\infty}^{0}\int_{-\infty}^{0}\int_{-\infty}^{-\Delta_k}\frac{\partial^2\phi(-\Delta_j,z_2,z_3,z_4;\Sigma_4)}{\partial z_2\partial z_3}\mathrm{d}z_2\mathrm{d}z_3\mathrm{d}z_4\\
&\qquad=\int_{-\infty}^{0}\phi(-\Delta_j,-\Delta_k,0,z_4;\Sigma_4)\mathrm{d}z_4\\
&\qquad=\int_{-\infty}^{0}\phi(z_4\mid-\Delta_j,-\Delta_k,0)\phi(-\Delta_j,-\Delta_k,0)\mathrm{d}z_4\\
&\qquad=\phi(-\Delta_j,-\Delta_k,0)\int_{-\infty}^{0}\phi(z_4\mid\Delta_j,-\Delta_k,0)\mathrm{d}z_4,
\end{aligned}$$

where $\phi(z_4|-\Delta_j,-\Delta_k,0)$ is the conditional pdf given $Z_1=-\Delta_j$, $Z_2=-\Delta_k$, and $Z_3=0$. Therefore, above integral corresponds to a probability (and is bounded by one), leading to

$$\int_{-\infty}^{0}\int_{-\infty}^{0}\int_{-\infty}^{-\Delta_k}\frac{\partial\phi(-\Delta_j,z_2,z_3,z_4;\Sigma_4)}{\partial\rho_{23}}\mathrm{d}z_2\mathrm{d}z_3\mathrm{d}z_4\le\phi(-\Delta_j,-\Delta_k,0)\le|\Sigma_4|^{-1/2},$$

where above inequalities hold because $\phi(-\Delta_j,-\Delta_k,0)\le\phi(0,0,0)\le|\Sigma_3|^{-1/2}\le|\Sigma_4|^{-1/2}$ and $\Sigma_3=\mathrm{var}\{(Z_1,Z_2,Z_3)\}$. As Lemma A.20 provides that $|\Sigma_4|^{-1/2}\le C$ for some constant $C>0$, we have the desired result. The case $(j,k)=(3,4)$ is similar with the same bound.

For $(j,k)=(1,2)$, again by Plackett (1954), we have

$$\int_{-\infty}^{0}\int_{-\infty}^{0}\int_{-\infty}^{-\Delta_k}\frac{\partial\phi(-\Delta_j,z_2,z_3,z_4)}{\partial\rho_{12}}\mathrm{d}z_2\mathrm{d}z_3\mathrm{d}z_4=-\int_{-\infty}^{0}\int_{-\infty}^{0}\frac{\partial\phi(-\Delta_j,-\Delta_k,z_3,z_4)}{\partial\Delta_j}\mathrm{d}z_3\mathrm{d}z_4.$$

For notational convenience, let $y = (-\Delta_j, -\Delta_k, z_3, z_4)^\top = (y_1, y_2, y_3, y_4)^\top$ and write

$$\int_{-\infty}^{0}\int_{-\infty}^{0} \frac{\partial \phi(-\Delta_j, -\Delta_k, z_3, z_4)}{\partial \Delta_j} \mathrm{d}z_3 \mathrm{d}z_4 = \int_{-\infty}^{0}\int_{-\infty}^{0} \frac{\partial \phi(y_1, y_2, y_3, y_4)}{\partial y_1} \mathrm{d}y_3 \mathrm{d}y_4$$
$$= \int_{-\infty}^{0}\int_{-\infty}^{0} (-\omega_1^\top y)\phi(y_1, y_2, y_3, y_4) \mathrm{d}y_3 \mathrm{d}y_4, \quad \text{(A30)}$$

where $\omega_i^\top$ is the ith row of Σ_4^{-1}. Then, by extending the range of integrations, the absolute value of (A30) is bounded above as

$$\left|\int_{-\infty}^{0}\int_{-\infty}^{0} (-\omega_1^\top y)\phi(y_1, y_2, y_3, y_4) \mathrm{d}y_3 \mathrm{d}y_4\right| \leq \int_{-\infty}^{0}\int_{-\infty}^{0} \left|\omega_1^\top y\right| \phi(y_1, y_2, y_3, y_4) \mathrm{d}y_3 \mathrm{d}y_4$$
$$\leq \int_{-\infty}^{\infty}\int_{-\infty}^{\infty} \left|\omega_1^\top y\right| \phi(y_1, y_2, y_3, y_4) \mathrm{d}y_3 \mathrm{d}y_4.$$

By the triangle inequality,

$$\int_{-\infty}^{\infty}\int_{-\infty}^{\infty} \left|\omega_1^\top y\right| \phi(y_1, y_2, y_3, y_4) \mathrm{d}y_3 \mathrm{d}y_4$$
$$= \int_{-\infty}^{\infty}\int_{-\infty}^{\infty} \sum_{i'=1}^{4} |\omega_{1i'} y_{i'}| \, \phi(y_1, y_2, y_3, y_4) \mathrm{d}y_3 \mathrm{d}y_4$$
$$\leq \sum_{i'=1}^{2} |\omega_{1i'} y_{i'}| \phi(y_1, y_2) + \sum_{i'=3}^{4} |\omega_{1i'}| \int_{-\infty}^{\infty}\int_{-\infty}^{\infty} |y_{i'}| \phi(y_1, y_2, y_3, y_4) \mathrm{d}y_3 \mathrm{d}y_4$$
$$\leq |\Sigma_4|^{-1/2} \left\{ \sum_{i'=1}^{2} |\omega_{1i'} y_{i'}| + \sum_{i'=3}^{4} |\omega_{1i'}| \int_{-\infty}^{\infty}\int_{-\infty}^{\infty} |y_{i'}| \phi(y_3, y_4 \mid y_1, y_2) \mathrm{d}y_3 \mathrm{d}y_4 \right\},$$

where the last inequality holds as $\phi(y_1, y_2) \leq |\Sigma_2|^{-1/2} \leq |\Sigma_4|^{-1/2}$ and $\Sigma_2 = \mathrm{var}\{(Y_1, Y_2)\}$. Under Assumption 2, $|y_1| = |\Delta_j| \leq M$,$|y_2| = |\Delta_k| \leq M$. By Lemma A.20, whether $\Sigma_4 = \Sigma_{4a}$ or $\Sigma_4 = \Sigma_{4b}$, $|\Sigma_4|^{-1/2}$ is bounded above and all elements of $\Sigma_4^{-1} = [\omega_{\ell\ell'}]_{1 \leq \ell\ell' \leq 4}$ are all bounded above. Thus, we have

$$\int_{-\infty}^{\infty}\int_{-\infty}^{\infty} \left|\omega_1^\top y\right| \phi(y_1, y_2, y_3, y_4) \mathrm{d}y_3 \mathrm{d}y_4 \leq C_0 + C_1 \sum_{i'=3}^{4} \int_{-\infty}^{\infty}\int_{-\infty}^{\infty} |y_{i'}| \phi(y_3, y_4 \mid y_1, y_2) \mathrm{d}y_3 \mathrm{d}y_4.$$

By Lemma A.23, for some constant $C > 0$,

$$\sum_{i'=3}^{4} \int_{-\infty}^{\infty}\int_{-\infty}^{\infty} |y_{i'}| \phi(y_3, y_4 \mid y_1, y_2) \mathrm{d}y_3 \mathrm{d}y_4 = \sum_{i'=3}^{4} \mathrm{E}(Y_{i'} \mid Y_1 = y_1, Y_2 = y_2) \leq C.$$

This concludes the proof and the proof for Δ_k is analogous. ■

Lemma A.17 *Under Assumptions 1 and 2,* $|\partial^2 G(r; \Delta_j, \Delta_k)/\partial \Delta_j^2|$ *and* $|\partial^2 G(r; \Delta_j, \Delta_k)/\partial \Delta_k^2|$ *are bounded above by some constant* $C > 0$.

Proof From the proof of Lemma A.15, we have

$$\frac{\partial}{\partial \Delta_j}\Phi_4(-\Delta_j, -\Delta_k, 0, 0; \Sigma_4) = (-1)\int_{-\infty}^{0}\int_{-\infty}^{0}\int_{-\infty}^{-\Delta_k} \phi(-\Delta_j \mid z_2, z_3, z_4)\phi(z_2, z_3, z_4)\mathrm{d}z_2\mathrm{d}z_3\mathrm{d}z_4.$$

By interchanging differentiation and integration,

$$\begin{aligned}\frac{\partial^2}{\partial \Delta_j^2}\Phi_4(-\Delta_j, -\Delta_k, 0, 0; \Sigma_4) =&(-1)\int_{-\infty}^{0}\int_{-\infty}^{0}\int_{-\infty}^{-\Delta_k} \frac{\partial}{\partial \Delta_j}\phi(-\Delta_j \mid z_2, z_3, z_4)\phi(z_2, z_3, z_4)\mathrm{d}z_2\mathrm{d}z_3\mathrm{d}z_4 \\ =&\int_{-\infty}^{0}\int_{-\infty}^{0}\int_{-\infty}^{-\Delta_k} \frac{\Delta_j+\mu}{v^2}\phi(-\Delta_j \mid z_2, z_3, z_4)\phi(z_2, z_3, z_4)\mathrm{d}z_2\mathrm{d}z_3\mathrm{d}z_4,\end{aligned}$$

where $\mathrm{E}(z_1 \mid z_2, z_3, z_4) = \mu$ and $\mathrm{var}(z_1 \mid z_2, z_3, z_4) = v^2$ as in Lemma A.22. Thus

$$\left|\frac{\partial^2}{\partial \Delta_j^2}\Phi_4(-\Delta_j, -\Delta_k, 0, 0; \Sigma_4)\right| \leq \int_{-\infty}^{0}\int_{-\infty}^{0}\int_{-\infty}^{-\Delta_k} \left|\frac{\Delta_j}{v^2}\right| \phi(-\Delta_j \mid z_2, z_3, z_4)\phi(z_2, z_3, z_4)\mathrm{d}z_2\mathrm{d}z_3\mathrm{d}z_4 \tag{A31}$$

$$+\int_{-\infty}^{0}\int_{-\infty}^{0}\int_{-\infty}^{-\Delta_k} \left|\frac{\mu}{v^2}\right| \phi(-\Delta_j \mid z_2, z_3, z_4)\phi(z_2, z_3, z_4)\mathrm{d}z_2\mathrm{d}z_3\mathrm{d}z_4. \tag{A32}$$

Consider the first term (A31). Following the proof of Lemma A.15,

$$\begin{aligned}\int_{-\infty}^{0}\int_{-\infty}^{0}\int_{-\infty}^{-\Delta_k} &\left|\frac{\Delta_j}{v^2}\right| \phi(-\Delta_j \mid z_2, z_3, z_4)\phi(z_2, z_3, z_4)\mathrm{d}z_2\mathrm{d}z_3\mathrm{d}z_4 \\ &= \left|\frac{\Delta_j}{v^2}\right| \int_{-\infty}^{0}\int_{-\infty}^{0}\int_{-\infty}^{-\Delta_k} \phi(-\Delta_j, z_2, z_3, z_4)\mathrm{d}z_2\mathrm{d}z_3\mathrm{d}z_4 \\ &\leq \left|\frac{\Delta_j}{v^2}\right| \frac{1}{\sqrt{2\pi}} \leq C, \end{aligned} \tag{A33}$$

where the last inequality holds as $|\Delta_j| \leq M$ under Assumption 2, and v^2 is bounded below by Lemma A.22.

Consider the second term (A32). Let $z_{-1} = (z_2, z_3, z_4)^\top$ and write $\mu = rz_2 + z_3/2^{1/2} - rz_4/2^{1/2} = u^\top z_{-1}$ as in Lemma A.22. Then, since $\phi(-\Delta_j \mid z_2, z_3, z_4) \leq 1/\sqrt{2\pi v^2}$,

$$\begin{aligned}\int_{-\infty}^{0}\int_{-\infty}^{0}\int_{-\infty}^{-\Delta_k} &\left|\frac{\mu}{v^2}\right| \phi(-\Delta_j \mid z_2, z_3, z_4)\phi(z_2, z_3, z_4)\mathrm{d}z_2\mathrm{d}z_3\mathrm{d}z_4 \\ &\leq \frac{1}{v^3\sqrt{2\pi}}\int_{-\infty}^{0}\int_{-\infty}^{0}\int_{-\infty}^{-\Delta_k} |u^\top \boldsymbol{z}_{-1}|\phi(z_2, z_3, z_4)\mathrm{d}z_2\mathrm{d}z_3\mathrm{d}z_4.\end{aligned}$$

Since $u^\top z_{-1} \sim \mathrm{N}(0, \frac{1+r^2}{2})$ and $|u^\top z_{-1}|$ follows the folded Gaussian with mean $\mathrm{E}|u^\top z_{-1}| = \sqrt{(1+r^2)/\pi}$, we further have that

$$\begin{aligned}\int_{-\infty}^{0}\int_{-\infty}^{0}\int_{-\infty}^{-\Delta_k} \left|\frac{\mu}{v^2}\right| \phi(-\Delta_j \mid z_2, z_3, z_4)\phi(z_2, z_3, z_4)\mathrm{d}z_2\mathrm{d}z_3\mathrm{d}z_4 &\leq \frac{1}{v^3\sqrt{2\pi}}\mathrm{E}|u^\top z_{-1}| \\ &= \frac{1}{v^3\pi}\left(\frac{1+r^2}{2}\right)^{1/2} \\ &\leq C,\end{aligned}$$

where the last inequality holds as $|r| \leq 1 - \varepsilon_r$ and v^3 is bounded below by Assumption 1. ■

Lemma A.18 *Under Assumptions 1 and 2, $|\partial^2 G(r; \Delta_j, \Delta_k)/\partial\Delta_k\partial\Delta_j|$ is bounded above by some constant $C > 0$.*

Proof By the Leibniz rule,

$$\begin{aligned}\frac{\partial^2}{\partial\Delta_k\partial\Delta_j}\Phi_4(-\Delta_j, -\Delta_k, 0, 0; \Sigma_4) &= \int_{-\infty}^{0}\int_{-\infty}^{0} \phi(z_3, z_4 \mid -\Delta_j, -\Delta_k)\phi(-\Delta_j, -\Delta_k)\mathrm{d}z_3\mathrm{d}z_4 \\ &= \phi(-\Delta_j, -\Delta_k)\int_{-\infty}^{0}\int_{-\infty}^{0} \phi(z_3, z_4 \mid -\Delta_j, -\Delta_k)\mathrm{d}z_3\mathrm{d}z_4,\end{aligned}$$

where $\phi(z_3, z_4 \mid -\Delta_j, -\Delta_k)$ is the conditional pdf given $Z_1 = -\Delta_j$ and $Z_2 = -\Delta_k$. Thus, the two-dimensional integral above corresponds to a probability (and is bounded by one), leading to

$$\left|\frac{\partial^2}{\partial\Delta_k\partial\Delta_j}\Phi_4(-\Delta_j, -\Delta_k, 0, 0; \Sigma_4)\right| \leq \phi(-\Delta_j, -\Delta_k) \leq \phi(0, 0) = |\Sigma_2|^{-1/2} \leq |\Sigma_4|^{-1/2},$$

where $\Sigma_2 = \mathrm{var}\{(Z_1, Z_2)\}$. As Lemma A.20 provides that $|\Sigma_4|^{-1/2} \leq C$ for some constant $C > 0$, this concludes the proof. ■

Lemma A.19 *Under Assumptions 1 and 2, $|\partial^2 G(r; \Delta_j, \Delta_k)/\partial r^2|$ is bounded above by some constant $C > 0$.*

Proof We start from the partial derivative with respect to r given in Theorem 6 of Yoon et al. (2020) as

$$\begin{aligned}\frac{\partial G(r, \Delta_j, \Delta_k)}{\partial r} &= -2\frac{\partial\Phi_4\{\Delta_j, \Delta_k, 0, 0; \Sigma_{4a}(r)\}}{\partial r} + 2\frac{\partial\Phi_4\{\Delta_j, \Delta_k, 0, 0; \Sigma_{4b}(r)\}}{\partial r} \\ &= 2^{1/2}h_{14a}(r) + 2^{1/2}h_{23a}(r) + 2h_{23a}(r) + 2h_{12b}(r) + 2^{1/2}h_{14b}(r) + 2^{1/2}h_{23b}(r) + 2h_{34b}(r),\end{aligned}$$

where $h_{14a}(r)$ is defined as

$$h_{14a}(r) = \frac{\partial\Phi(a_1, \ldots, a_4; \Sigma_{4a})}{\partial\rho_{14}(r)} = \int_{-\infty}^{a_3}\int_{-\infty}^{a_2} \phi(a_1, y_2, y_3, a_4; \Sigma_4)\mathrm{d}y_2\mathrm{d}y_3$$

and the rest of $h_{ij}(r)$'s are analogously defined.

As $\partial G(r; \Delta_j, \Delta_k)/\partial r$ is a sum of $h_{ij}(r)$'s, we show that $|\partial h_{ij}(r)/\partial r|$ is bounded above for all i and j whether $\Sigma_4 = \Sigma_{4a}$ and $\Sigma_4 = \Sigma_{4b}$. Using the multivariate chain rule and triangle inequality,

$$\left|\frac{\partial h_{ij}(r)}{\partial r}\right| = \left|\sum_{k<\ell}\frac{\partial h_{ij}(r)}{\partial\rho_{k\ell}}\frac{\partial\rho_{k\ell}}{\partial r}\right| \leq \sum_{k<\ell}\left|\frac{\partial h_{ij}(r)}{\partial\rho_{k\ell}}\right|\left|\frac{\partial\rho_{k\ell}}{\partial r}\right|.$$

By Lemma A.26, for all $1 \leq i < j \leq 4$ and $1 \leq k < \ell \leq 4$, $|\partial h_{ij}(r)/\partial\rho_{k\ell}| \leq C$ for some constant $C > 0$. Also, as $\rho_{k\ell}$'s are linear in r, $|\partial\rho_{k\ell}/\partial r|$'s are bounded above some positive constant. This concludes the proof. ■

C.7 Auxiliary lemmas

From Theorem 4 in Yoon et al. (2020), the bridge function for TT case takes the following form

$$G(r, \Delta_j, \Delta_k) = -2\Phi_4(-\Delta_j, -\Delta_k, 0, 0, ; \Sigma_{4a}) + 2\Phi_4(-\Delta_j, -\Delta_k, 0, 0, ; \Sigma_{4b})$$

with $\Delta_j = f_j(D_j)$, $\Delta_k = f_k(D_k)$,

$$\Sigma_{4a} = \begin{pmatrix} 1 & 0 & 1/\sqrt{2} & -r/\sqrt{2} \\ 0 & 1 & -r/\sqrt{2} & 1/\sqrt{2} \\ 1/\sqrt{2} & -r/\sqrt{2} & 1 & -r \\ -r/\sqrt{2} & 1/\sqrt{2} & -r & 1 \end{pmatrix}, \quad \Sigma_{4b} = \begin{pmatrix} 1 & r & 1/\sqrt{2} & r/\sqrt{2} \\ r & 1 & r/\sqrt{2} & 1/\sqrt{2} \\ 1/\sqrt{2} & r/\sqrt{2} & 1 & r \\ r/\sqrt{2} & 1/\sqrt{2} & r & 1 \end{pmatrix}. \tag{A34}$$

Lemma A.20 *Let $\Sigma_4 = \Sigma_{4a}$ or $\Sigma_4 = \Sigma_{4b}$ from above, and let its inverse be $\Sigma_4^{-1} = [\omega_{\ell\ell'}]_{1\le\ell,\ell'\le4}$. Under Assumption 1, $|\omega_{\ell\ell'}| \le C_1$, $1 \le \ell, \ell' \le 4$, for some constant $C_1 > 0$. Also, $|\Sigma_4|^{-1} \le C_2$ for some constant $C_2 > 0$ regardless of $\Sigma_4 = \Sigma_{4a}$ or $\Sigma_4 = \Sigma_{4b}$.*

Proof Computing determinants gives $|\Sigma_{4a}| = |\Sigma_{4b}| = (1-r^2)^2/4$, and thus by Assumption 1, $|\Sigma_4| \ge \{1 - (1 - \varepsilon_r^2)^2\}/4$, whether $\Sigma_4 = \Sigma_{4a}$ or $\Sigma_4 = \Sigma_{4b}$. Also, the inverses of Σ_{4a} and Σ_{4b} are

$$\Sigma_{4a}^{-1} = \frac{1}{r^2-1}\begin{pmatrix} -2 & 2r & \sqrt{2} & -\sqrt{2}r \\ 2r & -2 & -\sqrt{2}r & \sqrt{2} \\ \sqrt{2} & -\sqrt{2}r & -2 & 0 \\ -\sqrt{2}r & \sqrt{2} & 0 & -2 \end{pmatrix}, \quad \Sigma_{4b}^{-1} = \frac{1}{r^2-1}\begin{pmatrix} -2 & 2r & \sqrt{2} & -\sqrt{2}r \\ 2r & -2 & -\sqrt{2}r & \sqrt{2} \\ \sqrt{2} & -\sqrt{2}r & -2 & 2r \\ -\sqrt{2}r & \sqrt{2} & 2r & -2 \end{pmatrix},$$

respectively. Under Assumption 1, $|\omega_{\ell\ell'}|$'s are all bounded above by $2/\{1 - (1 - \varepsilon_r)^2\}$. ■

Lemma A.21 *Let $\Delta = \Phi^{-1}(\pi)$. Then, under Assumption 2,*

$$\left|\frac{\partial\Delta}{\partial\pi}\right| \le C_1 \quad \text{and} \quad \left|\frac{\partial^2\Delta}{\partial\pi^2}\right| \le C_2$$

for some constants $C_1, C_2 > 0$.

Proof Since $|\Delta| \le M$ and $\phi(|x|)$ is a decreasing function,

$$\frac{\partial\Delta}{\partial\pi} = \left(\frac{\partial\pi}{\partial\Delta}\right)^{-1} = \left\{\frac{\partial\Phi(\Delta)}{\partial\Delta}\right\}^{-1} = \frac{1}{\phi(\Delta)} \le \frac{1}{\phi(M)}.$$

Furthermore, as the second derivative is

$$\frac{\partial^2\Delta}{\partial\pi^2} = \frac{\partial}{\partial\pi}\frac{1}{\phi(\Delta)} = \frac{\partial\Delta}{\partial\pi}\frac{\partial}{\partial\Delta}\frac{1}{\phi(\Delta)} = -\frac{1}{\{\phi(\Delta)\}^3}\frac{\partial\phi(\Delta)}{\partial\Delta} = \frac{\Delta}{\{\phi(\Delta)\}^2},$$

we have

$$\left|\frac{\partial^2 \Delta}{\partial \pi^2}\right| \leq \frac{M}{\{\phi(M)\}^2}.$$

■

Lemma A.22 *Let* $(Z_1, Z_2, Z_3, Z_4)^\top \sim \mathrm{N}_4(0, \Sigma_4)$. *Then, it follows that regardless of* $\Sigma_4 = \Sigma_{4a}$ *or* $\Sigma_4 = \Sigma_{4b}$*, the conditional distribution of* Z_1 *given* Z_2, Z_3, Z_4 *is* $\mathrm{N}(\mu, v^2)$*, where*

$$\mu := \mathrm{E}(Z_1 \mid Z_2, Z_3, Z_4) = rZ_2 + Z_3/2^{1/2} - rZ_4/2^{1/2}$$
$$v^2 := \mathrm{var}(Z_1 \mid Z_2, Z_3, Z_4) = (1 - r^2)/2.$$

Proof The results follow from the properties of conditional Gaussian distribution using the form of Σ_{4a} and Σ_{4b} (A34). ■

Lemma A.23 *Let* $Y \sim \mathrm{N}_4(0, \Sigma_4)$*, where* $\Sigma_4 = \Sigma_{4a}$ *or* $\Sigma_4 = \Sigma_{4b}$. *Then, under Assumptions 1 and 2, for any* $1 \leq k < \ell \leq 4$ *and* $1 \leq i \leq 4$*,*

$$\mathrm{E}\left(|Y_i| \mid Y_k = y_k, Y_\ell = y_\ell\right) \leq C_0, \quad 0 < C_1 \leq \mathrm{var}\left(Y_i \mid Y_k = y_k, Y_\ell = y_\ell\right) \leq C_2$$

for some $C_0, C_1, C_2 > 0$*, where*

$$y_m = \begin{cases} -\Delta_j, & \textit{if} \quad m = 1; \\ -\Delta_k, & \textit{if} \quad m = 2; \\ 0, & \textit{otherwise.} \end{cases}$$

Proof We first calculate the conditional means and covariance matrices using the properties of multivariate Gaussian distribution to obtain:

$$\mathrm{E}(Y_1, Y_2 \mid Y_3 = 0, Y_4 = 0; \Sigma_{4a}) = \mathrm{E}(Y_1, Y_2 \mid Y_3 = 0, Y_4 = 0; \Sigma_{4b}) = \begin{pmatrix} 0 \\ 0 \end{pmatrix},$$

$$\mathrm{E}(Y_1, Y_3 \mid Y_2 = -\Delta_k, Y_4 = 0; \Sigma_{4a}) = \mathrm{E}(Y_1, Y_3 \mid Y_2 = -\Delta_k, Y_4 = 0; \Sigma_{4a}) = \begin{pmatrix} -\Delta_k r \\ 0 \end{pmatrix},$$

$$\mathrm{E}(Y_1, Y_4 \mid Y_2 = -\Delta_k, Y_3 = 0; \Sigma_{4a}) = \mathrm{E}(Y_1, Y_4 \mid Y_2 = -\Delta_k, Y_3 = 0; \Sigma_{4b}) = \frac{1}{2 - r^2} \begin{pmatrix} -\Delta_k r \\ -\sqrt{2}\Delta_k(1 - r^2) \end{pmatrix},$$

$$\mathrm{E}(Y_2, Y_3 \mid Y_1 = -\Delta_j, Y_4 = 0; \Sigma_{4a}) = \mathrm{E}(Y_2, Y_3 \mid Y_1 = -\Delta_j, Y_4 = 0; \Sigma_{4a}) = \frac{1}{2 - r^2} \begin{pmatrix} -\Delta_j r \\ -\sqrt{2}\Delta_j(1 - r^2) \end{pmatrix},$$

$$\mathrm{E}(Y_2, Y_4 \mid Y_1 = -\Delta_j, Y_3 = 0; \Sigma_{4a}) = \mathrm{E}(Y_2, Y_4 \mid Y_1 = -\Delta_j, Y_3 = 0; \Sigma_{4b}) = \begin{pmatrix} -\Delta_j r \\ 0 \end{pmatrix}$$

$$\mathrm{E}(Y_3, Y_4 \mid Y_1 = -\Delta_j, Y_2 = -\Delta_k; \Sigma_{4a}) = \frac{1}{\sqrt{2}} \begin{pmatrix} \Delta_k r - \Delta_j \\ \Delta_j r - \Delta_k \end{pmatrix},$$

$$\mathrm{E}(Y_3, Y_4 \mid Y_1 = -\Delta_j, Y_2 = -\Delta_k; \Sigma_{4b}) = \frac{1}{\sqrt{2}} \begin{pmatrix} -\Delta_j \\ -\Delta_k \end{pmatrix},$$

and

$$\begin{aligned}
&\mathrm{var}(Y_1, Y_2|Y_3, Y_4; \Sigma_{4a}) = \mathrm{var}(Y_1, Y_2|Y_3, Y_4; \Sigma_{4b}) = \frac{1}{2}\begin{pmatrix}1 & r\\ r & 1\end{pmatrix},\\
&\mathrm{var}(Y_1, Y_3|Y_2, Y_4; \Sigma_{4a}) = \mathrm{var}(Y_1, Y_3|Y_2, Y_4; \Sigma_{4b})\\
&= \mathrm{var}(Y_2, Y_4|Y_1, Y_3; \Sigma_{4a}) = \mathrm{var}(Y_2, Y_4|Y_1, Y_3; \Sigma_{4b}) = (1-r^2)\begin{pmatrix}1 & 1/\sqrt{2}\\ 1/\sqrt{2} & 1\end{pmatrix},\\
&\mathrm{var}(Y_1, Y_4 \mid Y_2, Y_3; \Sigma_{4a}) = \mathrm{var}(Y_2, Y_3 \mid Y_1, Y_4; \Sigma_{4a})\\
&= \mathrm{var}(Y_1, Y_4 \mid Y_2, Y_3; \Sigma_{4b}) = \mathrm{var}(Y_2, Y_3 \mid Y_1, Y_4; \Sigma_{4b}) = \left(1 - \frac{1}{2-r^2}\right)\begin{pmatrix}1 & -r/\sqrt{2}\\ -r/\sqrt{2} & 1\end{pmatrix},\\
&\mathrm{var}(Y_3, Y_4 \mid Y_1, Y_2; \Sigma_{4a}) = \frac{2}{1-r^2}\begin{pmatrix}1 & 0\\ 0 & 1\end{pmatrix},\\
&\mathrm{var}(Y_3, Y_4 \mid Y_1, Y_2; \Sigma_{4b}) = \frac{1}{2}\begin{pmatrix}1 & r\\ r & 1\end{pmatrix}.
\end{aligned}$$

Consider $\mathrm{var}(Y_i \mid Y_k = y_k, Y_\ell = y_\ell)$. From the above, it is clear that all conditional variances are bounded below by some positive constant. It can be also seen that all conditional variances are bounded above as long as $1 - r^2 \geq C$ for some constant $C > 0$. Under Assumptions 1, $|r| \leq 1 - \varepsilon_r$ and thus $1 - r^2 \geq 1 - (1-\varepsilon_r)^2 > 0$.

Consider $\mathrm{E}\left(|Y_i| \mid Y_k = y_k, Y_\ell = y_\ell\right)$. If $i = j$ or $i = \ell$, then $\mathrm{E}\left(|Y_i| \mid Y_k = y_k, Y_\ell = y_\ell\right) = |y_i|$ and the result is immediate under Assumption 2. For $i \neq j, k$, let $\mathrm{E}(Y_i \mid Y_k = y_k, Y_\ell = y_\ell) = \mu_i$ and $\mathrm{var}(Y_i \mid Y_k = y_k, Y_\ell = y_\ell) = \sigma_i^2$, where detailed expressions are given above. Then, by Lemma A.27, we have that

$$\begin{aligned}
\mathrm{E}\left(|Y_i| \mid Y_k = y_k, Y_\ell = y_\ell\right) &= \left[\sigma_i\sqrt{\frac{2}{\pi}}\exp\left(-\frac{\mu_i^2}{2\sigma_i^2}\right) + \mu_i\left\{1 - 2\Phi\left(-\frac{\mu_i}{\sigma_i}\right)\right\}\right]\\
&\leq \sigma_i\sqrt{\frac{2}{\pi}} + |\mu_i|.
\end{aligned}$$

We can see from the above conditional means that, under Assumptions 1 and 2, $|\mu_i|$ is bounded above by some positive constant. As we already showed that σ_i^2 is bounded above, the proof is complete. ■

Lemma A.24 *Let* $Y \sim \mathrm{N}_4(0, \Sigma_4)$, *where* $\Sigma_4 = \Sigma_{4a}$ *or* $\Sigma_4 = \Sigma_{4b}$. *Also let* Y_{-i} *be the 3-dimensional random vector without the* i*th component and* $y_{-i} = (y_j, y_k, y_\ell)^\top$ *be its realization such that*

$$y_m = \begin{cases} -\Delta_j, & \text{if} \quad m = 1;\\ -\Delta_k, & \text{if} \quad m = 2;\\ 0, & \text{otherwise.}\end{cases}$$

Then, under Assumptions 1 and 2, for any $1 \leq i \leq 4$,

$$\mathrm{E}\left(|Y_i| \mid Y_{-i} = y_{-i}\right) \leq C$$

for some constant $C > 0$.

Proof It follows by the conditional mean and variance formulas of the multivariate Gaussian distribution that, regardless of $\Sigma_4 = \Sigma_{4a}$ or $\Sigma_4 = \Sigma_{4b}$,

$$\begin{aligned}
\mathrm{var}(Y_i \mid Y_{-i} = y_{-i}; \Sigma_4) &= \frac{(1-r^2)}{2}, \quad i = 1, \ldots, 4, \\
\mathrm{E}(Y_1 \mid Y_{-1} = y_{-1}; \Sigma_4) &= -\Delta_k r, \\
\mathrm{E}(Y_2 \mid Y_{-2} = y_{-2}; \Sigma_4) &= -\Delta_j r, \\
\mathrm{E}(Y_3 \mid Y_{-3} = y_{-3}; \Sigma_4) &= -\frac{\Delta_j - \Delta_k r}{\sqrt{2}}, \\
\mathrm{E}(Y_4 \mid Y_{-4} = y_{-4}; \Sigma_4) &= -\frac{\Delta_k - \Delta_j r}{\sqrt{2}}.
\end{aligned}$$

Under Assumptions 1 and 2, the absolute values of the conditional means and conditional variances are bounded above by $\sqrt{2}M$ and $1/2$, respectively. Then the result follows by Lemma A.27. ■

Lemma A.25 *Let $y \sim \mathrm{N}_4(\mathbf{0}, \Sigma_4)$, where $\Sigma_4 = \Sigma_{4a}$ or $\Sigma_4 = \Sigma_{4b}$. Then, for any $1 \le k < \ell \le 4$ and $1 \le i < j \le 4$,*

$$\mathrm{E}\left\{|Y_i Y_j| \;\middle|\; Y_k = y_k, Y_\ell = y_\ell\right\} \le C$$

for some constant $C > 0$.

Proof Let $I = \{k, \ell\}$ and write $y_I = (y_k, y_\ell)^\top = (y_{I_1}, y_{I_2})^\top$. We prove this lemma by considering the following three cases: $\mathrm{card}(\{i,j\} \cap I) = 0, 1, 2$, namely cases 1, 2, and 3, respectively.

Case 1: Consider the case $\mathrm{card}(\{i,j\} \cap I) = 0$. Let

$$\begin{aligned}
\mathrm{E}(Y_i \mid Y_I = y_I) = \mu_i, \quad \mathrm{var}(Y_i \mid Y_I = y_I) = \sigma_i^2, \\
\mathrm{E}(Y_j \mid Y_I = y_I) = \mu_j, \quad \mathrm{var}(Y_j \mid Y_I = y_I) = \sigma_j^2,
\end{aligned}$$

whose detailed expressions are provided in Lemma A.23. Also, let $Z_i = Y_i/(2^{1/2}\sigma_i) \sim \mathrm{N}(\mu_i/(2^{1/2}\sigma_i), 1/2)$ and $Z_j = Y_j/(2^{1/2}\sigma_j) \sim \mathrm{N}(\mu_j/(2^{1/2}\sigma_j), 1/2)$ and write

$$\begin{aligned}
|Y_i Y_j| = 2\sigma_i\sigma_j \left|\frac{Y_i}{\sqrt{2}\sigma_i}\frac{Y_j}{2^{1/2}\sigma_j}\right| &= 2\sigma_i\sigma_j \left|\frac{1}{4}(Z_i + Z_j)^2 - \frac{1}{4}(Z_i - Z_j)^2\right| \\
&\le 2\sigma_i\sigma_j \left\{\frac{1}{4}(Z_i + Z_j)^2 + \frac{1}{4}(Z_i - Z_j)^2\right\},
\end{aligned}$$

where the last inequality holds by the triangle inequality. We have that $(Z_i+Z_j)^2$ and $(Z_i - Z_j)^2$ follow non-central $\chi^2_{\mathrm{df}=1}$ distributions with non-centrality parameters $\lambda_+ = \mu_i^2/(2\sigma_i^2) + \mu_j^2/(2\sigma_j^2)$ and $\lambda_- = \mu_i^2/(2\sigma_i^2) - \mu_j^2/(2\sigma_j^2)$, and thus,

$$\mathrm{E}\left\{|Y_i Y_j| \mid Y_{I_1} = y_{I_1}, Y_{I_2} = y_{I_2}\right\} \le \frac{\sigma_i\sigma_j}{2}\left\{\lambda_+ + \lambda_- + 2\right\}.$$

By Lemma A.23, we have $\mathrm{E}\left\{|Y_iY_j| \mid Y_{I_1}=y_{I_1}, Y_{I_2}=y_{I_2}\right\} < C$ for some constant $C > 0$.

Case 2: For the case $\mathrm{card}\left(\{i,j\}\cap I\right) = 1$, we assume, without loss of generality that, $\{i,j\}\cap I = \{i\}$ and write

$$\begin{aligned}\mathrm{E}\left\{|Y_iY_j| \mid Y_{I_1}=y_{I_1}, Y_{I_2}=y_{I_2}\right\} &= |y_{I_1}|\ \mathrm{E}\left\{|Y_j| \mid Y_{I_1}=y_{I_1}, Y_{I_2}=y_{I_2}\right\}\\ &\le M\ \mathrm{E}\left\{|Y_j| \mid Y_{I_1}=y_{I_1}, Y_{I_2}=y_{I_2}\right\},\end{aligned}$$

where $|y_k| \le M$ by Assumption 2. Then, by Lemma A.23, we have

$$\mathrm{E}\left\{|Y_iY_j| \mid Y_{I_1}=y_{I_1}, Y_{I_2}=y_{I_2}\right\} \le C$$

for some constant $C > 0$.

Case 3: For the case $\{i,j\}\cap I = \{i,j\}$, Assumption 2 gives that

$$\mathrm{E}\left\{|Y_iY_j| \mid Y_{I_1}=y_{I_1}, Y_{I_2}=y_{I_2}\right\} = |y_{I_1}y_{I_2}| \le M^2.$$

This conclude the proof. ■

Lemma A.26 *Let $h_{ij}(r) = \partial\Phi(a_1,\ldots,a_4;\Sigma_4)/\partial\rho_{ij}(r)$, where $\Sigma_4 = [\rho_{ij}(r)]_{1\le i,j\le 4}$. Then, for any $1 \le i < j \le 4$ and $1 \le k < \ell \le 4$, and some constant $C > 0$,*

$$\left|\frac{\partial h_{ij}(r)}{\partial \rho_{k\ell}}\right| \le C.$$

Proof Let $I = \{i,j\}$, $K = \{k,\ell\}$. We write $x_I = (x_i,x_j)^\top = (x_{I_1},x_{I_2})^\top$ and $\mathcal{R}_I = \{(x_i,x_j) \mid x_i < a_i, x_j < a_j\} \subset \mathbb{R}^2$, where.

$$a_m = \begin{cases}-\Delta_j, & \text{if} \quad m=1;\\ -\Delta_k, & \text{if} \quad m=2;\\ 0, & \text{otherwise.}\end{cases}$$

We consider three cases where $\mathrm{card}(I\cap K) = 2,1,0$.

Consider the case $\mathrm{card}(I\cap K) = 0$, i.e., $K = \{1,\ldots,4\} - I = I^c$. By Plackett (1954)

$$\frac{\partial h_I(r)}{\partial \rho_{I^c}} = \frac{\partial}{\partial \rho_{I^c}}\int_{\mathcal{R}_{I^c}} \phi(a_I, x_{I^c};\Sigma_4)dx_{I^c} = \phi(a_I,a_{I^c};\Sigma_4) \le |\Sigma_4|^{1/2}$$

because $\phi(a_I,a_{I^c};\Sigma_4) \le \phi(0,0,0,0;\Sigma_4) = |\Sigma_4|^{-1/2}$. By Lemma A.20, we have

$$\left|\frac{\partial h_I(r)}{\partial \rho_{I^c}}\right| \le C$$

for some constant $C > 0$.

Consider the case $\mathrm{card}(I\cap K) = 2$, i.e., $I = K$. For notational convenience, we write $a_I = y_I$ and $x_{I^c} = y_{I^c}$. Then,

$$\begin{aligned}\frac{\partial h_I(r)}{\partial \rho_I} &= \frac{\partial}{\partial \rho_I}\int_{\mathcal{R}_{I^c}} \phi(a_I, x_{I^c};\Sigma_4)\mathrm{d}x_{I^c} = \frac{\partial}{\partial \rho_I}\int_{\mathcal{R}_{I^c}} \phi(y_I, y_{I^c};\Sigma_4)\mathrm{d}y_{I^c}\\ &= \int_{\mathcal{R}_{I^c}} \frac{\partial}{\partial \rho_I}\phi(y_I,y_{I^c};\Sigma_4)\mathrm{d}y_{I^c} = \int_{\mathcal{R}_{I^c}} \frac{\partial^2}{\partial y_{I_1}\partial y_{I_2}}\phi(y_I,y_{I^c};\Sigma_4)\mathrm{d}y_{I^c},\end{aligned}$$

where the last equality is due to Plackett (1954). Let $\omega_j^\top$ be the jth row of $\Sigma_4^{-1} = [\omega_{ij}]_{1\le i,j\le 4}$, $\Sigma_I = \mathrm{var}(y_I)$, and $\Sigma_{I^c|I} = \mathrm{var}(y_{I^c}|y_I)$. By differentiating the multivariate Gaussian density, we have

$$\left|\int_{\mathcal{R}_{I^c}} \frac{\partial^2}{\partial y_{I_1}\partial y_{I_2}}\phi(y_I, y_{I^c};\Sigma_4)dy_{I^c}\right| = \left|\int_{\mathcal{R}_{I^c}} \left\{(\omega_{I_1}^\top y)(\omega_{I_2}^\top y) - \omega_I\right\}\phi(y_I, y_{I^c};\Sigma_4)dy_{I^c}\right|$$
$$= \left|\phi(y_I;\Sigma_I)\int_{\mathcal{R}_{I^c}} \left\{(\omega_{I_1}^\top y)(\omega_{I_2}^\top y) - \omega_I\right\}\phi(y_{I^c}|y_I;\Sigma_{I^c|I})dy_{I^c}\right|.$$

We also have that $\phi(y_I;\Sigma_I) \le \phi(0,0;\Sigma_I) = |\Sigma_I|^{-1/2} \le |\Sigma_4|^{-1/2}$ for any I, and by Lemma A.20, $|\Sigma_4|^{-1/2}| \le C_2^{1/2}$ for some constant $C_2 > 0$. Thus,

$$\left|\int_{\mathcal{R}_{I^c}} \frac{\partial^2}{\partial y_{I_1}\partial y_{I_2}}\phi(y_I, y_{I^c};\Sigma_4)dy_{I^c}\right| \le C_2^{1/2}\left|\int_{\mathcal{R}_{I^c}} \left\{(\omega_{I_1}^\top y)(\omega_{I_2}^\top y) - \omega_I\right\}\phi(y_{I^c}|y_I;\Sigma_{I^c|I})dy_{I^c}\right|.$$

The absolute value of the integral of the last term is bounded as

$$\left|\int_{\mathcal{R}_{I^c}} \left\{(\omega_{I_1}^\top y)(\omega_{I_2}^\top y) - \omega_I\right\}\phi(y_{I^c}|y_I;\Sigma_{I^c|I})dy_{I^c}\right| \le \int_{\mathcal{R}_{I^c}} \left|(\omega_{I_1}^\top y)(\omega_{I_2}^\top y) - \omega_I\right|\phi(y_{I^c}|y_I;\Sigma_{I^c|I})dy_{I^c}$$
$$\le \int_{\mathbb{R}^2} \left|(\omega_{I_1}^\top y)(\omega_{I_2}^\top y) - \omega_I\right|\phi(y_{I^c}|y_I;\Sigma_{I^c|I})dy_{I^c}$$
$$\le \int_{\mathbb{R}^2} \left|(\omega_{I_1}^\top y)(\omega_{I_2}^\top y)\right|\phi(y_{I^c}|y_I;\Sigma_{I^c|I})dy_{I^c} + |\omega_I|,$$

where the second inequality is due to expanding the range of integration, and the third inequality is due to the triangle inequality. By Lemma A.20, we know that, whether $\Sigma_4 = \Sigma_{4a}$ or $\Sigma_4 = \Sigma_{4b}$, $|\omega_{jk}| \le C_1$, for all $1 \le j,k \le 4$. Also, by the triangle inequality,

$$\left|(\omega_{I_1}^\top y)(\omega_{I_2}^\top y)\right| = \left|\sum_{i'=1}^4\sum_{j'=1}^4 \omega_{I_1 i'}\omega_{I_2 j'}y_{i'}y_{j'}\right| \le C_1^2\sum_{i'=1}^4\sum_{j'=1}^4 \left|y_{i'}y_{j'}\right|.$$

Hence, for some constant $C > 0$,

$$\left|\frac{\partial h_I(r)}{\partial \rho_I}\right| \le C_2^{1/2}\int_{\mathbb{R}^2}\left|(\omega_{I_1}^\top y)(\omega_{I_2}^\top y)\right|\phi(y_{I^c}|y_I;\Sigma_{I^c|I})dy_{I^c} + C_2^{1/2}C_1$$
$$\le C_2^{1/2}C_1^2\sum_{i'=1}^4\sum_{j'=1}^4\int_{\mathbb{R}^2}\left|y_{i'}y_{j'}\right|\phi(y_{I^c}|y_I;\Sigma_{I^c|I})dy_{I^c} + C_2^{1/2}C_1$$
$$\le C,$$

where the last inequality holds by Lemma A.25.

Consider the case card$(I\cap K) = 1$. We assume, without loss of generality, that $I = \{i,j\}$ and $K = \{j,\ell\}$, i.e., $I \cap K = \{j\}$. Then, by Plackett (1954),

$$\frac{\partial h_{ij}(r)}{\partial \rho_{j\ell}} = \frac{\partial}{\partial \rho_{j\ell}}\int_{-\infty}^{a_\ell}\int_{-\infty}^{a_k}\phi(a_i,a_j,x_k,x_\ell;\Sigma_4)\mathrm{d}x_k\mathrm{d}x_\ell = \int_{-\infty}^{a_k}\frac{\partial}{\partial a_j}\phi(a_i,a_j,x_k,a_\ell;\Sigma_4)\mathrm{d}x_k.$$

For notational convenience, let $y = (a_i, a_j, x_k, a_\ell)^\top = (y_i, y_j, y_k, y_\ell)^\top$ and write

$$\frac{\partial h_{ij}(r)}{\partial \rho_{j\ell}} = \int_{-\infty}^{a_k} \frac{\partial}{\partial y_j} \phi(y; \Sigma_4) \mathrm{d}y_k = \int_{-\infty}^{a_k} (-\omega_j^\top y) \phi(y; \Sigma_4) \mathrm{d}y_k.$$

Then, we have that

$$\begin{aligned}
\left| \frac{\partial h_{ij}(r)}{\partial \rho_{j\ell}} \right| &= \left| \int_{-\infty}^{a_k} (-\omega_j^\top y) \phi(y; \Sigma_4) \mathrm{d}y_k \right| \\
&\leq \int_{-\infty}^{a_k} |\omega_j^\top y| \phi(y; \Sigma_4) \mathrm{d}y_k \\
&\leq \int_{-\infty}^{\infty} |\omega_j^\top y| \phi(y; \Sigma_4) \mathrm{d}y_k \quad \text{(by expanding the range of integration)} \\
&\leq \sum_{i' \neq k} |\omega_{ji'} y_{i'}| \phi(y_i, y_j, y_\ell; \Sigma_3) + \phi(y_i, y_j, y_\ell; \Sigma_3) \int_{-\infty}^{\infty} |\omega_{jk} y_k| \phi(y_k | y_i, y_j, y_\ell; \Sigma_4) \mathrm{d}y_k,
\end{aligned}$$

where the last inequality holds by the triangle inequality. By Lemma A.20, $|\omega_{jk}| \leq C_1$ for all $1 \leq j, k \leq 4$, and by Assumption 2, $|y_i| \leq M$ for all for $1 \leq i \leq 4$. This gives

$$\begin{aligned}
\left| \frac{\partial h_{ij}(r)}{\partial \rho_{j\ell}} \right| &\leq \sum_{i' \neq k} |\omega_{ji'} y_{i'}| \phi(y_i, y_j, y_\ell; \Sigma_3) + \int_{-\infty}^{\infty} |\omega_{jk} y_k| \phi(y_k | y_i, y_j, y_\ell; \Sigma_4) \mathrm{d}y_k \left\{ \phi(y_i, y_j, y_\ell; \Sigma_3) \right\} \\
&\leq 3 C_1 M |\Sigma_4|^{-1/2} + C_1 |\Sigma_4|^{-1/2} \int_{-\infty}^{\infty} |y_k| \phi(y_k | y_i, y_j, y_\ell; \Sigma_4) \mathrm{d}y_k.
\end{aligned}$$

Again, by Lemma A.20, $|\Sigma_4|^{-1/2} \leq C_2^{1/2}$, and by Lemma A.24, above integral is bounded above by some positive constant. Thus, for some constant $C > 0$

$$\left| \frac{\partial h_{ij}(r)}{\partial \rho_{j\ell}} \right| \leq C.$$

■

Lemma A.27 *Let* $X \sim \mathrm{N}(\mu, \sigma^2)$. *Then* $\mathrm{E}(|X|) \leq \sigma (2/\pi)^{1/2} + |\mu|$.

Proof For $X \sim \mathrm{N}(\mu, \sigma^2)$, $|X|$ follows the folded Gaussian distribution with mean

$$\mathrm{E}(|X|) = \sigma \left(\frac{2}{\pi} \right)^{1/2} \exp\left(-\frac{\mu^2}{2\sigma^2} \right) + \mu \left\{ 1 - 2\Phi\left(-\frac{\mu}{\sigma} \right) \right\}.$$

Since $\exp(-a^2) \leq 1$ and $0 \leq a\{1 - 2\Phi(a)\} \leq |a|$, $a \in \mathbb{R}$, we have that

$$\mathrm{E}(|X|) \leq \sigma \left(\frac{2}{\pi} \right)^{1/2} + |\mu|.$$

■
